# Exploring the Redox Properties of Bench-Stable Uranyl(VI) Diamido–Dipyrrin Complexes

**DOI:** 10.1021/acs.inorgchem.1c03744

**Published:** 2022-02-07

**Authors:** Karlotta van Rees, Emma K. Hield, Ambre Carpentier, Laurent Maron, Stephen Sproules, Jason B. Love

**Affiliations:** † EaStCHEM School of Chemistry, The University of Edinburgh, Edinburgh EH9 3FJ, U.K.; ‡ Laboratoire de Physique et Chimie de Nano-Objets, Institut National des Sciences Appliquées, Université de Toulouse, 135 avenue de Rangueil, 31077 Toulouse Cedex 4, France; § WestCHEM School of Chemistry, University of Glasgow, Glasgow G12 8QQ, U.K.

## Abstract

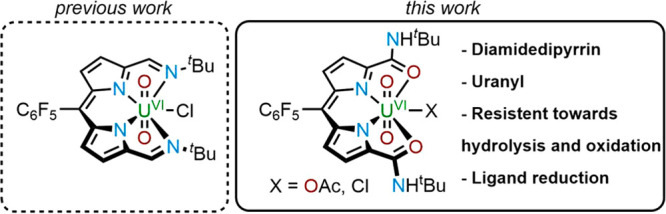

The uranyl complexes UO_2_(OAc)(**L**) and UO_2_Cl(**L**) of the redox-active, acyclic diamido–dipyrrin anion **L**^**–**^ are reported and their redox properties explored. Because of the inert nature of the complexes toward hydrolysis and oxidation, synthesis of both the ligands and complexes was conducted under ambient conditions. Voltammetric, electron paramagnetic resonance spectroscopy, and density functional theory studies show that one-electron chemical reduction by the reagent CoCp_2_ leads to the formation of a dipyrrin radical for both complexes [Cp_2_Co][UO_2_(OAc)(**L**^•^)] and [Cp_2_Co][UO_2_Cl(**L**^•^)].

## Abstract 2

Air-stable uranyl complexes of diamido−dipyrrin ligands undergo one-electron reduction to form highly air-sensitive ligand radical complexes instead of uranyl(V) complexes seen for diimine analogues.

## Introduction

Redox-active ligands, also referred to as redox-noninnocent ligands, continue to fascinate and perplex chemists. While the ability of these ligands to adopt multiple stable oxidation states often hinders analysis of the electronic structures of metal complexes, the reactivity of metals can be expanded by their action as electron reservoirs, altered Lewis acids, and reactive ligand radicals and in enabling ligand-to-substrate electron transfer.^1−3^ Although the chemistry with transition metals has been vastly explored, there has only recently been a rise in interest of actinide complexes of redox-active ligands, in particular those of uranium.^4−6^

Uranium is most commonly present as the uranyl(VI) dication UO_2_^2+^ under ambient conditions. This dioxide adopts a linear [O≡U^VI^≡O]^2+^ structure in which the axial oxygen atoms (O_ax_) are strongly bound to the uranium center.^7^ As a result, UO_2_^2+^ is very stable in terms of both kinetics and thermodynamics. Even so, the reduction of uranyl(VI) to uranium(IV) via the unstable uranyl(V) cation UO_2_^+^ is an important aspect of uranium remediation by immobilization, and significant advances have been made in the isolation and study of reduced uranyl complexes, e.g., in oxometalated and oxosilylated uranyl(V) compounds.^8^

**Figure 1:**
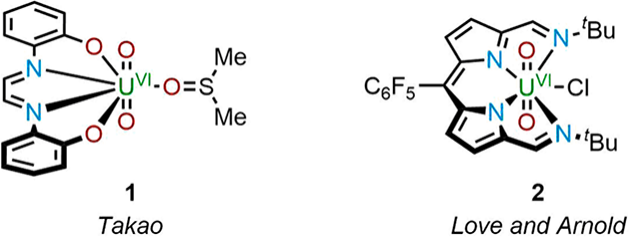
Structures of uranyl(VI) di-imine and dipyrrin complexes.

Uranyl complexes of redox-active ligands, such as Schiff bases,^9,10^ quinones,^4^ and pyrroles in, for example, tetraaza[14]annulenes,^11^ calix[4]pyrroles,^12^ and dipyrrins,^13−15^ have been reported. Because of the added redox character of these ligands, the complexes react differently under reducing conditions. For example, uranyl(VI) complexes of pentadentate N_3_O_2_-saldien ligands with various substituents all underwent one-electron uranium reduction to afford the corresponding uranyl(V) complex, regardless of the difference in the substituents.^16^ In contrast, the uranyl(VI) α-di-imine diphenolate (**1**) (Figure 1) and uranyl(VI) salophens undergo one-electron reduction of the ligand, leading to ligand-centered radical anions and not the expected uranyl(V) complexes.^9,10,17^

Dipyrrins are popular because of their effective absorption of visible light through π–π* transitions, forming colorful and luminescent metal complexes.^18,19^ Uranyl complexes of dipyrrin ligands can be readily accessed through anhydrous, salt metathesis routes.^13^ We recently reported the redox behavior of the donor-expanded Schiff-base uranyl(VI) dipyrrin complex **2** (Figure 1) and its contrasting but controlled inner- and outer-sphere redox chemistry. The use of 1 equiv of the outer-sphere reductant CoCp_2_ resulted in one-electron reduction of the ligand to a dipyrrin radical. The addition of a second equiv of CoCp_2_ reduced the uranium center to uranyl(V). The reaction of **2** with 1 equiv of the inner-sphere reductant [TiCp_2_Cl]_2_ led to the formation of a doubly titanated uranium(IV) complex.^14^ In addition, the effects of both the equatorial coordination sphere and axial oxo–ligand bonding in **2** were investigated, showing that it is possible to shift the nonaqueous uranyl(VI/V) and uranyl(V/IV) reduction potentials to values in the range accessible to reductants that are present in uranium remediation processes and in nuclear fuel storage.^15^ However, these dipyrrin complexes all display air sensitivity and therefore need to be handled accordingly.

This study presents the formation of easy-to-synthesize and bench-stable uranyl complexes of a diamidodipyrrin ligand and an evaluation of their reduction properties. A similar ligand has previously been exploited in the formation of boron and transition-metal complexes, such as nickel, copper, and cobalt, although these studies mainly focused on the rich coordination chemistry of these ligands.^20−22^ We rationalized that the use of these ligands would deliver a uranyl complex that would potentially be resistant toward oxidation reactions and hydrolysis, while maintaining its redox properties.

## Results and Discussion

### Synthesis and Structures of Uranyl(VI) Complexes

**Scheme 1:**
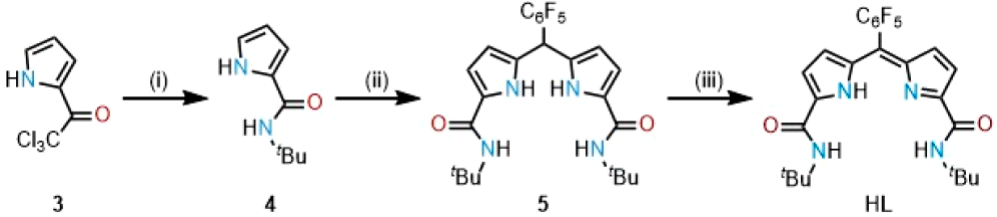
Synthesis of H**L** Reaction conditions: (i) neat ^*t*^BuNH_2_, reflux, 16 h; (ii) 0.5 equiv of C_6_F_5_CHO, 5 mol % *p*-TSA, PhCH_3_, reflux, 16 h; (iii) 1.1 equiv of DDQ, THF, RT, 24 h.

The synthesis of H**L** was achieved using a modification of previously reported procedures (Scheme 1).^20^ The amination of (trichloroacetyl)pyrrole was conducted in neat, boiling *tert*-butylamine; however, because of the steric demand of *tert*-butylamine, the pyrrole amide **4** was synthesized in lower yield compared with the literature derivatives. The second step was an acid-catalyzed condensation that led to formation of the dipyrromethane **5** in 36% yield. In contrast to acyclic Schiff-base dipyrrin ligands made previously in our group, **5** did not spontaneously oxidize during its synthesis and required additional oxidant (2,3-dichloro-5,6-dicyano-1,4-benzoquinone, DDQ) to form the dipyrrin H**L**, which was readily purified using silica chromatography.^23^ The formation of H**L** was indicated not only by the disappearance of the *meso*-proton singlet at 5.86 ppm in the ^1^H NMR spectrum but also by the intensely orange solid obtained, typical of the dipyrrin chromophore (see the Supporting Information, SI).

**Scheme 2:**
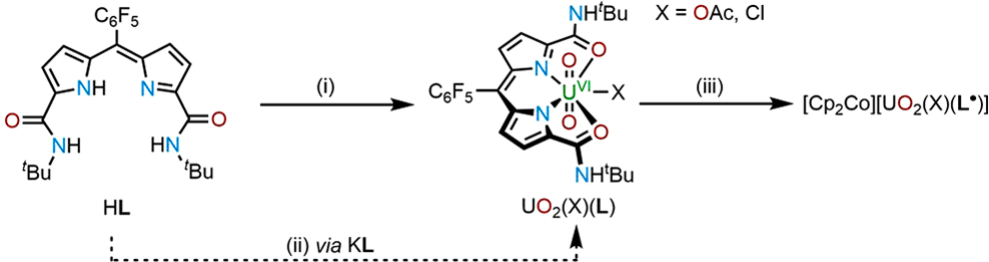
Synthesis of Uranyl Complexes of H**L** Reaction conditions: (i) 1.1 equiv of UO_2_(OAc)_2_·2H_2_O or UO_2_Cl_2_(THF)_2_, NEt_3_, MeOH/CHCl_3_ (1:3, v/v), heated to reflux in air for 16 h; (ii) 1.5 equiv of KH under N_2_, THF, RT, 16 h, followed by the addition of UO_2_Cl_2_(THF)_2_; (iii) 1 equiv of CoCp_2_, THF, RT, 16 h.

The reaction between H**L**, triethylamine, and 1 equiv of uranyl acetate [UO_2_(OAc)_2_·2H_2_O] or uranyl chloride [UO_2_Cl_2_(THF)_2_] (THF = tetrahydrofuran) in a mixture of methanol (MeOH) and CHCl_3_ (1:3, v/v) in air led to rapid color changes from an orange to a dark-pink solution (Scheme 2). The acetate complex UO_2_(OAc)(**L**) was obtained in 77% yield as a dark-pink solid, and the chloride UO_2_Cl(**L**) was obtained in 91% yield as a dark-reddish-pink solid after aqueous workups. While no additional purification steps were required for UO_2_(OAc)(**L**), UO_2_Cl(**L**) was heated in chloroform to ensure the formation of a single product. The second product is likely the ion pair [UO_2_(solvent)(**L**)][Cl] formed through ready dissociation of the chloride anion.^15^ The chloride complex UO_2_Cl(**L**) may also be prepared via K**L** using air-sensitive methods.

Formation of the uranyl complexes was indicated by the disappearance of the pyrrole N–H proton at 12.69 ppm for H**L** and the downfield shift of the pyrrole peaks in the ^1^H NMR spectra (see the SI).^24^ Both complexes adopt *C*_2*h*_ symmetry in solution, which is also seen in the ^19^F NMR spectra, with three resonances indicating horizontal planar symmetry. In addition, the ^1^H NMR spectrum of UO_2_(OAc)(**L**) contains a broad singlet at 2.17 ppm with an integration of 3H that is assigned to the coordinated acetate ion; this fluxionality of the acetate means that it is not easily identified in the ^13^C{^1^H} NMR spectrum. The chloride complex UO_2_Cl(**L**) was also prepared under nonaqueous conditions: the reaction between K**L** (formed *in situ* by the reaction of H**L** and KH in THF) and UO_2_Cl_2_(THF)_2_ in THF formed UO_2_Cl(**L**) in high yield.

**Figure 2:**
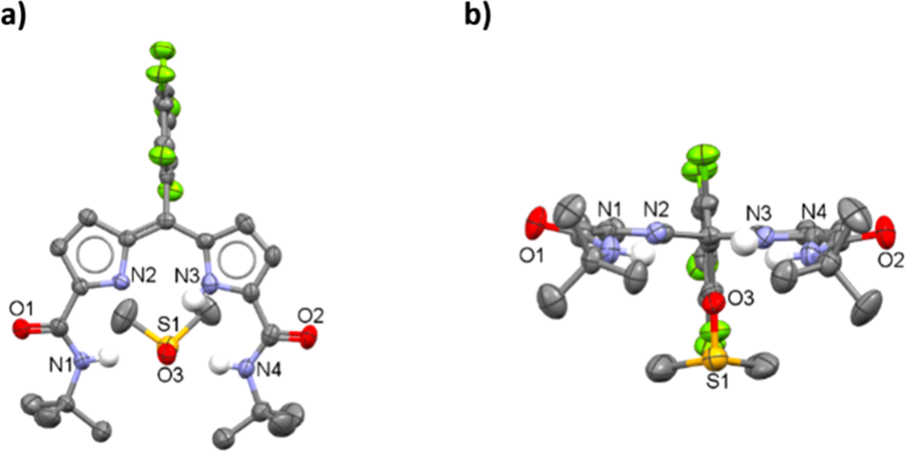
X-ray crystal structure of H**L** viewed from the side and top. For clarity, all hydrogen atoms except those involved in hydrogen bonding are omitted (displacement ellipsoids are drawn at 50% probability).

**Figure 3:**
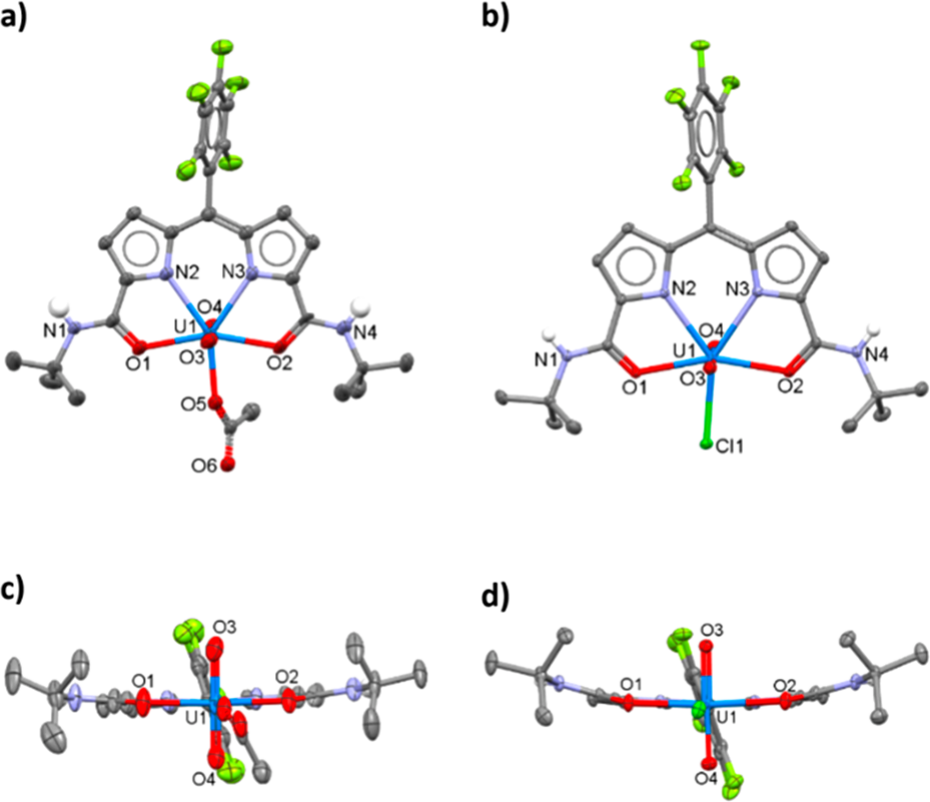
X-ray crystal structures of UO_2_(OAc)(**L**) (a and c) and UO_2_Cl(**L**) (b and d) viewed from the side and top. For clarity, all hydrogen atoms except on amide N1 and N4 are omitted (displacement ellipsoids drawn at 50% probability).

Crystals suitable for X-ray analysis were grown for H**L**, UO_2_(OAc)(**L**) and UO_2_Cl(**L**) (Figures 2 and 3). Weakly diffracting orange plates of H**L** were crystallized from a concentrated dimethyl sulfoxide (DMSO) solution, and so the X-ray structure is reported to show connectivity only. H**L** did not display any intermolecular hydrogen bonding and instead displayed hydrogen bonding between the amide N4-H and the O3 atom of the DMSO solvate molecule.

Greenish-pink blocks of UO_2_(OAc)(**L**) were grown through the slow evaporation of a concentrated THF solution. The asymmetric unit comprises two molecules that differ primarily in the orientation of the monodentate acetate group, supporting the fluxionality of this anion seen in solution by NMR spectroscopy. In the solid state, the complex adopts a distorted pentagonal-bipyrimidal coordination geometry, in which the ONNO donor set of the expanded dipyrrin ligand occupies the equatorial positions along with the acetate ligand. This shows ONNO coordination geometry similar to that of Cu(DADP^ph,*i*pr^)Cl (DADP^ph,*i*pr^ = 1,1′-isopropylamide-5-phenyl-4,6-dipyrrinato) in which the equatorial position is occupied by a chloride ligand.^20^ The uranium coordinates to the oxygen atoms of the amide groups, as seen with other uranyl(VI) amide complexes.^25^ The O_ax_4–U1 and U1–O_ax_3 bonds are 1.750(6) and 1.762(5) Å, respectively, with an O_ax_4–U1–O_ax_3 angle of 177.9(2)° and are fully consistent with uranyl(VI). The U1–N_pyrrole_ bond lengths are 2.514(5) and 2.505(6) Å, the U1–O_amide_ bond lengths are 2.315(1) Å, and the U1−O5_acetate_ is 2.314(5) Å.

Pink crystals of UO_2_Cl(**L**) were grown by the slow evaporation of a THF solution, and the X-ray crystal structure is similar to that of UO_2_(OAc)(**L**). In this case, the O_ax_4–U1 and U1–O_ax_3 bonds are 1.774(2) and 1.759(2) Å, respectively, with an O_ax_4–U1–O_ax_3 angle of 177.54(9)°. The U1–N_pyrrole_ bond lengths are 2.508(2) and 2.517(2) Å, while the U1–O_amide_ bond lengths are 2.389(2) and 2.406(2) Å. The U1–Cl1 bond length is 2.7018(6) Å, which is close to the U–Cl bond length of the dipyrrin–diimine analogue **2**, 2.710(1) Å.^14^ Both complexes exhibit U–O_ax_ bond lengths and O_ax_–U–O_ax_ angles in the range of other unfunctionalized uranyl(VI) complexes, in which an average U–O_ax_ bond of 1.777 Å is seen.^8^ In addition, the U–O_amide_ bond distance is similar to those found in other uranyl(VI) amide complexes (typically 2.34–2.40 Å).^25^

### Electrochemistry

**Figure 4:**
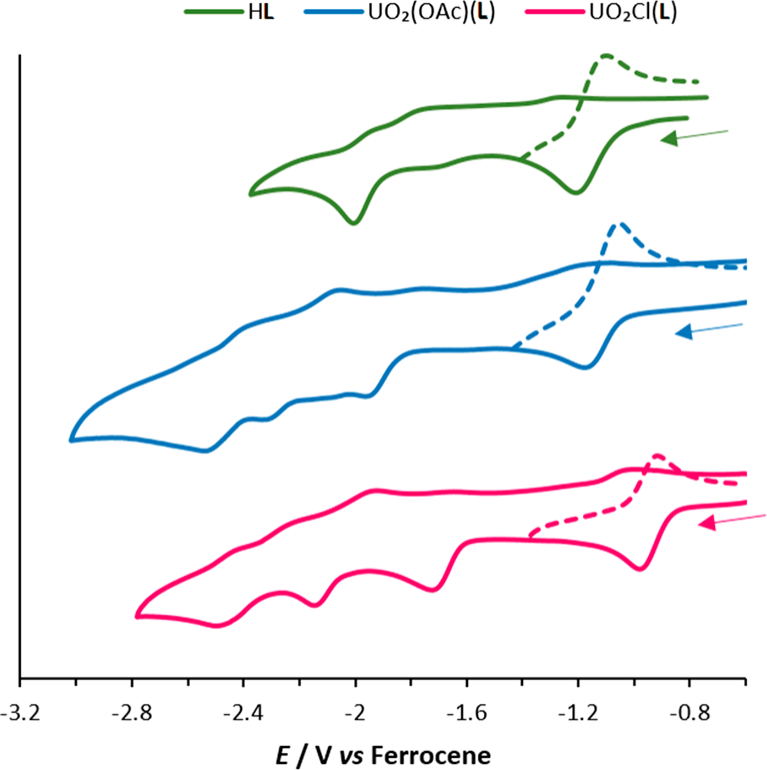
Stacked CVs for H**L**, UO_2_(OAc)(**L**) and UO_2_Cl(**L**). All measured as 1 mM MeCN solutions (0.1 M [^*n*^Bu_4_N][PF_6_] supporting electrolyte, glassy-carbon working electrode, platinum gauze counter electrode, and silver wire quasi-reference electrode). Potentials are referenced against the Fc/Fc^+^ couple recorded under identical conditions.

The cyclic voltammograms (CVs) of H**L**, UO_2_(OAc)(**L**), and UO_2_Cl(**L**) were recorded in acetonitrile (MeCN) at a scan rate of 100 mV s^–1^ (Figure 4). The CV of H**L** features a quasi-reversible reduction at *E*_1/2_ = −1.15 V versus ferrocene/ferrocenium (Fc/Fc^+^) and an irreversible reduction at *E*_p_ = −1.99 V versus Fc/Fc^+^. The first reduction appears reversible when isolated in the CV (Figure 4, dotted line). This feature is significantly less negative than that of the analogous diimine–dipyrrin ligand (seen in **2**), which displays a reversible reduction at *E*_1/2_ = −1.51 V versus Fc/Fc^+^ in CH_2_Cl_2_.^14^ Although the diamide ligand is more easily reduced than the diimine analogue, this is not true of their corresponding complexes. The CV of UO_2_(OAc)(**L**) features four different redox processes upon cathodic scanning. The first is a quasi-reversible reduction process at *E*_1/2_ = −1.10 V versus Fc/Fc^+^, followed by irreversible reduction processes at *E*_p_ = −1.97, −2.31, and −2.53 V versus Fc/Fc^+^. The CV of UO_2_Cl(**L**) also features four different redox processes, the first a quasi-reversible reduction process at *E*_1/2_ = −0.88 V versus Fc/Fc^+^, followed by irreversible reduction processes at *E*_p_ = −1.72, −2.15, and −2.50 V versus Fc/Fc^+^. In contrast, the diimine–dipyrrin analogue **2** has two consecutive quasi-reversible reduction processes that are both more accessible at *E*_1/2_ = −0.97 and −1.18 V versus Fc/Fc^+^ compared with UO_2_(X)(**L**) (X = OAc, Cl). This variation may be due to an increase of the electron density from the amide oxygen atoms to the uranium in the uranyl complexes of **L**, making them less susceptible to reduction. In addition, the solution of UO_2_Cl(**L**) required additional stirring after each measurement because of the formation of a second species with a similar reduction pattern (see the SI), which may arise from chloride dissociation to form the ion pair [UO_2_(MeCN)(**L**)][Cl].

### One-Electron Reduction

Colbaltocene (CoCp_2_) is a strong outer-sphere reductant with a formal cobalt(III)/cobalt(II) redox potential of −1.33 V versus Fc/Fc^+^^26^ but could only be used to study the first reduction of UO_2_(OAc)(**L**) and UO_2_Cl(**L**) because of the significantly more negative second reduction potentials. Reactions between either UO_2_(OAc)(**L**) or UO_2_Cl(**L**) and 1 equivalent of CoCp_2_ in pyridine-*d*_5_ lead to a dark-red, NMR-silent compound (Scheme 2). Scale-ups were carried out in dry THF, causing the products to precipitate as greenish-brown solids, which are characterized as the ligand-reduction products [Cp_2_Co][UO_2_(OAc)(**L**^•^)] or [Cp_2_Co][UO_2_Cl(**L**^•^)], respectively. These complexes are highly sensitive toward air and react rapidly to form new compounds; unfortunately, we have been unable to identify the products of these reactions. Both reduced complexes were successfully characterized by elemental analysis, but attempts to obtain single crystals for X-ray structural characterization were unsuccessful.

### Electron Paramagnetic Resonance (EPR) Spectroscopy

The room temperature (RT) EPR spectra of [Cp_2_Co][UO_2_(OAc)(**L**^•^)] and [Cp_2_Co][UO_2_Cl(**L**^•^)] show a relatively sharp line devoid of hyperfine structure synonymous with the formation of an *S* = ^1^/_2_ species (see the SI). Both compounds show *g*_iso_ = 1.997, a value significantly shifted from that of the free electron (2.0023). These data are consistent with a ligand-centered reduction affording [UO_2_(X)(**L**^•^)]^−^, where the presence of the coordinated uranium(VI) ion not only has instigated the *g* shift but also broadened the line, obscuring all hyperfine splitting from the various spin-active nuclei in the dipyrrin.^14^ No signal for a uranyl(V) complex (*f*^1^) would be expected to be seen at RT.

### Electronic Spectroscopy

**Figure 5:**
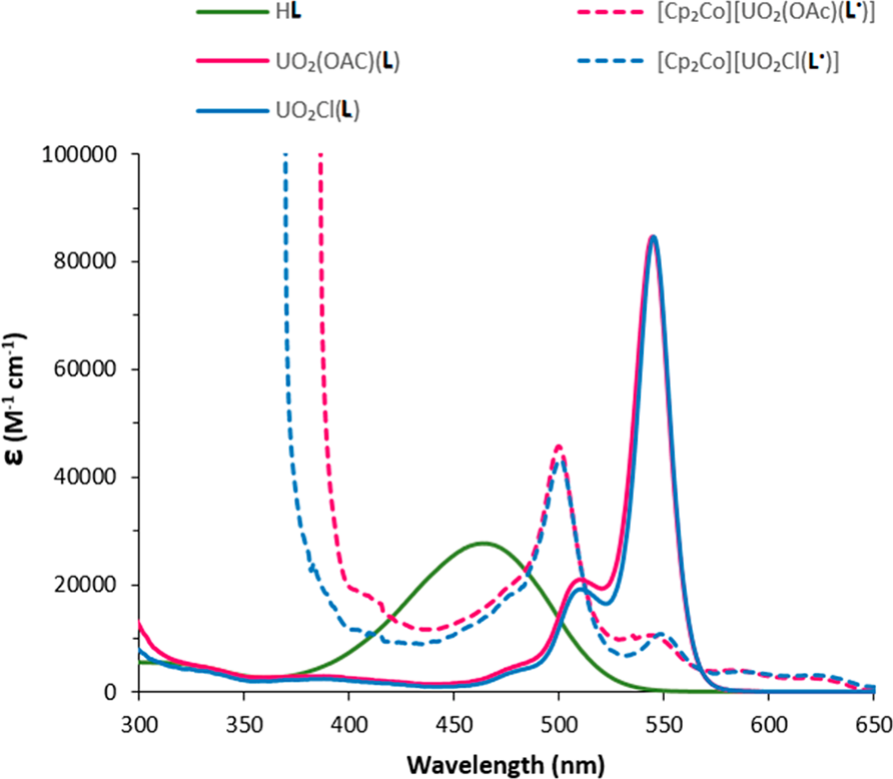
UV–vis spectra of H**L** in CH_2_Cl_2_, UO_2_(OAc)(**L**) and UO_2_Cl(**L**) in THF, and [Cp_2_Co][UO_2_(OAc)(**L**^•^)] and [Cp_2_Co][UO_2_Cl(**L**^•^)] in pyridine.

The absorbance spectra of H**L**, acetate, chloride uranyl complexes UO_2_(X)(**L**), and reduced complexes [Cp_2_Co][UO_2_(X)(**L**^•^)] were recorded (Figure 5). H**L** has a maximum absorbance of 470 nm (ε = 27280 M^–1^ cm^–1^) and is similar to the previously synthesized derivatives.^20^ Upon metalation to form the uranyl complexes UO_2_(X)(**L**), the easy-to-visualize color change is reflected in the UV–vis spectrum with significant red shifts observed relative to H**L**; the absorbance is independent of the anion, and both complexes exhibit a maximum absorbance at 546 nm (ε = 82316 M^–1^ cm^–1^) along with a second, weaker band at 510 nm and a shoulder at 478 nm. The reduced compounds [Cp_2_Co][UO_2_(X)(**L**^•^)] are poorly soluble in THF, and measurements were therefore carried out in pyridine. Both compounds exhibit near-identical spectra. The intense absorption of the dipyrrin chromophore in the UV and visible regions that took place before 300 nm has now shifted dramatically and can be seen just before 400 nm. The maximum absorbance has also shifted to 500 nm (ε = 45700 M^–1^ cm^–1^).

### Density Functional Theory (DFT) Calculations

**Figure 6:**
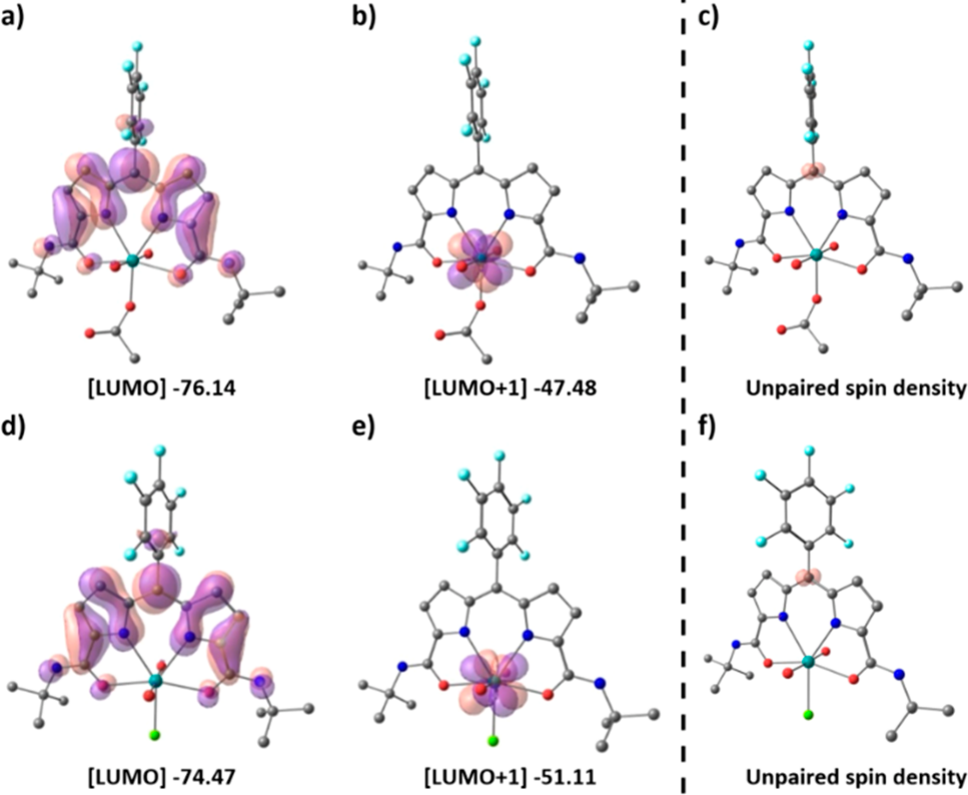
Molecular orbital plots of UO_2_(OAc)(**L**) (a and b) and UO_2_Cl(**L**) (d and e) and spin-density plots of the singly reduced complexes [UO_2_(X)(**L**^•^)]^−^ (c and f). The ISO value is 0.02 au. Hydrogen atoms were omitted for clarity. Positive is purple; negative is red. All energies are depicted in kilocalories per mole.

The occurrence of one-electron reduction of the diamido–dipyrrin ligand and not the uranium center in the uranyl complexes is supported by computational analysis. DFT calculations were undertaken on both UO_2_(OAc)(**L**) and UO_2_Cl(**L**) and their one-electron-reduction products. The former experiments reveal that the lowest unoccupied molecular orbitals (LUMOs) of both complexes are located entirely on the ligand, whereas in contrast, the LUMOs+1 are metal-based, indicating that one-electron reductions should indeed lead to ligand-based radicals (Figure 6). Furthermore, the LUMOs+1 suggest that the second reduction should lead to uranium reduction, i.e., to the formation of uranyl(V) complexes. The singly occupied molecular orbitals (SOMOs) of [UO_2_(OAc)(**L**^•^)]^−^ and [UO_2_Cl(**L**^•^)]^−^ are also ligand-based, and the unpaired spin-density maps of both show that the electron density is located entirely on the *meso*-carbon of the ligand, furthermore confirming the radical character of the ligand after one-electron reduction.

As shown previously, the CV of UO_2_Cl(**L**) exhibits another similar set of reductions, and it was concluded that this was due to the lability of the chloride, forming the ion pair [UO_2_(MeCN)(**L**)][Cl] in solution. A study conducted previously in the group, however, demonstrated that the cation of **2**, [UO_2_(**L**^2^)][BAr^F^], first undergoes uranyl(VI)/uranyl(V) reduction rather than the formation of a ligand radical (**L**^2^ = dipyrrin–diimine ligand).^15^ Therefore, to ensure that UO_2_Cl(**L**) and [UO_2_(MeCN)(**L**)][Cl] exhibit similar reactivity, the LUMOs of both UO_2_Cl(**L**) and [UO_2_(**L**)]^+^ were compared with those of **2** and [UO_2_(**L**^2^)]^+^ (see the SI). These calculations show that the LUMOs of both UO_2_Cl(**L**) and UO_2_Cl(**L**^2^) are ligand–based. While the LUMO of [UO_2_(**L**^2^)]^+^ exhibits both ligand and metal character and results experimentally in uranyl(VI)/uranyl(V) reduction, the LUMO of [UO_2_(**L**)]^+^ is fully ligand-based. This supports the theory that the second species seen in the CV is likely the ion pair [UO_2_(MeCN)(**L**)][Cl] and that this compound exhibits similar reactivity to the parent UO_2_Cl(**L**).

## Conclusions

The diamido–dipyrrin ligand acts as a tetradentate chelate for the uranyl dication and, because of its low-lying π* molecular orbitals, is a redox-noninnocent partner in the reduction chemistry of its uranyl complexes. The uranyl complexes UO_2_(OAc)(**L**) and UO_2_Cl(**L**) are both insensitive toward hydrolysis and could therefore be easily prepared and stored on the bench. In addition, both complexes undergo one-electron reduction when reacted with CoCp_2_, leading to ligand radicals rather than uranyl(V) complexes. Although attempts to crystallize the singly reduced complexes were unsuccessful, EPR, cyclic voltammetry, and DFT studies support the presence of a ligand radical. Our current investigations are focused on manipulation of the redox behavior of similar dipyrrin ligands in order to form air-stable uranyl(V) dipyrrin complexes.

## Experimental Section

### General Procedure

#### **Caution!** Depleted uranium (primary isotope ^238^U) is a weak α-emitter (4.197 MeV) with a half-life of 4.47 × 109 years. Manipulations and reactions should be carried out in monitored fume hoods or in an inert-atmosphere glovebox in a radiation laboratory equipped with α- and β-counting equipment.

 The syntheses of all air- and moisture-sensitive compounds were carried out using standard Schlenk techniques under an atmosphere of dry argon. Vacuum Atmospheres and MBraun gloveboxes were used to manipulate and store air- and moisture-sensitive compounds under an atmosphere of dried and deoxygenated dinitrogen. The solvents pyridine-d_5_ and THF-*d*_8_ were refluxed over potassium metal overnight, trap-to-trap-distilled, and three times free-pump-thaw-degassed prior to use. All glassware was dried in an oven at 160 °C, cooled under 10–3 mbar vacuum, and then purged with argon. Prior to use, all Fisherbrand R 1.2 mm retention glass microfiber filters and stainless-steel cannula were dried in an oven at 160 °C overnight. All solvents for use with air- and moisture-sensitive compounds were stored in Teflon-tapped ampules containing predried 4 Å molecular sieves. Solvents were collected from a solvent purification system (Innovation Technologies), where they had been passed over a column of molecular sieves for 24 h prior to collection. They were then degassed prior to use and subsequent storage. All chemicals were used as used as received without any purification, unless otherwise specified. Tetrabutylammonium hexafluorophosphate, [^n^Bu_4_N][PF_6_], was recrystallized twice from absolute EtOH and further dried for 2 days under vacuum.

^1^H NMR spectra were recorded on a Bruker AVA400 spectrometer operating at 399.90 MHz, a Bruker AVA500 or a Bruker PRO500 spectrometer operating at 500.12 MHz, or a Bruker AVA600 spectrometer operating at 599.81 MHz. ^13^C{^1^H} NMR spectra were recorded on a Bruker AVA500 or a Bruker PRO500 spectrometer operating at 125.76 MHz. ^19^F{^1^H} NMR spectra were recorded on a Bruker AVA500 spectrometer operating at 470.59 MHz. Chemical shifts are reported in parts per million. ^1^H and ^13^C{^1^H} NMR spectra are referenced to residual solvent resonances calibrated against an external standard, SiMe_4_ (*d* = 0 ppm). ^19^F{^1^H} NMR spectra are referenced to an external standard, CCl_3_F (*d* = 0 ppm). All spectra were recorded at 298 K unless otherwise specified. All data were processed using *MestReNova 12.0.3*. Full assignments are given in the Supporting Information.

Single-crystal X-ray diffraction data were collected at 120 K on an Oxford Diffraction Excalibur diffractometer using graphite-monochromated Mo Kα radiation equipped with an Eos CCD detector (λ = 0.71073 Å) or at 120 K on a Supernova Dual Cu at Zero Atlas diffractometer using Cu Kα radiation (λ = 1.5418 Å). Structures were solved using *ShelXT* direct methods or intrinsic phasing and refined using a full-matrix least-squares refinement on |*F*|^2^ using *ShelXL*.^27−29^ All programs were used within the *OLEX* suite.^30^ All non-hydrogen atoms refined with anisotropic displacement and *H* parameters were constrained to parent atoms and refined using a riding model unless otherwise specified. All single-crystal X-ray structures were analyzed and illustrated using *Mercury 4.3.1*.

Elemental analyses were carried out by Elemental Microanalysis Ltd., measured in duplicate. All Fourier transform infrared (FTIR) spectra were recorded using a JASCO 410 or a JASCO 460 plus spectrometer. Intensities are assigned as w = weak, m = medium, and s = strong. All UV–vis absorption spectra were recorded on a Jasco V-670 spectrometer on a 10 mm quartz cuvette, fitted with a septum for air-sensitive compounds.

### Synthesis

#### **4**

(Trichloroacetyl)pyrrole (4.8 g, 23 mmol, 1.0 equiv) was added to 50 mL of freshly distilled *tert*-butylamine, and the mixture was heated to 50 °C for 48 h. The solvent was removed under reduced pressure. The solid was washed with *n*-hexane (3 × 100 mL), and the remaining white solid was recrystallized from a hot ethanol (EtOH) solution. Yield: 1.42 g (39%). ^1^H NMR (400 MHz, MeOH-*d*_4_): δ_H_ 6.86 (1H, dd, *J* = 2.6 and 1.4 Hz), 6.75 (1H, dd, *J* = 3.7 and 1.4 Hz), 6.12 (1H, dd, *J* = 3.7 and 2.6 Hz), 1.43 (9H, s). ^13^C{^1^H} NMR (101 MHz, MeOH-*d*_4_): δ_C_ 158.51, 126.50, 120.95, 110.34, 108.56, 50.80, 27.90. HRMS (ESI^+^, MeOH). Calcd for C_9_H_15_N_2_O ([M + H]^+^): *m*/*z* 167.117890. Found: *m*/*z* 167.11770 (mass error = −0.19 ppm). Elem anal. Calcd for C_9_H_14_N_2_O (MW = 166.2 g mol^–1^): C, 65.03; H, 8.49; N, 16.85. Found: C, 64.91; H, 8.62; N, 16.92. FTIR (film): ν_MAX_ 1581 cm^–1^ (C5=ONH).

#### **5**

**4** (2.2 g, 14.7 mmol, 2.0 equiv) was added to PhCH_3_ (80 mL). (Pentafluorophenyl)benzaldehyde (1.5 g, 7.6 mmol, 1.0 equiv) and *p*-toluenesulfonic acid (*p*-TSA; 40 mg, 0.23 mmol, 0.03 equiv) were added to the gray suspension before the mixture was set to reflux. After 20 h, the reaction was cooled to RT. The solids were filtered and washed with PhCH_3_ (3 × 10 mL). The isolated white solid was recrystallized from *n*-hexane, resulting in a white powder. Yield: 1.45 g (36%). ^1^H NMR (400 MHz, DMSO-*d*_6_): δ_H_ 11.33 (2H, s), 7.21 (2H, s, 2H), 6.70 (2H, dd, *J* = 3.7 and 2.5 Hz), 5.86 (1H, s), 5.73 (2H, t, *J* = 3.1 Hz), 1.34 (s, 18H). ^13^C{^1^H} NMR (101 MHz, DMSO-*d*_6_): δ_C_ 160.81, 146.50, 141.97, 132.71, 127.36, 116.13, 110.54, 108.49, 108.03, 50.87, 32.96, 29.33. ^19^F{^1^H} NMR (376 MHz, DMSO-*d*_6_): δ_F_ −141.28 (2F, dd, *J* = 24.0, 6.9 Hz), −157.60 (1F, t, *J* = 22.7 Hz), −163.31 (2F, td, *J* = 23.7 and 7.0 Hz). HRMS (ESI^+^, MeOH). Calcd for C_25_H_28_F_5_N_4_O_2_ ([M + H]^+^): *m*/*z* 511.21269. Found: *m*/*z* 511.21180 (mass error = −0.89 ppm). Calcd for C_25_H_27_F_5_N_4_O_2_Na ([M + Na]^+^): *m*/*z* 533.19436. Found: *m*/*z* 533.19280 (mass error = −1.84 ppm). Elem anal. Calcd for C_25_H_27_F_5_N_4_O_2_ (MW = 510.2 g mol^–1^): C, 58.82; H, 5.33; N, 10.97. Found: C, 58.95; H, 5.36; N, 10.85. FTIR (film): ν_MAX_ 1580 cm^–1^ (C5=ONH).

#### H**L**

**5** (950 mg, 1.86 mmol, 1.0 equiv) was dissolved in THF (150 mL) and DDQ (460 mg, 2.02 mmol, 1.1 equiv) dissolved in THF (100 mL) was slowly added over a period of 20 min., during which the dark-greenish yellow solution slowly turned dark red. After 22 h, the mixture was concentrated, redissolved in CH_2_Cl_2_ (50 mL), and filtered. The filtrate was concentrated. The crude product was purified by silica column chromatography (1 = 100% CH_2_Cl_2_; 2 = 98:2 CH_2_Cl_2_/EtOH; rf = 0.3; bright-pink-orange fraction), resulting in a bright-greenish-orange solid. Orange single crystals suitable for X-ray crystallography were obtained through the slow evaporation of a concentrated DMSO solution. Yield: 240 mg (25%). ^1^H NMR (400 MHz, chloroform-*d*): δ_H_ 12.69 (1H, bs), 6.77 (2H, d, *J* = 4.4 Hz), 6.60 (2H, bs), 6.51 (2H, d, *J* = 4.4 Hz), 1.53 (18H, s). ^13^C{^1^H} NMR (101 MHz, chloroform-*d*): δ_C_ 159.89, 151.45, 145.88, 143.42, 141.28, 138.70, 137.77, 127.97, 125.63, 117.88, 51.79, 28.75. ^19^F{^1^H} NMR (376 MHz, chloroform-*d*): δ_F_ −132.11 to −142.09 (2F, m), −151.07 (1F, t, *J* = 21.1 Hz), −157.37 to −166.39 (2F, m). HRMS (ESI^+^, MeOH). Calcd for C_25_H_26_F_5_N_4_O_2_ ([M + H]^+^): *m*/*z* 509.19704. Found: *m*/*z* 509.19419 (mass error = −2.94 ppm). Calcd for C_25_H_25_F_5_N_4_O_2_Na ([M + Na]^+^): *m*/*z* 531.17899. Found: *m*/*z* 531.17700 (mass error = −1.99 ppm). Elem anal. Calcd for C_25_H_25_F_5_N_4_O_2_ (MW = 508.2 g mol^–1^): C, 59.05; H, 4.96; N, 11.02. Found: C, 58.93; H, 4.94; N, 10.94. FTIR (film): ν_MAX_ 1652 cm^–1^ (C5=ONH). UV–vis (CH_2_Cl_2_): λ = 252 nm, ε = 19500 M^–1^ cm^–1^; λ_max_ = 470 nm, ε = 27 280 M^–1^ cm^–1^.

#### UO_2_(OAc)(**L**)

A solution of H**L** (100 mg, 0.197 mmol, 1 equiv; in 1:3 MeOH/CHCl_3_, 70 mL) was added to a solution of UO_2_(OAc)_2_·2H_2_O (91.8 mg, 0.217 mmol, 1.1 equiv; in 1:3 MeOH/CHCl_3_, 20 mL), after which triethanolamine (NEt_3_) was added (36 μL, 0.256 mmol, 1.3 equiv), causing an immediate color change from orange to pink. The reaction mixture was heated to 65 °C and stirred for 18 h, after which the solvent was removed under reduced pressure. The oil was redissolved in CH_2_Cl_2_ (75 mL), washed with H_2_O (3 × 15 mL), and dried with MgSO_4_. A greenish-pink solid was obtained. Greenish-pink single crystals suitable for X-ray crystallography were obtained through the slow evaporation of a concentrated THF solution. Yield: 127 mg (77%). ^1^H NMR (400 MHz, MeOH-*d*_4_): δ_H_ 7.59 (2H, d, *J* = 4.5 Hz), 7.15 (2H, d, *J* = 4.5 Hz), 2.17 (3H, bs), 1.80 (18H, s). ^13^C{^1^H} NMR (126 MHz, MeOH-*d*_4_): δ_C_ 169.76, 158.86, 144.80, 143.44, 142.21, 137.67, 137.41, 133.53, 128.46, 119.00, 54.04, 27.70. ^19^F{^1^H} NMR (376 MHz, MeOH-*d*_4_): δ_F_ −141.82 (2F, dd, *J* = 21.3 and 5.9 Hz), −155.06 (1F, t, *J* = 20.6 Hz), −163.97 (2F, td, *J* = 20.7 and 6.0 Hz). HRMS (ESI^+^, MeOH). Calcd for C_27_H_28_F_5_N_4_O_6_U ([M + H]^+^): *m*/*z* 837.24314. Found: *m*/*z* 837.25460 (mass error = 11.46 ppm). Calcd for C_27_H_27_F_5_N_4_O_6_UNa ([M + Na]^+^): *m*/*z* 859.22508. Found: *m*/*z* 859.22830 (mass error = 3.22 ppm). Calcd for C_27_H_24_F_5_N_4_O_4_U ([M – OAc]^+^): *m*/*z* 777.22201. Found: *m*/*z* 777.22640 (mass error = 5.64 ppm). Elem anal. Calcd for C_27_H_27_F_5_N_4_O_6_U (MW = 836.24 g mol^–1^): C, 38.77; H, 3.25; N, 6.70. Found: C, 38.83; H, 3.35; N, 6.51. FTIR (film): ν 2962 (w), 2925 (w), 1590 (s), 1575 (s), 1520 (s), 1501 (s), 1495 (m), 1370 (m), 1352 (m), 1332 (w), 1292 (m), 1247 (s), 1199 (s), 1072 (m), 1005 (s), 979 (s), 951 (m), 905 (s), 837 (s), 805 (m), 758 (m), 743 (m), 725 (m), 713 (m), 645 (m) cm^–1^. UV–vis (THF): λ = 512 nm, ε = 20812 M^–1^ cm^–1^; λ_max_ = 546.5 nm, ε = 82316 M^–1^ cm^–1^.

#### UO_2_Cl(**L**)

Method A: A solution of H**L** (131 mg, 0.257 mmol, 1 equiv; in 1:3 MeOH/CHCl_3_, 150 mL) was added to a solution of UO_2_Cl_2_THF_2_ (137 mg, 0.283 mmol, 1.1 equiv; in 1:3 MeOH/CHCl_3_, 20 mL), after which NEt_3_ was added (47 μL, 0.334 mmol, 1.3 equiv), causing an immediate color change from orange to red. The reaction mixture was heated to 65 °C and stirred for 18 h, after which the solvent was removed under reduced pressure. The majority of the red solid was redissolved in CH_2_Cl_2_ (400 mL) and filtered. The filtrate was washed with H_2_O (3 × 50 mL), dried with MgSO_4_, and concentrated to obtain a red solid. The red solid and residue were combined, yielding a red solid. Yield: 189 mg (91%). ^1^H NMR (400 MHz, MeCN-*d*_3_): δ_H_ 8.22 (2H, s), 7.46 (2H, d, *J* = 4.4 Hz), 7.19 (2H, d, *J* = 4.4 Hz), 1.76 (18H, s). ^13^C{^1^H} NMR (126 MHz, MeCN-*d*_3_): δ_C_ 169.78, 159.02, 144.81, 143.17, 142.42, 138.05, 137.56, 134.23, 132.34, 119.57, 55.02, 27.84. ^19^F{^1^H} NMR (376 MHz, MeCN-*d*_3_): δ_F_ −140.88 to −143.36, −154.28 to −155.13, −162.18 to −163.97. HRMS (ESI^+^, MeOH). Calcd for C_25_H_25_F_5_N_4_O_4_ClU ([M + H]^+^): *m*/*z* 813.19869. Found: *m*/*z* 813.19580 (mass error = −2.89 ppm). Elem anal. Calcd for C_25_H_24_ClF_5_N_4_O_4_U (MW = 836.24 g mol^–1^): C, 36.94; H, 2.98; N, 6.89. Found: C, 36.96; H, 3.02; N, 6.48. FTIR (film): ν 3300 (w), 3270 (w), 2972 (w), 1592 (s), 1570 (s), 1521 (s), 1489 (s), 1460 (m), 1374 (m), 1370 (m), 1348 (m), 1291(m), 1264 (s), 1200 (s), 1164 (m), 1075 (s), 1053 (w), 1007 (s), 974 (s), 978 (s), 950 (m), 912 (s), 839 (s), 804 (m), 771 (m), 743 (s), 726 (m), 714 (m), 647 (m) cm^–1^. UV–vis (THF): λ = 514 nm, ε = 18 582 M^–1^ cm^–1^; λ_max_ = 546 nm, ε = 84 301 M^–1^ cm^–1^.

Method B: A solution of H**L** (35 mg, 0.068 mmol, 1.0 equiv) in dry THF (3 mL) was dropwise added to a slurry of KH (3 mg, 0.0746, 1.1 equiv) in dry THF (2 mL). The solution slowly turned pinkish red and was left to stir overnight, after which it was dropwise added to a yellow slurry of UO_2_Cl_2_THF_2_ (32 mg, 0.068 mmol, 1.0 equiv) in dry THF (2 mL), causing an immediate color change from red to pink. The reaction mixture was stirred at RT for 18 h, after which the reaction mixture was transferred to the bench and the solvent was removed under reduced pressure. The solid was partially dissolved in CH_2_Cl_2_ (30 mL). The filtrate was washed with H_2_O (3 × 3 mL), dried over MgSO_4_, and concentrated. Both the residue and washed filtrate were combined, obtaining a red solid.

#### [Cp_2_Co][UO_2_(OAc)(**L**^•^)]

A pink solution of UO_2_(OAc)(**L**) (50 mg, 0.06 mmol, 1.0 equiv) in dry THF (5 mL) was added to a solution of CoCp_2_ (11.3 mg, 0.06 mmol, 1.0 equiv) in dry THF (1 mL). The solution turned dark greenish red instantaneously, and a green precipitate started to form. The reaction was left to stir for 1 h before it was centrifuged. Greenish-brown solids were obtained. Yield: 46 mg (75%). NMR silence. EPR: *S* = ^1^/_2_ and *g*_iso_ = 1.997. Elem anal. Calcd for C_37_H_37_CoF_5_N_4_O_6_U (MW = 1025.25 g mol^–1^): C, 43.33; H, 3.63; N, 5.46. Found: C, 43.44; H, 3.50; N, 5.44. UV–vis (pyridine): λ = 551 nm, ε = 10500 M^–1^ cm^–1^; λ_max_ = 500 nm, ε = 45700 M^–1^ cm^–1^.

#### [Cp_2_Co][UO_2_Cl(**L**^•^)]

A pink solution of UO_2_(Cl)(**L**) (49 mg, 0.06 mmol, 1.0 equiv) in dry THF (5 mL) was added to a solution of CoCp_2_ (11.3 mg, 0.06 mmol, 1.0 equiv) in dry THF (1 mL). The solution turned dark greenish red instantaneously, and a green precipitate started to form. The reaction was left to stir for 1 h before it was centrifuged. Greenish-brown solids were obtained. Yield: 55 mg (91%). NMR silence. EPR: *S* = ^1^/_2_ and *g*_iso_ = 1.997. Elem anal. Calcd for C_35_H_34_ClCoF_5_N_4_O_4_U (MW = 1002.09 g mol^–1^): C, 41.95; H, 3.42; N, 5.59. Found: C, 41.34; H, 3.31; N, 5.30. UV–vis (pyridine): λ = 551 nm, ε = 10500 M^–1^ cm^–1^; λ_max_ = 500 nm, ε = 45700 M^–1^ cm^–1^.

## Supporting Information Available

Full synthetic procedures, X-ray crystallography, DFT calculations, EPR spectroscopy, and electrochemical methods (PDF)

## Accession Codes

CCDC 2122704–2122706 contain the supplementary crystallographic data for this paper. These data can be obtained free of charge via www.ccdc.cam.ac.uk/data_request/cif, or by emailing data_request@ccdc.cam.ac.uk, or by contacting The Cambridge Crystallographic Data Centre, 12 Union Road, Cambridge CB2 1EZ, UK; fax: +44 1223 336033.
