# Supplementary material for: Exploring the Redox Properties of Bench-Stable Uranyl(VI) Diamido–Dipyrrin Complexes
Source: Inorg Chem. 2022 Feb 7;61(7):3249–55. doi: 10.1021/acs.inorgchem.1c03744 (PMC9007458; doi:10.1021/acs.inorgchem.1c03744)
Supplement: Supplementary file 1 — ic1c03744_si_001.pdf [file ic1c03744_si_001.pdf]

SI: Exploring the redox properties of bench-stable uranyl(VI) diamidodipyrrin complexes

Karlotta van Rees[a], Emma K. Hield[a], Ambre Carpentier[b], Laurent Maron[b], Stephen Sproules[c], and Jason B. Love*[a]

[a]EaStCHEM School of Chemistry, University of Edinburgh, Joseph Black Building, David Brewster Road, Edinburgh, EH9 3FJ. [b]LPCNO, INSA, Université de Toulouse, 135, avenue de Rangueil, 31077, Toulouse cedex 4, France. [c]WestCHEM School of Chemistry, University of Glasgow, Glasgow, G12 8QQ, UK.

Contents

1 General procedure 2
2 Synthetic procedures 4
2.1 Synthesis of N-tert-butanyl-1H-pyrrole-2-carboxamide 4
2.2 1,9-Di-*tert*-butylamide-5(pentafluorphenyl)dipyrromethane 6
2.3 1,9-Di-*tert*-butylamide-5(pentafluorphenyl)dipyrrin H**L** 9
2.4 Uranyl acetate complex UO_2(OAc)(**L**) 13
2.5 Uranyl chloride complex UO_2Cl(**L**) 17
2.6 [Cp_2Co][UO_2(OAc)(**L•**)] 21
2.7 [Cp_2Co][UO_2Cl(**L•**)] 22
3 Crystallography 23
4 Optimised DFT geometries 26
4.1 UO_2Cl(**L**) 26
4.2 [UO_2(**L**)]$^+$ 26
4.3 UO_2(OAc)(**L**) 27
4.4 UO_2(OAc)(**L°**) 28
U 5.289433 2.292172 14.871584 28
4.5 UO_2(OAc)(**L°**) 29
5 Calculated Molecular orbitals 30
5.1 Compound $U^{VI}O_2$(OAc)(**L**) 30
5.2 Compound $U^{VI}O_2$(OAc)(**L•**) 32
5.3 Compound $U^{VI}O_2$Cl(**L**) 34
5.4 Compound $U^{VI}O_2$(Cl)(**L•**) 36
5.5 Cationic species 38
6 Electrochemistry 39
7 EPR 49

8 References 50

1 General procedure

The syntheses of all air- and moisture-sensitive compounds were carried out using standard Schlenk techniques under an atmosphere of dry argon. Vacuum Atmospheres and MBraun glove boxes were used to manipulate and store air- and moisture-sensitive compounds under an atmosphere of dried and deoxygenated dinitrogen. The solvents pyridine-d_5 and THF-d_8 were refluxed over potassium metal overnight, trap-to-trap distilled and three times free-pump-thaw degassed prior to use. All glassware was dried in an oven at 160 °C, cooled under 10^{-3} mbar vacuum and then purged with argon. Prior to use, all Fisherbrand R 1.2 *m*m retention glass microfiber filters and stainless-steel cannulae were dried in an oven at 160 °C overnight. All solvents for use with air- and moisture-sensitive compounds were stored in Teflon-tapped ampoules containing pre-dried 4 Å molecular sieves. Solvents were collected from a solvent purification system (Innovation Technologies), where they had been passed over a column of molecular sieves for 24 hours prior to collection. They were then degassed prior to use and subsequent storage. All chemicals were used as used as received without any purification, unless otherwise specified. Tetrabutylammonium hexafluorophosphate, [^{n}Bu$_4$N][PF$_6$], was recrystallized twice from absolute ethanol and further dried for two days under vacuum.

^{1}H NMR spectra were recorded on a Bruker AVA400 spectrometer operating at 399.90 MHz, a Bruker AVA500 or Bruker PRO500 operating at 500.12 MHz or a Bruker AVA600 spectrometer operating at 599.81 MHz. ^{13}C{^{1}H} NMR spectra were recorded on a Bruker AVA500 or Bruker PRO500 operating at 125.76 MHz. ^{19}F{^{1}H} NMR spectra were recorded on a Bruker AVA500 spectrometer operating at 470.59 MHz. Chemical shifts are reported in parts per million (ppm). ^{1}H and ^{13}C{^{1}H} NMR spectra are referenced to residual solvent resonances calibrated against an external standard, SiMe$_4$ (d = 0 ppm). ^{19}F{^{1}H} NMR spectra are referenced to an external standard, CCl$_3$F (d = 0 ppm). All spectra were recorded at 298 K unless otherwise specified. All data were processed using MestReNova 12.0.3.

Single crystal X-ray diffraction data were collected at 170 K on an Oxford Diffraction Excalibur diffractometer using graphite monochromated Mo-Kα radiation equipped with an Eos CCD detector (λ = 0.71073 Å), or at 120 K on a Supernova, Dual, Cu at Zero Atlas diffractometer using Cu-Kα radiation (λ = 1.5418 Å). Structures were solved using ShelXT direct methods or intrinsic phasing and refined using a full-matrix least square refinement on $|F|^2$ using ShelXL.[1-3] All programs were used within the Olex suite.[4] All non-hydrogen atoms refined with anisotropic displacement parameters and H-parameters were constrained to parent atoms and refined using a riding model unless otherwise specified. All X-ray crystal structures were analysed and illustrated using Mercury 4.1.0.

For the uranium complexes. The optimization of different spin states for uranium complexes was carried out by employing DFT hybrid functional (B3PW91)[5, 6] along with small core pseudopotential Stuggart basis set for uranium, chlorine with additional polarization functions for chlorine atom.[7, 8] Pople basis sets (6-31G** for carbon, nitrogen, oxygen, hydrogen atoms) were employed for the rest of the atoms.[9, 10] Frequency calculations were performed to locate minima for the optimized structures. Disperison corrections were included in our calculations by employing D3 version of Grimme's dispersion with Becke-Johnson damping.[11] All the calculations were performed using Gaussian 09 suite of programs.[12]

Elemental analyses were carried out by Elemental Microanalysis Ltd., measured in duplicate. All FT-IR spectra were recorded using JASCO 410 or JASCO 460 plus spectrometers. Intensities are assigned as: w = weak, m = medium, and s = strong. All UV-vis absorption spectra were recorded on a Jasco V-670 spectrometer on a 10 mm quartz cuvette, fitted with a septum for air-sensitive compounds.

Electrochemical measurements were made using an Autolab 302 potentiostat and the data processed using NOVA 2.1.4. Experiments were undertaken at room temperature in a N_2 glovebox in a 4-neck rbf with a glassy-carbon disk working electrode, a platinum gauze as the counter electrode and a silver wire pseudo-reference electrode. The solution employed was 1.0 mM of the compound with 0.1 N [$^{n}Bu_4N$][PF_6] as the supporting electrolyte, in 20 mL dry and deoxygenated MeCN. All potentials were references against $[FeCp_2]^{+/0}$ ($E_{1/2}$, $[FeCp_2]^{+/0}$ = 0.0 V). Cyclic voltammograms were measured for quiescent solutions at variable scan rates. Square-wave voltammograms were also measured for quiescent solution, using frequency of 25 Hz, amplitude of 20 mV and step-potential of 5 mV, giving a scan-rate of 125 mV s^{-1}.

X-band EPR spectra were measured in CH_2Cl_2 solutions on a Bruker ELEXSYS E500 spectrometer and simulations performed using Bruker's Xsophe software package.[13]

Caution: *Depleted uranium (primary isotope 238U) is a weak α-emitter (4.197 MeV) with a half-life of 4.47×109 years. Manipulations and reactions should be carried out in monitored fume hoods or in an inert atmosphere glovebox in a radiation laboratory equipped with α- and β-counting equipment.*

2 Synthetic procedures

2.1 Synthesis of N-tert-butanyl-1H-pyrrole-2-carboxamide

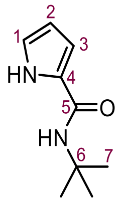

4

Trichloroacetylpyrrole (4.8 g, 23 mmol, 1.0 eq.) was added to 50 mL of freshly distilled *tert*-butylamine, and the mixture was heated to 50 °C for 48 h. Solvent was removed under reduced pressure. Solid was washed with *n*-hexane (3 x 100 mL) and the remaining white solid was recrystallised from a hot EtOH solution. Yield = 1.42 g (39 %) ^{1}H NMR (400 MHz, Methanol-d_4): δ_H 6.86 (1H, dd, *J* = 2.6 1.4 Hz, C(1)*H*), 6.75 (1H, dd, *J* = 3.7, 1.4 Hz, C(2)*H*), 6.12 (1H, dd, *J* = 3.7, 2.6 Hz, C(3)*H*), 1.43 (9H, s, C(7)H_3). ^{13}C{^{1}H} NMR (101 MHz, Methanol-d_4): δ_C 158.51 (*C*(5)), 126.50 (*C*(4)), 120.95 (*C*(1)H), 110.34 (*C*(2)H), 108.56 (*C*(3)H), 50.80 (*C*(6)), 27.90 (*C*(7)H_3). HRMS (ESI$^+$, MeOH): *m/z* $C_9H_{15}N_2O$ [M+H]$^+$ requires 167.117890, found 167.11770 (mass error = –0.19 ppm). EA: $C_9H_{14}N_2O$ (MW = 166.2 gmol^{-1}) requires C,65.03 %; H,8.49 %; N,16.85 %. Found: C,64.91 %; H,8.62 %; N,16.92 %. FTIR (film) ν_{MAX} 1581 cm^{-1} C(5)=ONH.

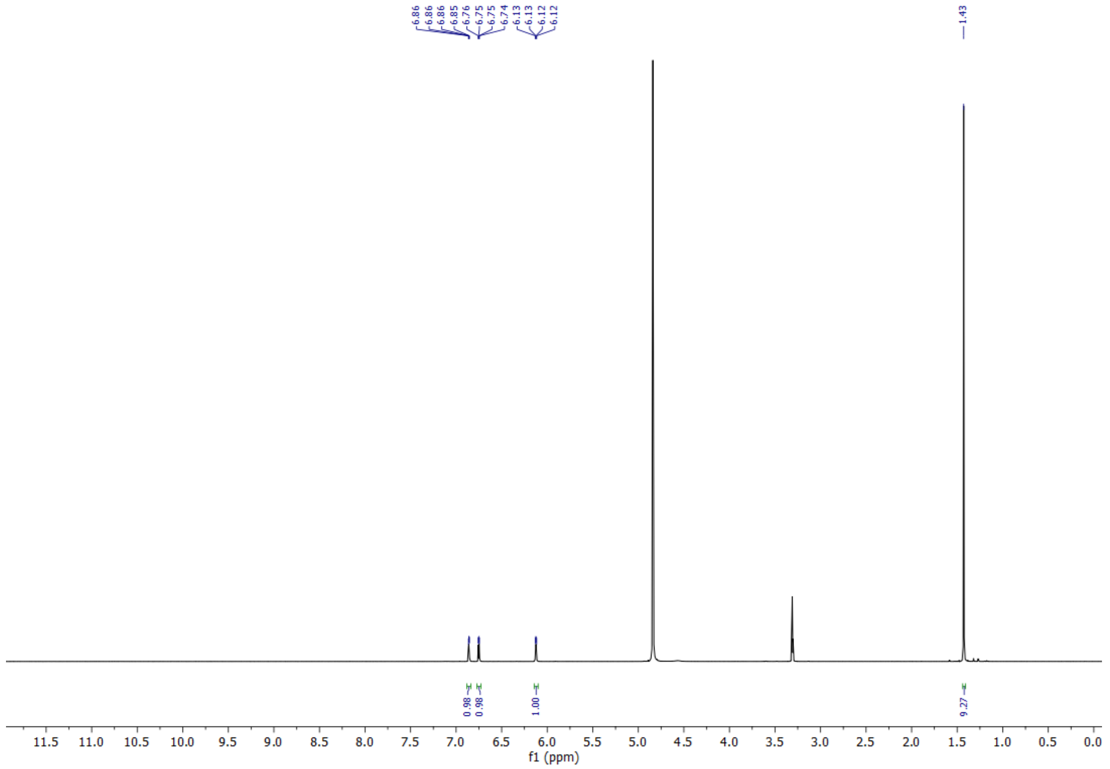

Figure S1 ^{1}H NMR of **4** in methanol-d_4.

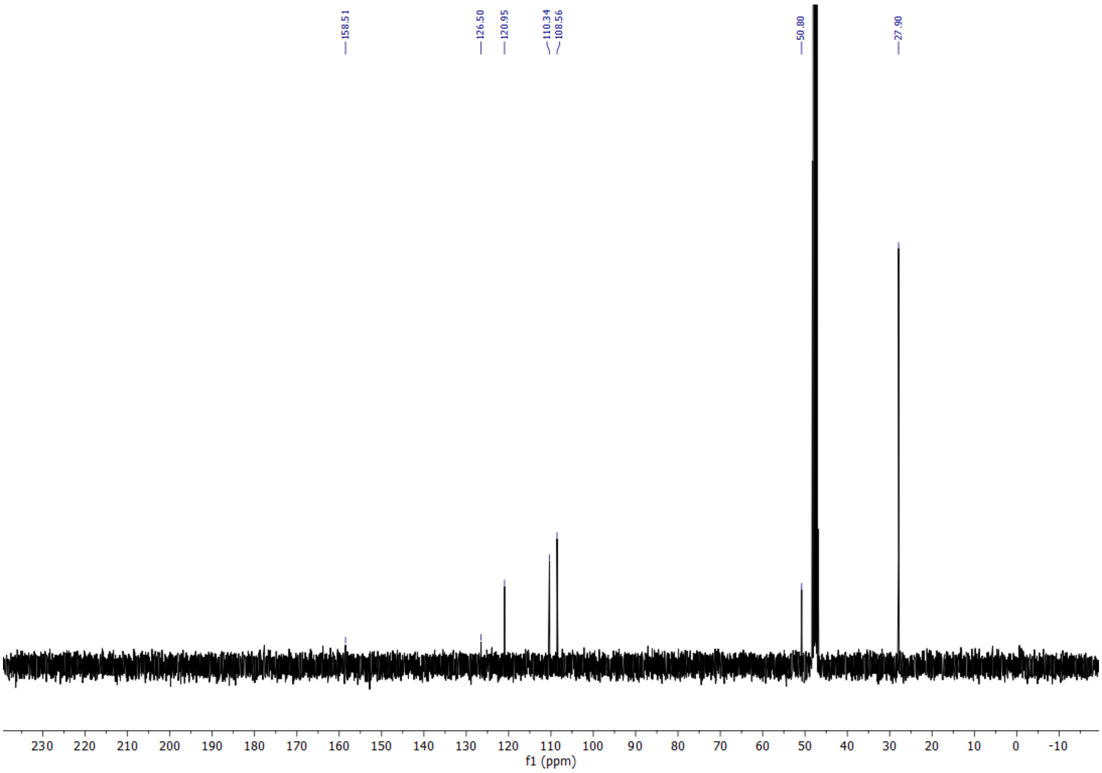

Figure S2 $^{13}C\{^{1}H\}$ NMR of **4** in methanol-d_4.

2.2 1,9-Di-*tert*-butylamide-5(pentafluorphenyl)dipyrromethane

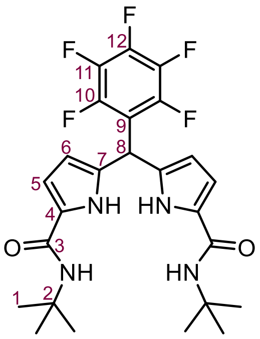

5

4 (2.2 g, 14.7 mmol, 2.0 eq.) was dissolved in $PhCH_3$ (80 mL). Pentafluorphenylbenzaldehyde (1.5 g, 7.6 mmol, 1.0 eq.) and *p*-TSA (40 mg, 0.23 mmol, 0.03 eq.) were added to the grey suspension before the mixture was set to reflux. After 20 h the reaction was cooled back to RT. The solids were filtered and washed with $PhCH_3$ (3 x 10 mL). The isolated white solid was recrystallized from *n*-hexane, resulting in a white powder. Yield = 1.45 g (36 %) ^{1}H NMR (400 MHz, Dimethylsulfoxide-d_6): δ_H 11.33 (2H, s, pyrrole-N*H*), 7.21 (2H, s, 2H, C(3)ON*H*), 6.70 (2H, dd, *J* = 3.7, 2.5 Hz, C(5)*H*), 5.86 (1H, s, C(8)*H*), 5.73 (2H, t, *J* = 3.1 Hz, C(6)*H*), 1.34 (s, 18H, C(1)H_3). $^{13}C\{^{1}H\}$ NMR (101 MHz, Dimethylsulfoxide-d_6): δ_C 160.81 (*C*(3)), 146.50 (*C*(12)F), 141.97 (*C*(11)F), 132.71 (*C*(7)), 127.36 (*C*(4)), 116.13 (*C*(10)), 110.54 (*C*(5)H), 108.49 (*C*(9)F), 108.03 (*C*(6)H), 50.87 (*C*(2)), 32.96 (*C*(8)), 29.33 (*C*(1)H_3). $^{19}F\{^{1}H\}$ NMR (376 MHz, Dimethylsulfoxide-d_6): δ_F -141.28 (2F, dd, *J* = 24.0, 6.9 Hz, Ar^F *ortho*-F), -157.60 (1F, t, *J* = 22.7 Hz, Ar^F *para*-F), -163.31 (2F, td, J = 23.7, 7.0 Hz, Ar^F *meta*-F). HRMS (ESI^+, MeOH): *m/z* $C_{25}H_{28}F_5N_4O_2$ $[M+H]^+$ requires 511.21269, found 511.21180 (mass error = -0.89 ppm), $C_{25}H_{27}F_5N_4O_2Na$ $[M+Na]^+$ requires 533.19436, found 533.19280 (mass error = -1.84 ppm). EA: $C_{25}H_{27}F_5N_4O_2$ (MW = 510.2 $gmol^{-1}$) requires C,58.82 %; H,5.33 %; N,10.97 %. Found C,58.95 %; H,5.36 %; N,10.85 %. FTIR (film) ν_{MAX} 1580 cm^{-1} C(5)=ONH.

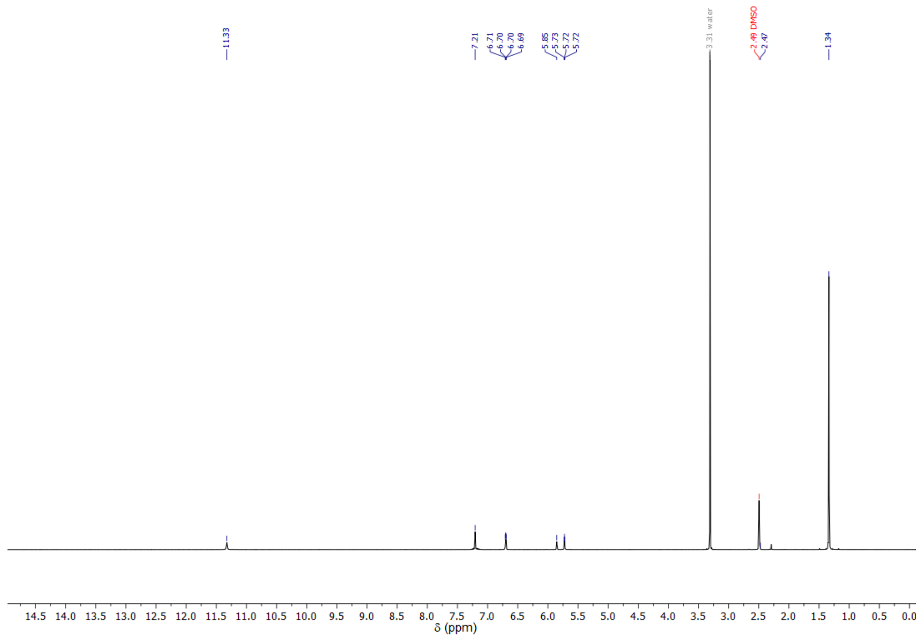

Figure S3 ^{1}H NMR of **5** in dimethylsulfoxide-d_6.

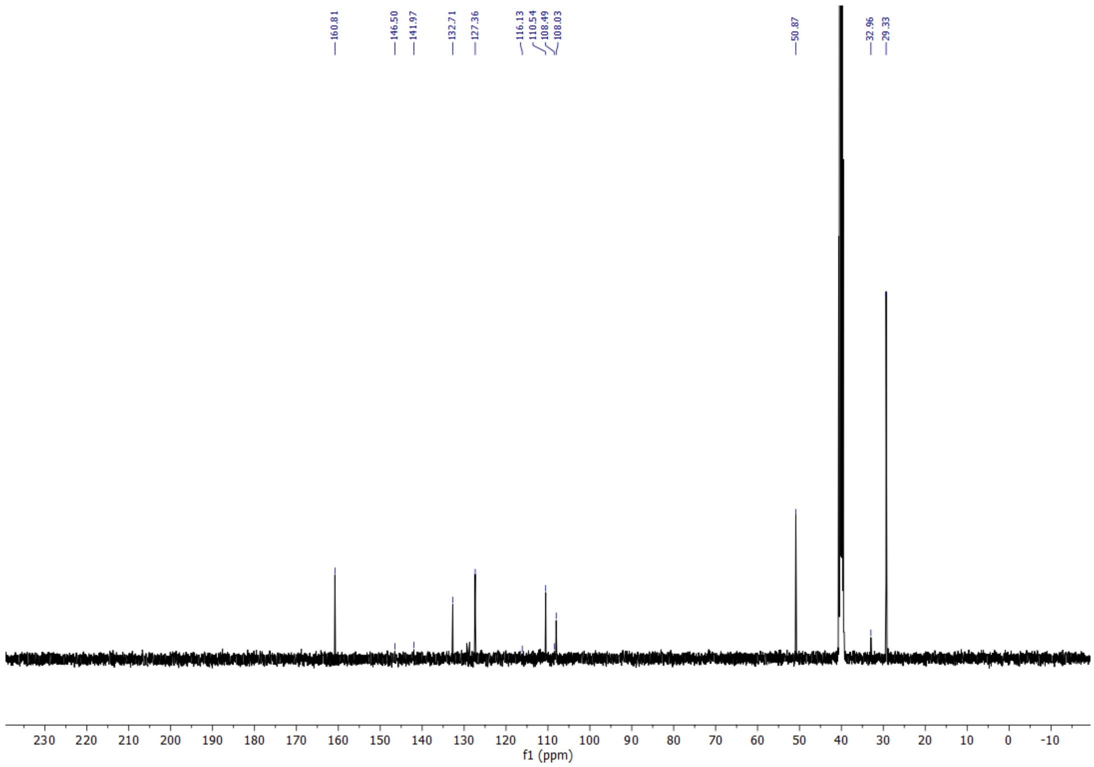

Figure S4 $^{13}C\{^{1}H\}$ NMR of **5** in dimethylsulfoxide-d_6.

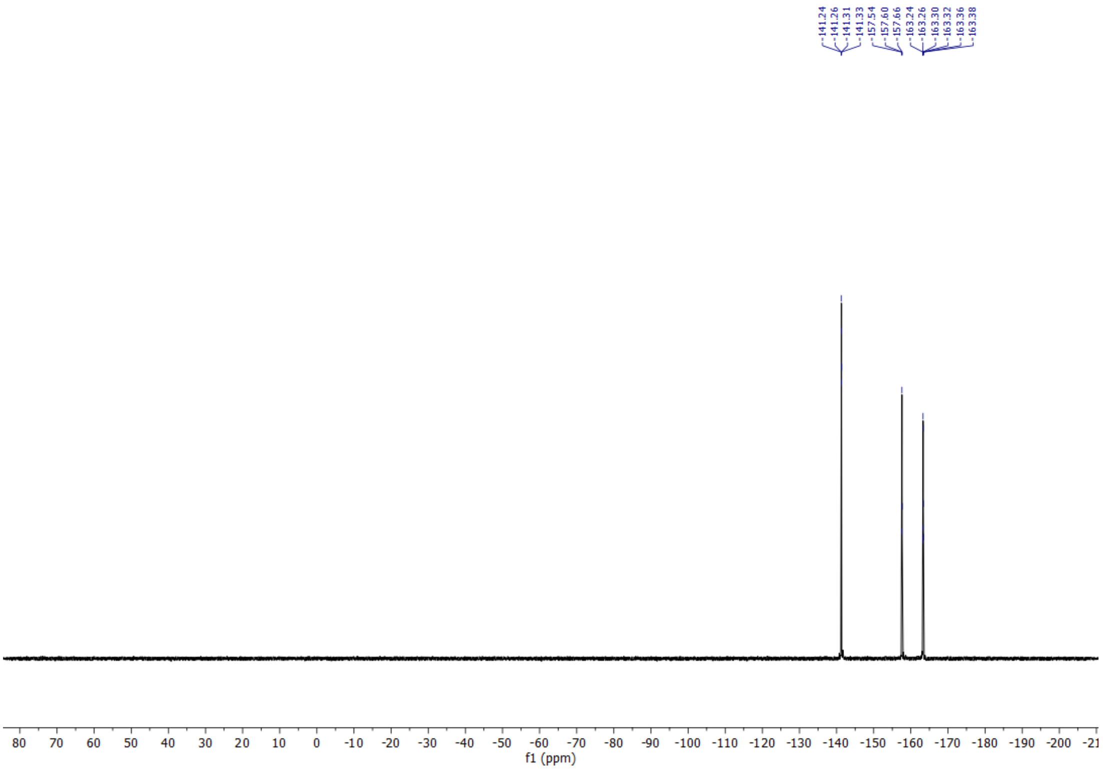

Figure S5 $^{19}F\{^{1}H\}$ NMR of **5** in dimethylsulfoxide-d_6.

2.3 1,9-Di-*tert*-butylamide-5(pentafluorphenyl)dipyrrin H**L**

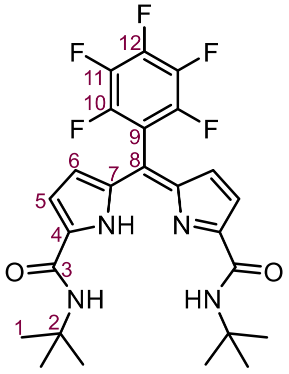

H**L**

2 (950 mg, 1.86 mmol, 1.0 eq.) was dissolved in THF (150 mL). A solution of DDQ (460 mg, 2.02 mmol, 1.1 eq.) in THF (100 mL) was slowly added over a period of 20 minutes. The then dark-greenish yellow solution slowly turned dark red. After 22 h, the mixture was concentrated, redissolved in CH_2Cl_2 (50 mL) and filtered. The filtrate was concentrated. The crude product was purified by silica column chromatography (1 = 100 % CH_2Cl_2; 2 = 98:2 CH_2Cl_2:EtOH; rf =0.3; bright pinking orange fraction) resulting in a bright greenish orange solid. Orange single crystals suitable for X-ray crystallography were obtained through slow evaporation of a concentration DMSO solution. Yield = 240 mg (25 %). ^{1}H NMR (400 MHz, Chloroform-*d*) δ_H 12.69 (1H, bs, pyrrole-N*H*), 6.77 (2H, d, *J* = 4.4 Hz, C(5)*H*), 6.60 (2H, bs, C(3)ON*H*), 6.51 (2H, d, *J* = 4.4 Hz, C(6)*H*), 1.53 (18H, s, C(1)H_3). ^{13}C{^{1}H} NMR (101 MHz, Chloroform-*d*) δ_C 159.89 (*C*(3)), 151.45 (*C*(7)), 145.88 (*C*(12)F), 143.42 (*C*(11)F), 141.28 (*C*(4)), 138.70 (*C*(10)F), 137.77 (*C*(9)), 127.97 (*C*(6)H), 125.63 (*C*(8)), 117.88 (*C*(5)H), 51.79 (*C*(2)), 28.75 (*C*(1)H_3). ^{19}F{^{1}H} NMR (376 MHz, Chloroform-*d*): δ_F -132.11 – -142.09 (2F, m, Ar^F *ortho*-F), -151.07 (1F, t, *J* = 21.1 Hz, Ar^F *para*-F), -157.37 – -166.39 (2F, m, Ar^F *meta*-F). MS (ESI$^+$, MeOH): *m/z* $C_{25}H_{26}F_5N_4O_2$ [M+H]$^+$ requires 509.19704, found 509.19419 (mass error = -2.94 ppm), $C_{25}H_{25}F_5N_4O_2Na$ [M+Na]$^+$ requires 531.17899, found 531.17700 (mass error = -1.99 ppm). EA for $C_{25}H_{25}F_5N_4O_2$ (MW = 508.2 gmol^{-1}) requires C,59.05 %; H,4.96 %; N,11.02 %. Found C,58.93 %; H,4.94 %; N,10.94 %. FTIR (film) ν_{MAX} 1652 cm^{-1} C(5)=ONH. UV-vis (CH_2Cl_2): λ 252 nm, ε = 19,500 M^{-1} cm^{-1}; λ_{max} 470 nm, ε = 27,280 M^{-1} cm^{-1}.

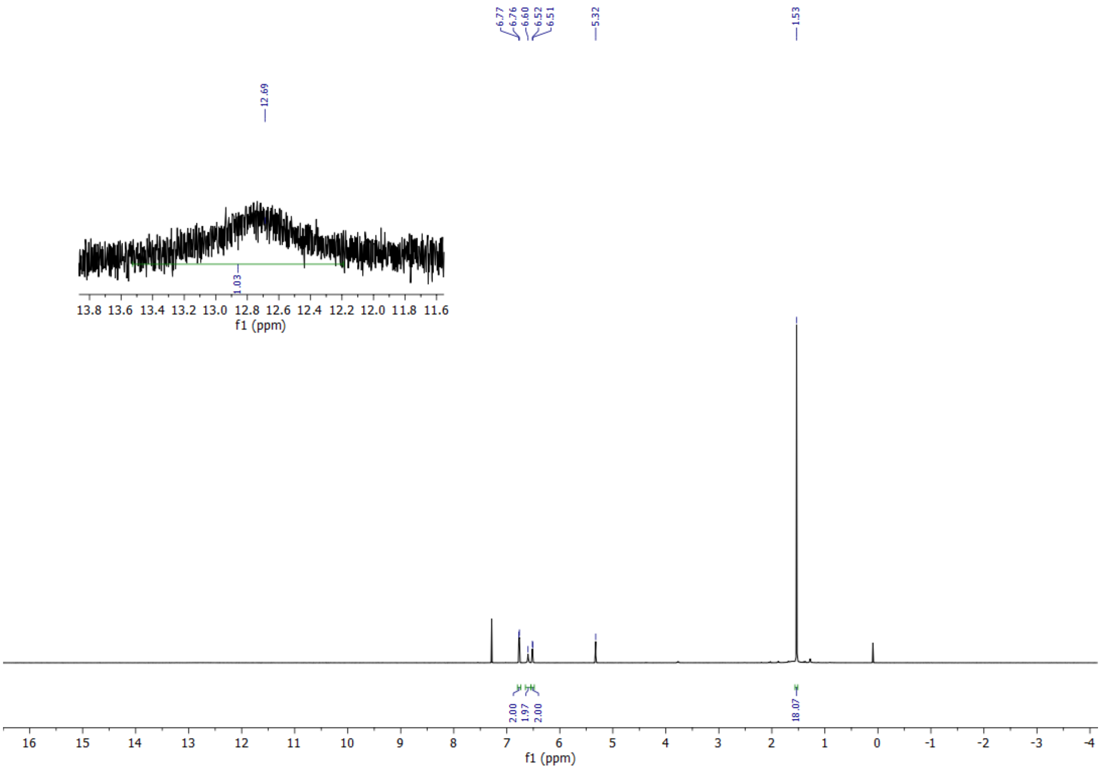

Figure S6 ^{1}H NMR of H**L** in chloroform-*d*.

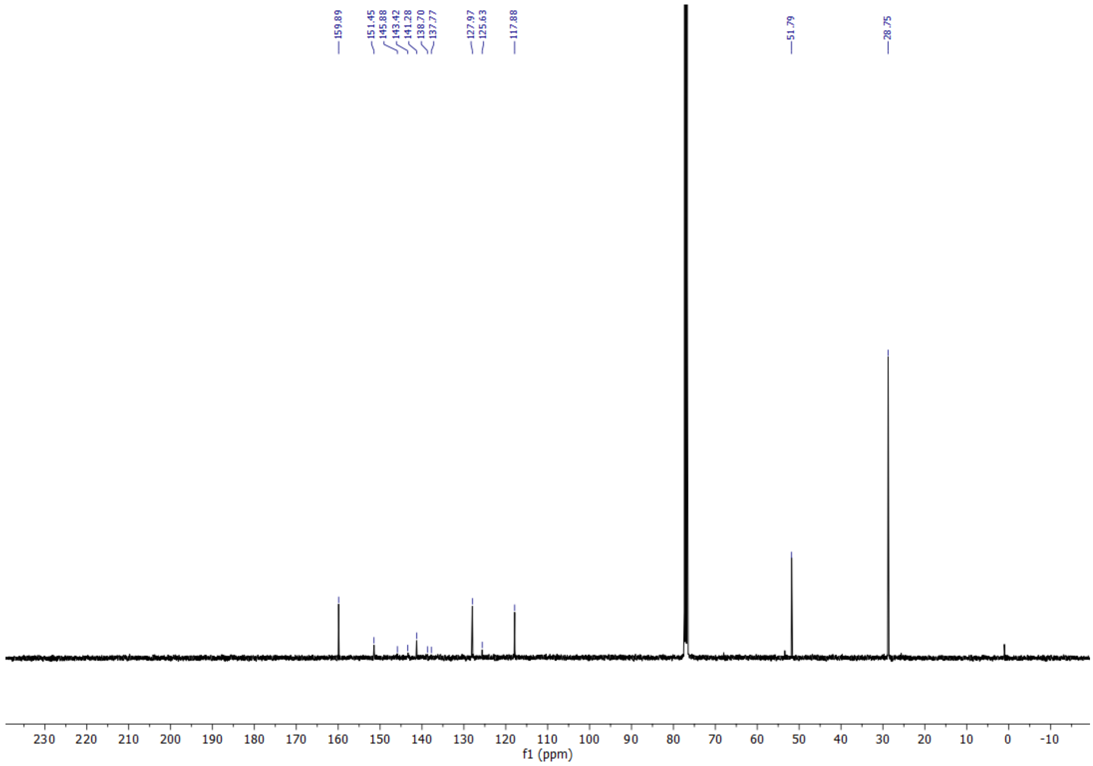

Figure S7 $^{13}C\{^{1}H\}$ NMR of H**L** in chloroform-*d*.

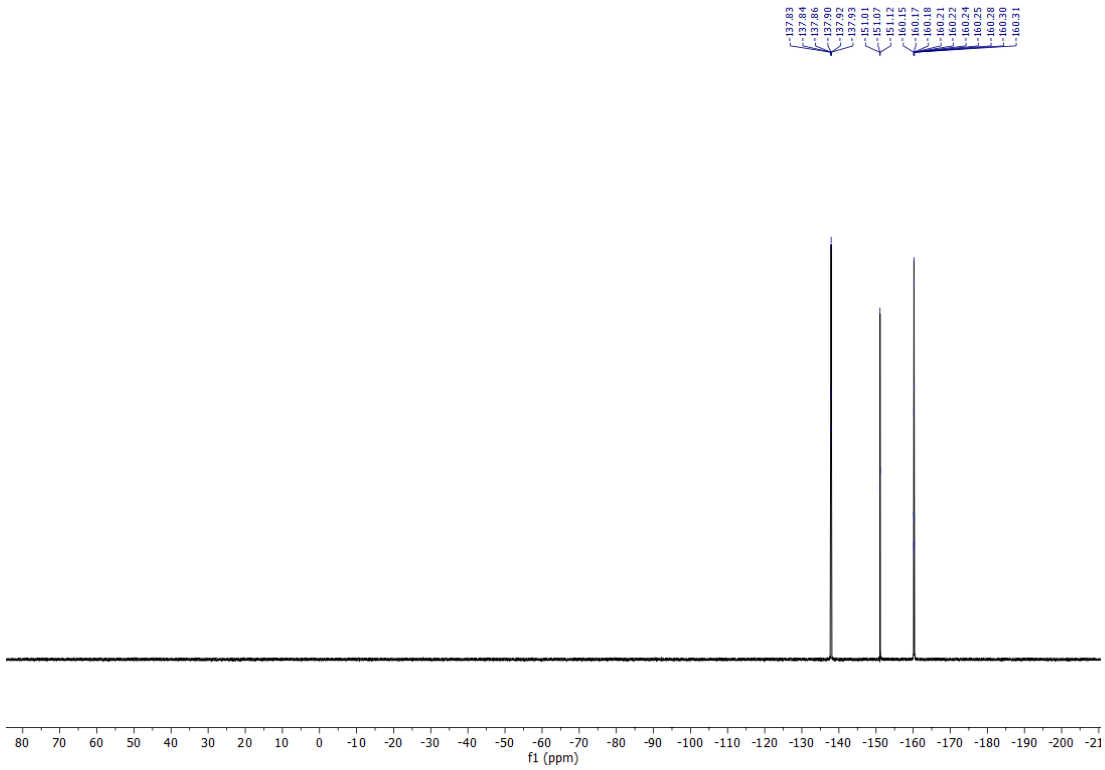

Figure S8 $^{19}F\{^{1}H\}$ NMR of H**L** in chloroform-*d*.

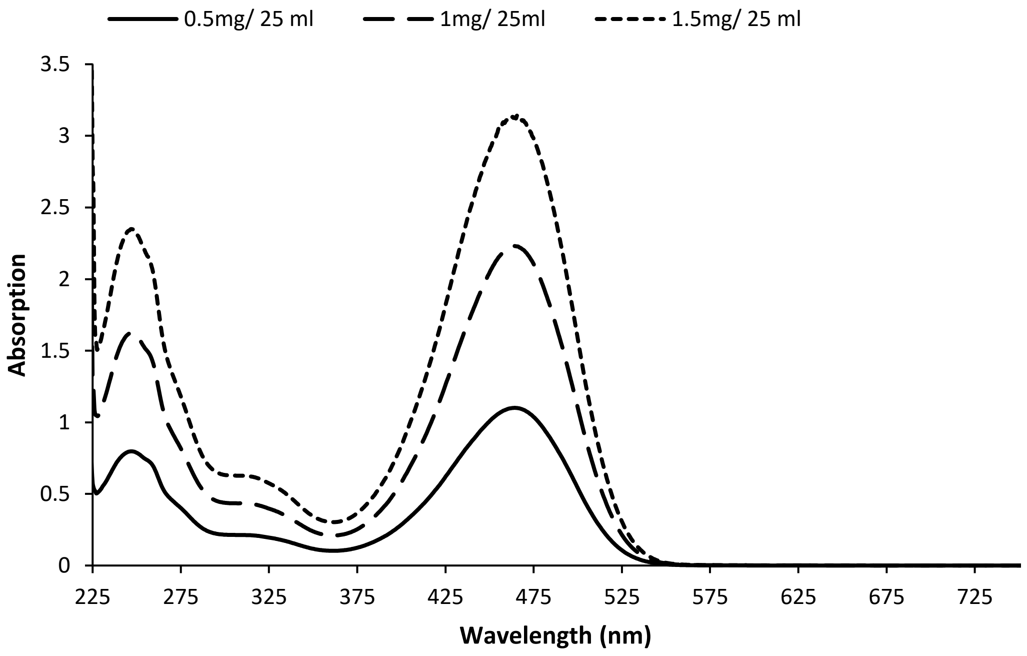

Figure S9 Electronic absorption spectra of different concentration H**L** in CH_2Cl_2.

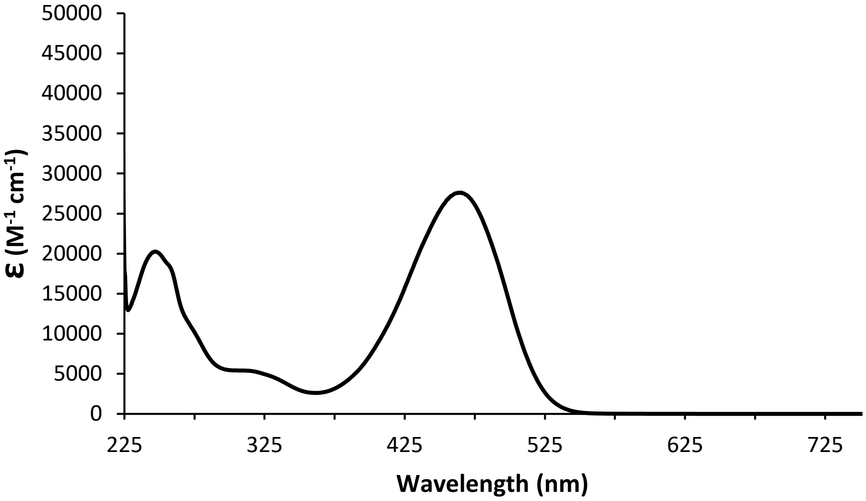

Figure S10 Electronic absorption spectrum of H**L** in CH_2Cl_2.

2.4 Uranyl acetate complex $UO_2(OAc)(\mathbf{L})$

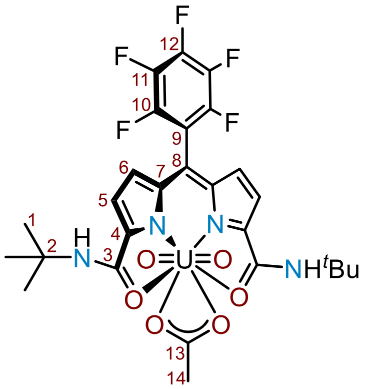

$UO_2(OAc)(\mathbf{L})$

A solution of H**L** (100 mg, 0.197 mmol, 1 eq.; in MeOH:$CHCl_3$, 1:3, 70 mL) was added to a solution of $UO_2(OAc)_2 \cdot 2H_2O$ (91.8 mg, 0.217 mmol, 1.1 eq.; in MeOH:$CHCl_3$, 1:3, 20 mL) after which NEt_3 was added (36 µL, 0.256 mmol, 1.3 eq.), causing an immediate colour change from orange to pink. The reaction mixture was heated to 65 °C and stirred for 18 hrs, after which solvent was removed under reduced pressure. The oil was redissolved in CH_2Cl_2 (75 mL), washed with H_2O (3 x 15 mL) and dried with $MgSO_4$. A greenish pink solid was obtained. Greenish-pink single crystals suitable for X-ray crystallography were obtained through slow evaporation of a concentrated THF solution. Yield = 127 mg (77 %). ^{1}H NMR (400 MHz, Methanol-d_4): δ_H 7.59 (2H, d, J = 4.5 Hz, C(5)H), 7.15 (2H, d, J = 4.5 Hz, c(6)H), 2.17 (3H, bs, C(14)H_3), 1.80 (18H, s, C(1)H_3). ^{13}C{^{1}H} NMR (126 MHz, Methanol-d_4): δ_C 169.76 (C(3)), 158.86 (C(4)), 144.80 (d, J = 230.5 Hz, C(12)F), 143.44 (C(7)), 142.21 (d, J = 250.4 Hz, C(11)F), 137.67 (d, J = 246.6 Hz, C(10)F), 137.41 (C(8)), 133.53 (C(6)H), 128.46 (C(9)), 119.00 (C(5)H), 54.04 (C(2)), 27.70 (C(1)H_3). ^{19}F{^{1}H} NMR (376 MHz, Methanol-d_4): δ_F -141.82 (2F, dd, J = 21.3, 5.9 Hz, Ar^F ortho-F), -155.06 (1F, t, J = 20.6 Hz, Ar^F para-F), -163.97 (2F, td, J = 20.7, 6.0 Hz, Ar^F meta-F). MS (ESI$^+$, MeOH): m/z $C_{27}H_{28}F_5N_4O_6U$ $[M+H]^+$ requires 837.24314, found 837.25460 (mass error = 11.46 ppm), $C_{27}H_{27}F_5N_4O_6UNa$ $[M+Na]^+$ requires 859.22508, found 859.22830 (mass error = 3.22 ppm), $C_{27}H_{24}F_5N_4O_4U$ $[M-OAc]^+$ requires 777.22201, found 777.22640 (mass error = 5.64 ppm). EA for $C_{27}H_{27}F_5N_4O_6U$ (MW = 836.24 gmol^{-1}) requires C,38.77 %; H,3.25 %; N,6.70 %. Found C,38.83 %; H,3.35 %; N,6.51 %. FTIR (film) ν cm^{-1} 2962 (w), 2925 (w), 1590 (s), 1575 (s), 1520 (s), 1501 (s), 1495 (m), 1370 (m), 1352 (m), 1332 (w), 1292 (m), 1247 (s), 1199 (s), 1072 (m), 1005 (s), 979 (s), 951 (m), 905 (s), 837 (s), 805 (m), 758 (m), 743 (m), 725 (m), 713 (m), 645 (m). UV-vis (THF): λ 512 nm, ε = 20.812 M^{-1} cm^{-1}; λ_{max} 546.5 nm, ε = 82.316 M^{-1} cm^{-1}.

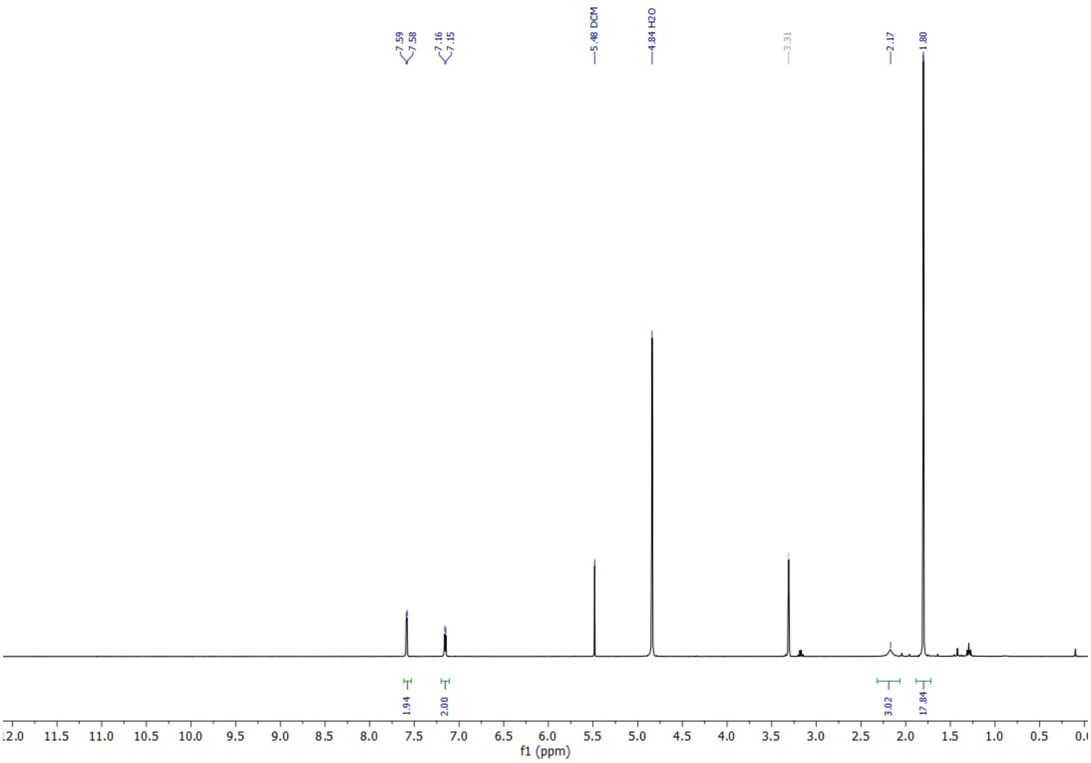

Figure S11 ^{1}H NMR of UO_2(OAc)(**L**) in methanol-d_4.

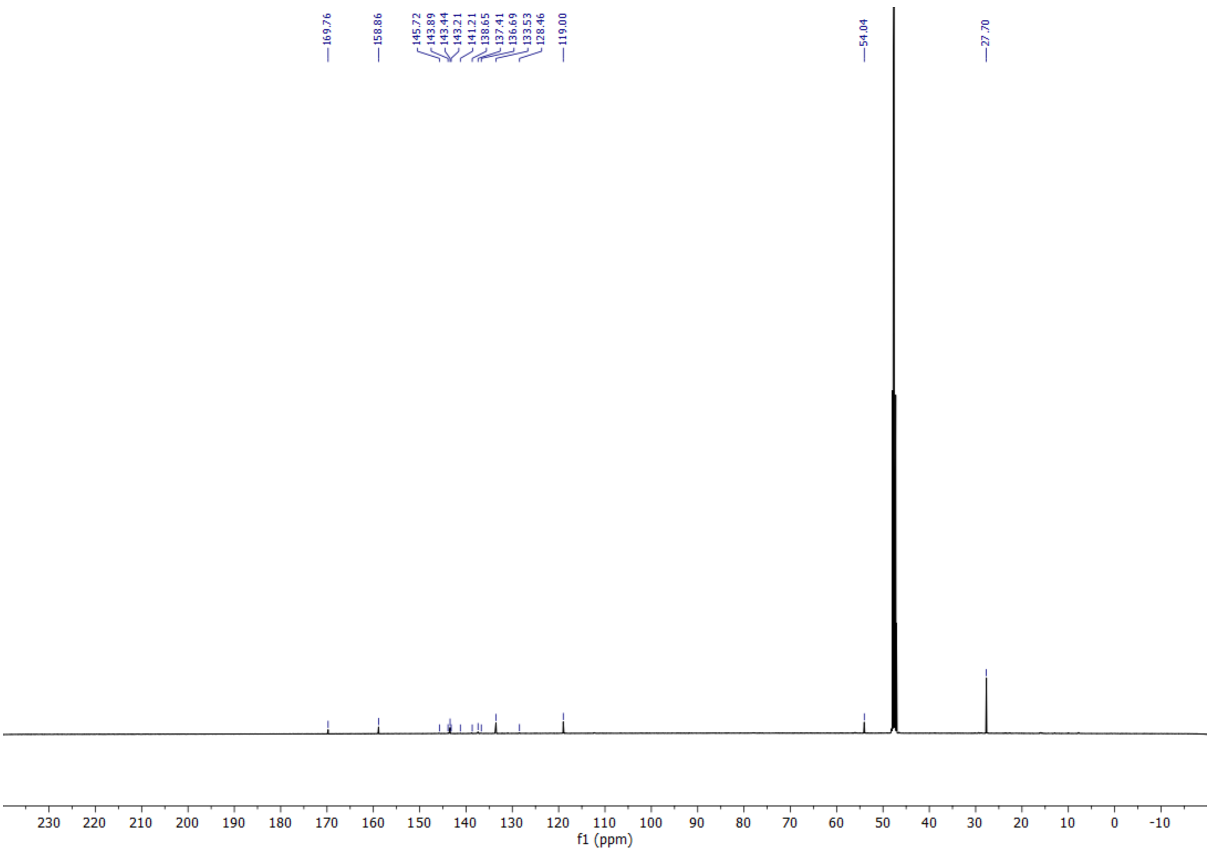

Figure S12 $^{13}C\{^{1}H\}$ NMR of UO_2(OAc)(**L**) in methanol-d_4.

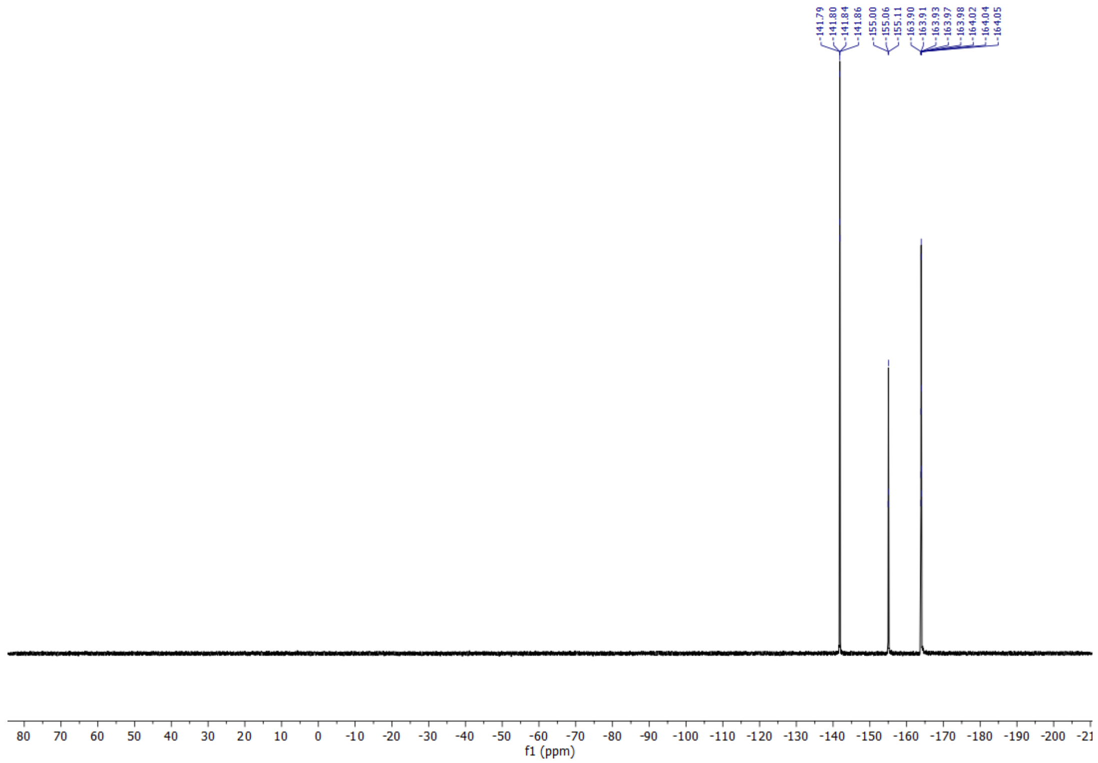

Figure S13 $^{19}F\{^1H\}$ NMR of $UO_2(OAc)(\mathbf{L})$ in methanol-d_4.

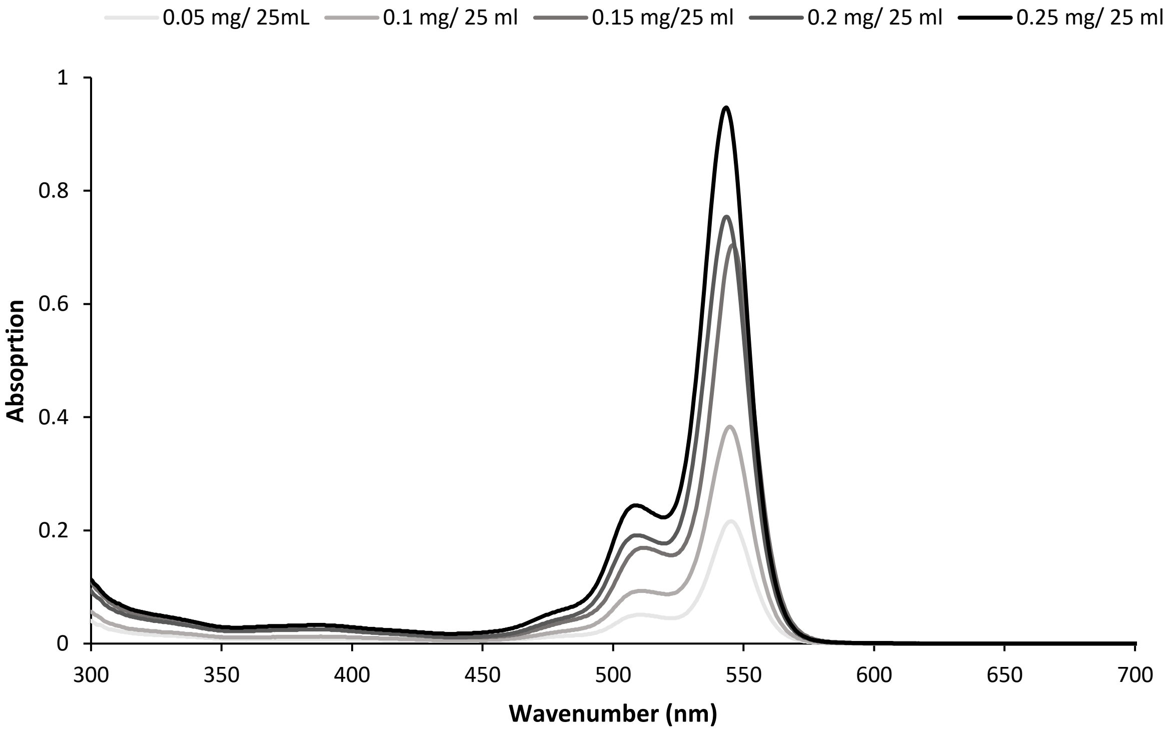

Figure S14 Electronic absorption spectra of different concentration $UO_2(OAc)(\mathbf{L})$ in THF.

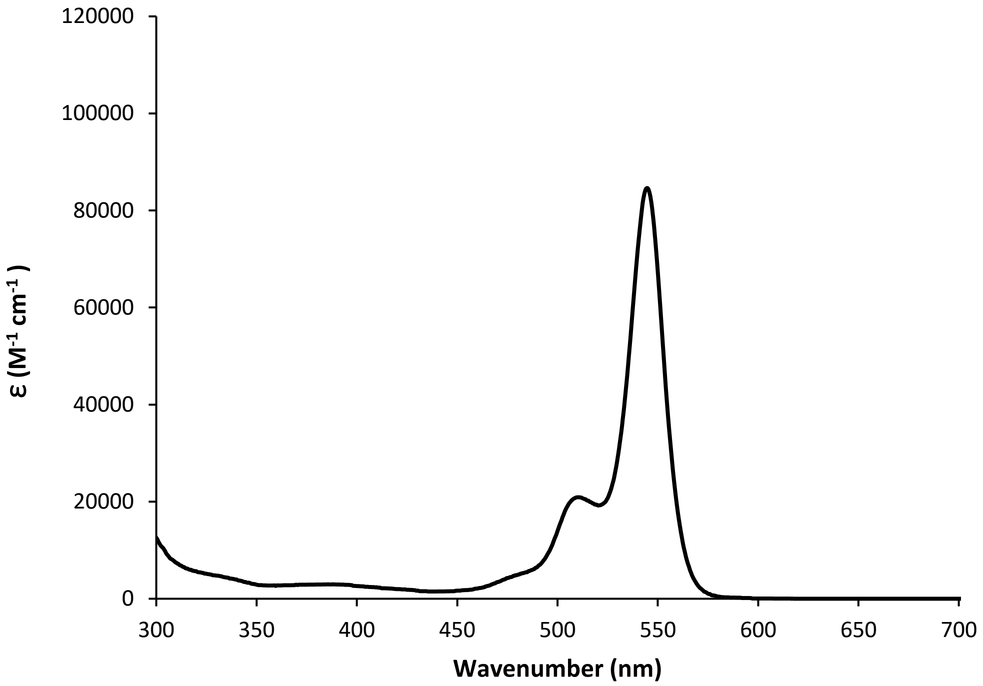

Figure S15 Electronic absorption spectrum of UO_2(OAc)(**L**) in THF.

2.5 Uranyl chloride complex $UO_2Cl(\mathbf{L})$

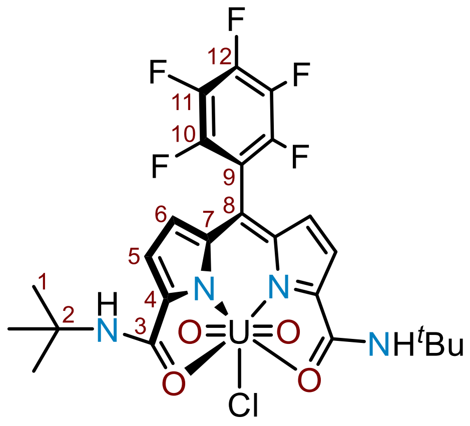

$UO_2Cl(\mathbf{L})$

Method A

A solution of H**L** (131 mg, 0.257 mmol, 1 eq.; in MeOH:$CHCl_3$, 1:3, 150 mL) was added to a solution of $UO_2Cl_2THF_2$ (137 mg, 0.283 mmol, 1.1 eq.; in MeOH:$CHCl_3$, 1:3, 20 mL) after which NEt_3 was added (47 µL, 0.334 mmol, 1.3 eq.), causing an immediate colour change from orange to red. The reaction mixture was heated to 65 °C and stirred for 18 hrs, after which solvent was removed under reduced pressure. The majority of the red solid was redissolved in CH_2Cl_2 (400 mL) and filtered. The filtrate was washed with H_2O (3 x 50 mL) and dried with $MgSO_4$ and concentrated so obtain a red solid. The red solid and residue was combined yielding a red solid. Yield = 189 mg (91 %). ^{1}H NMR (400 MHz, Acetonitrile-d_3): δ_H 8.22 (2H, s, C(3)ON*H*), 7.46 (2H, d, *J* = 4.4 Hz, C(5)*H*), 7.19 (2H, d, *J* = 4.4 Hz, C(6)*H*), 1.76 (18H, s, C(1)H_3). ^{13}C{^{1}H} NMR (126 MHz, Acetonitrile-d_3): δ_C 169.78 (*C*(3)), 159.02 (*C*(4)), 144.81 (d, *J* = 247.5 Hz, Ar^F *C*(12)F), 143.17 (*C*(7)), 142.42 (d, *J* = 248.2 Hz, Ar^F *C*(11)F), 138.05 (d, J = 251.5 Hz, Ar^F *C*(10)F) 137.56 (*C*(8)), 134.23 (*C*(6)H), 132.34 (*C*(9)), 119.57 (*C*(5)H), 55.02 (*C*(2)), 27.84 (*C*(1)H_3). ^{19}F{^{1}H} NMR (376 MHz, Acetonitrile-d_3): δ_F -140.88 – -143.36 (m, Ar^F *ortho*-F), -154.28 – -155.13 (m, Ar^F *para*-F), -162.18 – -163.97 (m, Ar^F *meta*-F). MS (ESI$^+$, MeOH): *m/z* $C_{25}H_{25}F_5N_4O_4ClU$ $[M+H]^+$ requires 813.19869, found 813.19580 (mass error = -2.89 ppm). FTIR (film) ν cm^{-1} 3300 (w), 3270 (w), 2972 (w), 1592 (s), 1570 (s), 1521 (s), 1489 (s), 1460 (m), 1374 (m), 1370 (m), 1348 (m), 1291(m), 1264 (s), 1200 (s), 1164 (m), 1075 (s), 1053 (w), 1007 (s), 974 (s), 978 (s), 950 (m), 912 (s), 839 (s), 804 (m), 771 (m), 743 (s), 726 (m), 714 (m), 647 (m). UV-vis (THF): λ 514 nm, ε = 18.582 M^{-1} cm^{-1}; λ_{max} 546 nm, ε = 84.301 M^{-1} cm^{-1}.

Method B

A solution of HL (35 mg, 0.068 mmol, 1.0 eq.) in dry THF (3 mL) was dropwise added to a slurry of KH (3 mg, 0.0746, 1.1 eq.) in dry THF (2 mL). The solution slowly turned pinkish red and was left stirred overnight after which it was dropwise added to a yellow slurry of $UO_2Cl_2THF_2$ (32 mg, 0.068 mmol, 1.0 eq.) in dry THF (2 mL), causing an immediate colour change from red to pink. The reaction mixture was stirred at room temperature for 18 hours, after which the reaction mixture was transferred to the bench and solved was removed under reduced pressure. The solid partially dissolved in CH_2Cl_2 (30 mL). The filtrate was washed with H_2O (3 x 3 mL), dried over $MgSO_4$ and concentrated. Both residue and washed filtrate were combined, obtaining a red solid.

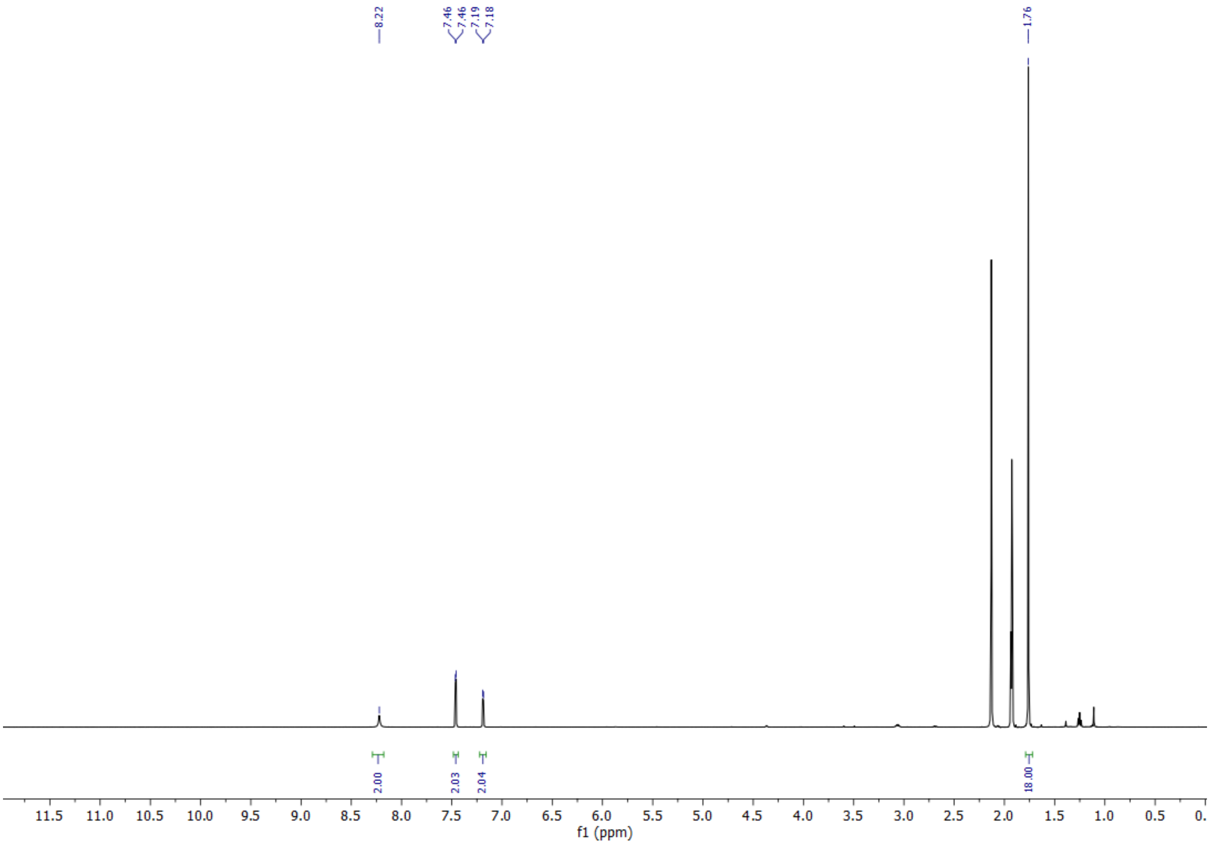

Figure S16 ^{1}H NMR of UO_2(Cl)(**L**) in acetonitrile-d_3.

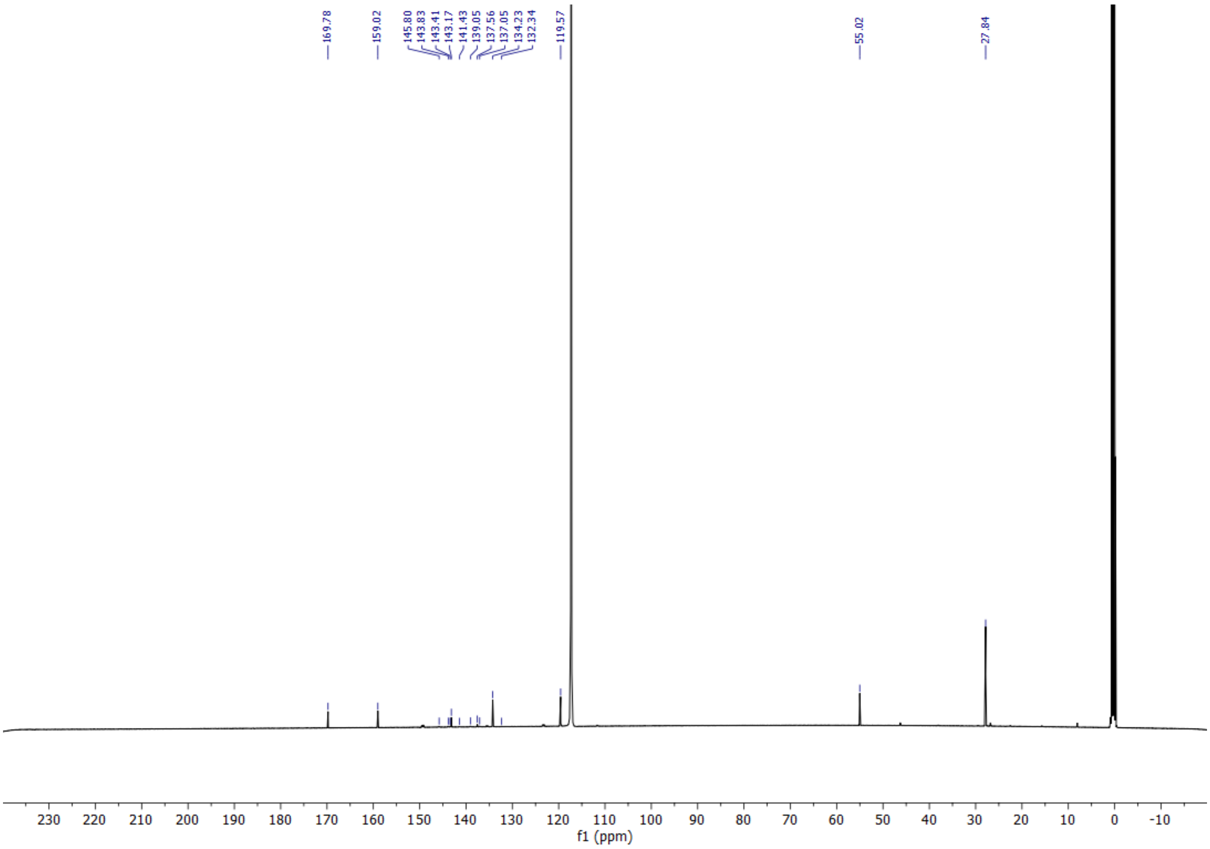

Figure S17 $^{13}C\{^1H\}$ NMR of $UO_2Cl(\mathbf{L})$ in acetonitrile-d_3

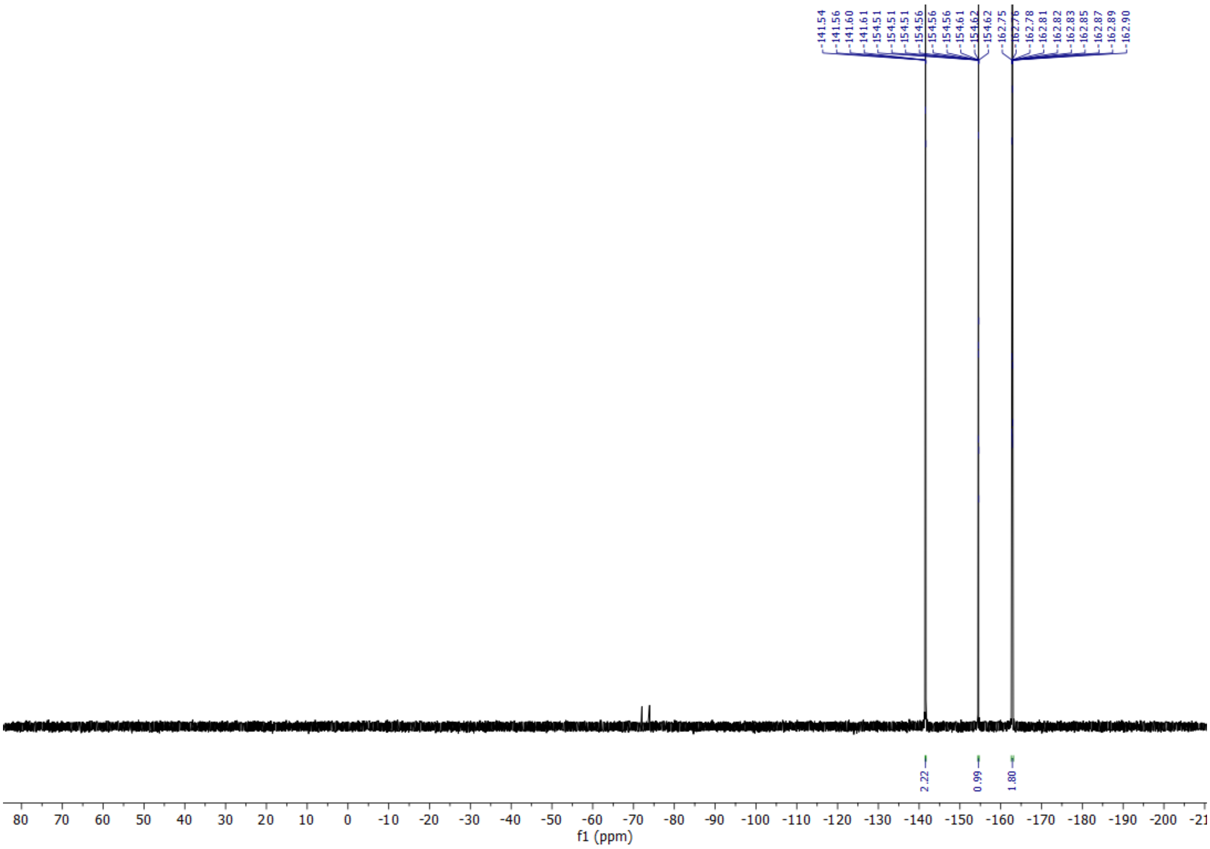

Figure S18 $^{19}F\{^1H\}$ NMR of $UO_2Cl(\mathbf{L})$ in acetonitrile-d_3

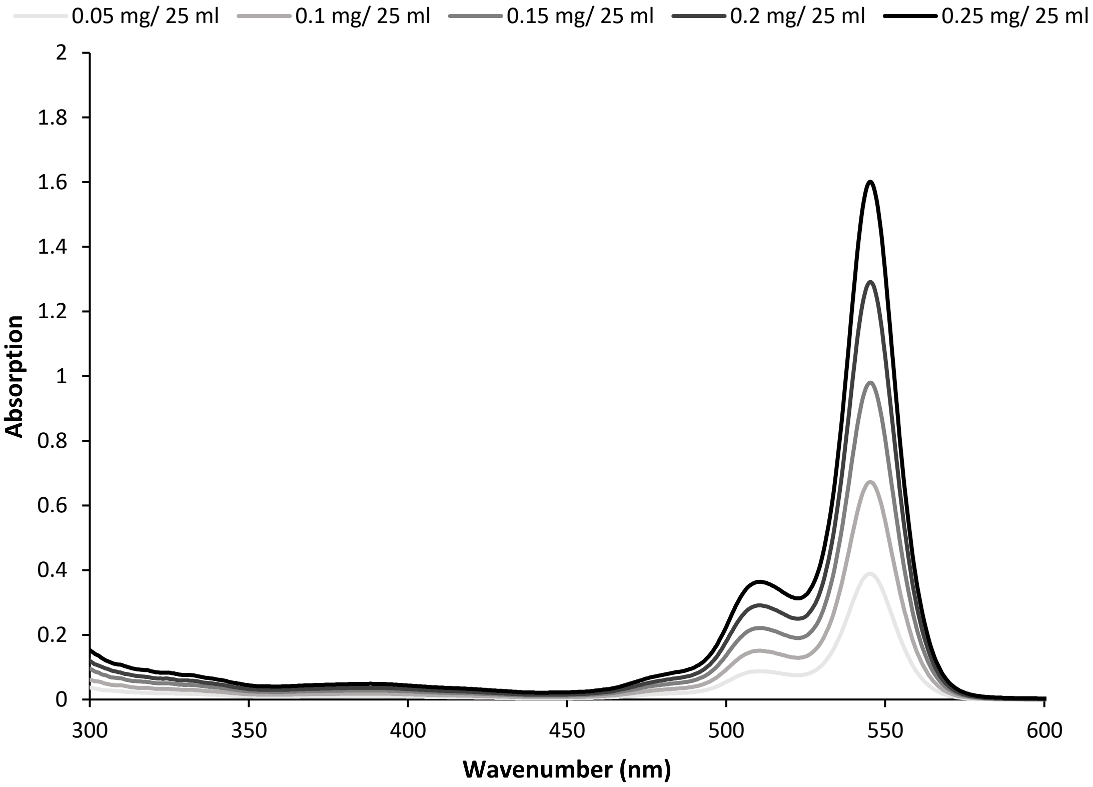

Figure S19 Electronic absorption spectra of different concentration UO_2Cl(**L**) in THF.

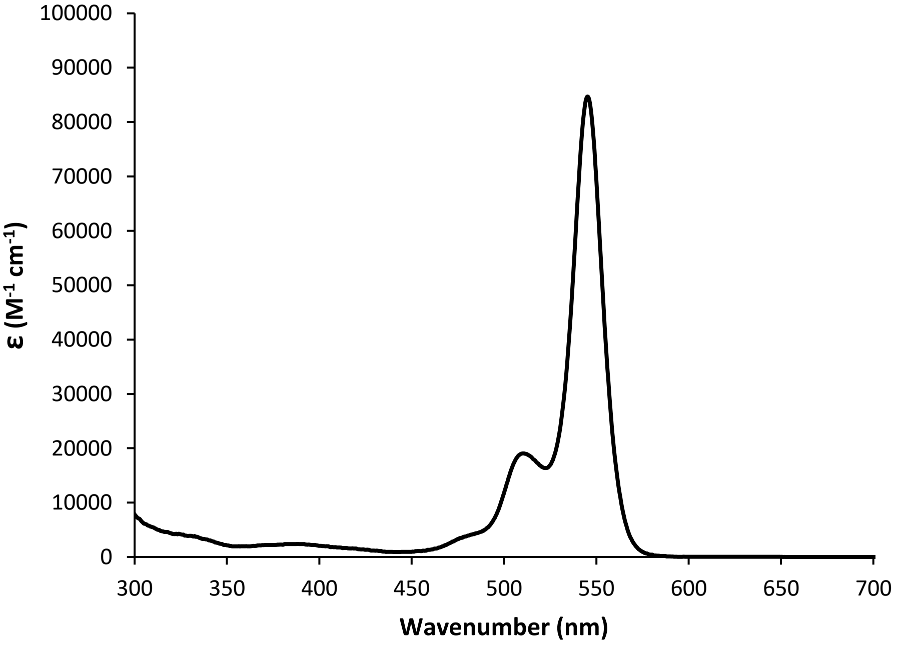

Figure S20 Electronic absorption spectrum of UO_2Cl(**L**) in THF.

2.6 [Cp_2Co][UO_2(OAc)(**L•**)]

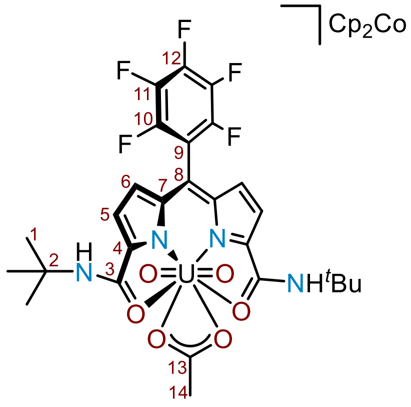

[Cp_2Co][UO_2(OAc)**L•**]

A pink solution of UO_2(OAc)(**L**) (50 mg, 0.06 mmol, 1.0 eq.) in dry THF (5 mL) was added to a solution of $CoCp_2$ (11.3 mg, 0.06 mmol, 1.0 eq) in dry THF (1 mL). The solution turned dark greenish red instantaneously and a green precipitate started forming. The reaction was left stirring for 1 hr before centrifuging. Solids were obtained as a greenish brown solid. Yield = 46 mg (75 %). NMR silence. EPR S = ½, and g_{iso} of 1.997. EA for $C_{37}H_{37}CoF_5N_4O_6U$ (MW = 1025.25 gmol^{-1}) requires C,43.33 %; H,3.63 %; N,5.46 %. Found C,43.44 %; H,3.50 %; N,5.44 %. UV-vis (pyridine): λ 551 nm, ε = 10 500 M^{-1} cm^{-1}; λ_{max} 500 nm, ε = 45 700 M^{-1} cm^{-1}.

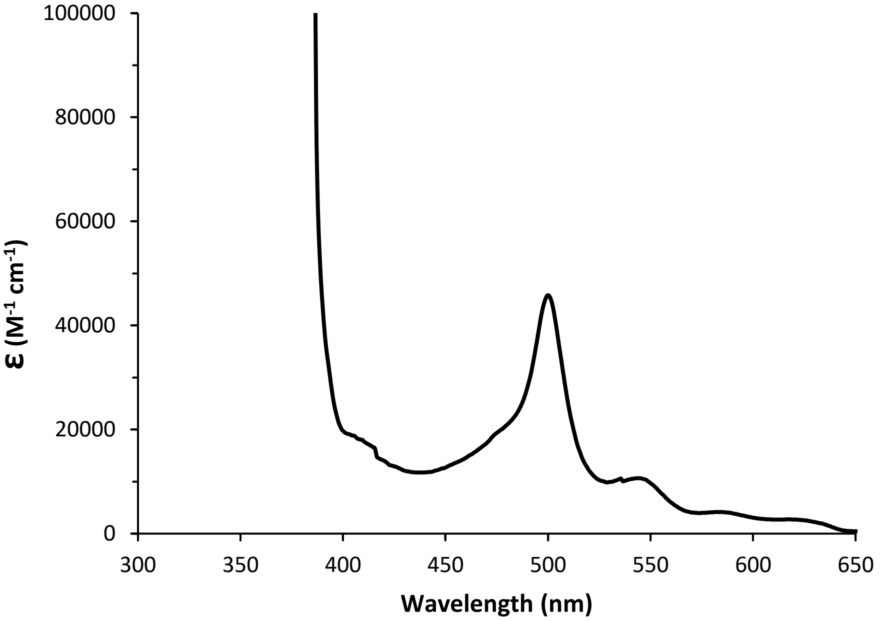

Figure S21 Electronic absorption spectrum of [Cp_2Co][UO_2(OAc)(L•)] in pyridine.

2.7 [Cp_2Co][UO_2Cl(**L**•)]

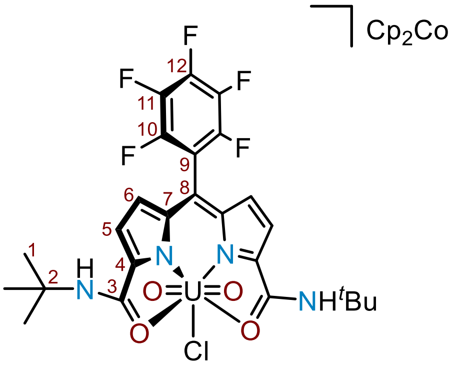

[Cp_2Co][UO_2Cl**L**•]

[Cp_2Co][UO_2Cl(**L**•)]: A pink solution of UO_2(Cl)(**L**) (49 mg, 0.06 mmol, 1.0 eq.) in dry THF (5 mL) was added to a solution of $CoCp_2$ (11.3 mg, 0.06 mmol, 1.0 eq) in dry THF (1 mL). The solution turned dark greenish red instantaneously and a green precipitate started forming. The reaction was left stirring for 1 hr before centrifuging. Solids were obtained as a greenish brown solid. Yield = 55 mg (91 %). NMR silence. EPR S = ½, and g_{iso} of 1.997. EA for $C_{35}H_{34}ClCoF_5N_4O_4U$ (MW = 1002.09 gmol^{-1}) requires C,41.95 %; H,3.42 %; N,5.59 %. Found C,41.34 %; H,3.31 %; N,5.30 %. UV-vis (pyridine): λ 551 nm, ε = 10 500 M^{-1} cm^{-1}; λ_{max} 500 nm, ε = 45 700 M^{-1} cm^{-1}.

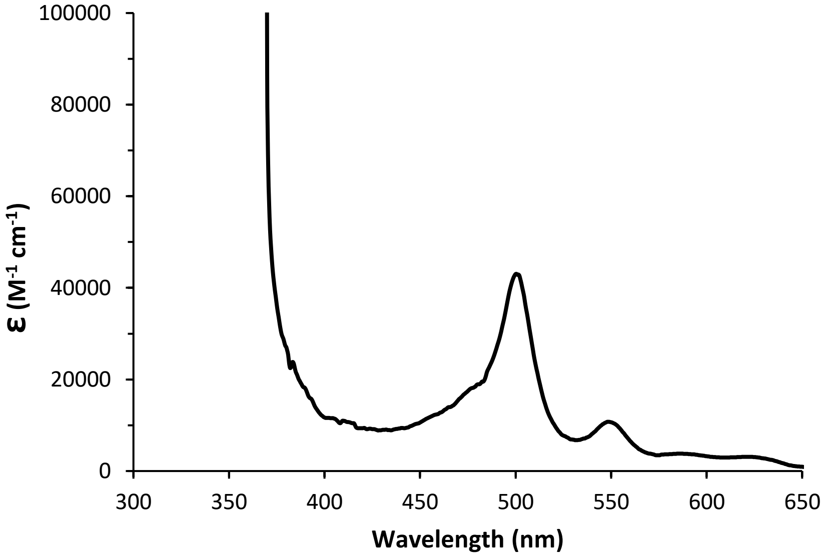

Figure S22 Electronic absorption spectrum of [Cp_2Co][UO_2Cl(L•)] in pyridine.

3 Crystallography

Table S1 Crystal data for H**L**. Crystallised as the solvate from a concentrated DMSO solution.

**Crystal data**	
CCDC 2122706	
Chemical formula	$C_2H_6OS{\cdot}C_{25}H_{25}F_5N_4O_2$
M_r	586.62
Crystal system, space group	Triclinic, *P*1
Temperature (K)	170
a, *b*, *c* (Å)	9.9616 (12), 10.2726 (12), 14.926 (2)
α, β, γ (°)	75.169 (11), 75.089 (11), 81.929 (10)
V (Å^3)	1422.3 (3)
Z	2
Radiation type	Mo *K*α
μ (mm^{-1})	0.18
Crystal size (mm)	0.15 × 0.14 × 0.07
**Data collection**	
Diffractometer	Xcalibur, Eos
Absorption correction	Analytical<br>*CrysAlis PRO* 1.171.40.54a (Rigaku Oxford Diffraction, 2019) Analytical numeric absorption correction using a multifaceted crystal model based on expressions derived by R.C. Clark & J.S. Reid. (Clark, R. C. & Reid, J. S. (1995). Acta Cryst. A51, 887-897) Empirical absorption correction using spherical harmonics, implemented in SCALE3 ABSPACK scaling algorithm.
T_{min}, T_{max}	0.986, 0.993
No. of measured, independent and observed [$I > 2\sigma(I)$] reflections	19136, 3356, 2062
R_{int}	0.107
θ_{max} (°)	21.7
$(\sin \theta/\lambda)_{max}$ (Å^{-1})	0.521
**Refinement**	
$R[F^2 > 2\sigma(F^2)]$, $wR(F^2)$, S	0.070, 0.177, 1.04
No. of reflections	3356
No. of parameters	368
H-atom treatment	H atoms treated by a mixture of independent and constrained refinement
$\Delta\rho_{max}$, $\Delta\rho_{min}$ (e Å^{-3})	0.59, −0.31

Table S2 Crystal data for $UO_2(OAc)$(**L**). Crystallised as the solvate from a concentrated THF solution.

Crystal data	
CCDC 2122705	
Chemical formula	$C_{27}H_{27}F_5N_4O_6U$
M_r	836.55
Crystal system, space group	Monoclinic, $P2_1/n$
Temperature (K)	120
a, b, c (Å)	17.6861 (5), 18.0840 (4), 22.0970 (9)
β (°)	106.225 (4)
V (Å^3)	6785.9 (4)
Z	8
Radiation type	Mo Kα
μ (mm^{-1})	4.85
Crystal size (mm)	0.35 × 0.28 × 0.09
Data collection	
Diffractometer	SuperNova, Dual, Cu at home/near, Atlas
Absorption correction	Multi-scan<br>*CrysAlis PRO* 1.171.40.84a (Rigaku Oxford Diffraction, 2020) Empirical absorption correction using spherical harmonics, implemented in SCALE3 ABSPACK scaling algorithm.
T_{min}, T_{max}	0.311, 1.000
No. of measured, independent and observed [$I > 2\sigma(I)$] reflections	97983, 12402, 9836
R_{int}	0.071
$(\sin \theta/\lambda)_{max}$ (Å^{-1})	0.602
Refinement	
$R[F^2 > 2\sigma(F^2)]$, $wR(F^2)$, S	0.046, 0.101, 1.06
No. of reflections	12402
No. of parameters	789
No. of restraints	1076
H-atom treatment	H-atom parameters constrained
	$w = 1/[\sigma^2(F_o^2) + (0.0287P)^2 + 46.4684P]$<br>where $P = (F_o^2 + 2F_c^2)/3$
$\Delta\rho_{max}$, $\Delta\rho_{min}$ (e Å^{-3})	3.18, −1.23

Table S3 Crystal data for UO_2Cl(**L**). Crystallised as the solvate from a concentrated THF solution.

**Crystal data**	
**CCDC 2122704**	
Chemical formula	$C_{25}H_{24}ClF_5N_4O_4U·1.5(C_4H_8O)·0.25(C_4O_2)$
M_r	941.13
Crystal system, space group	Monoclinic, $P2_1/n$
Temperature (K)	100
a, *b*, *c* (Å)	18.4999 (4), 15.3604 (3), 24.8107 (6)
β (°)	98.655 (1)
V (Å^3)	6970.1 (3)
Z	8
Radiation type	Mo *K*α
μ (mm^{-1})	4.81
Crystal size (mm)	0.18 × 0.15 × 0.04
**Data collection**	
Diffractometer	Bruker *APEX*-II CCD
Absorption correction	Multi-scan<br>*SADABS2016*/2 (Bruker,2016/2) was used for absorption correction. wR2(int) was 0.0686 before and 0.0481 after correction. The Ratio of minimum to maximum transmission is 0.8239. The λ/2 correction factor is Not present.
T_{min}, T_{max}	0.615, 0.747
No. of measured, independent and observed [$I > 2σ(I)$] reflections	109436, 28284, 20697
R_{int}	0.051
$(\sin θ/λ)_{max}$ (Å^{-1})	0.794
**Refinement**	
$R[F^2 > 2σ(F^2)]$, $wR(F^2)$, S	0.036, 0.069, 1.02
No. of reflections	28284
No. of parameters	895
H-atom treatment	H-atom parameters constrained
$Δρ_{max}$, $Δρ_{min}$ (e Å^{-3})	1.42, −1.19

4 Optimised DFT geometries

Computational details

The optimization of different spin states for uranium complexes was carried out by employing DFT hybrid functional (B3PW91)[13] along with small core pseudopotential Stuttgart basis set for uranium, chlorine with additional polarization functions for chlorine atom.[14] Pople basis sets (6-31G** for carbon, nitrogen, oxygen, hydrogen atoms) were employed for the rest of the atoms.[15] Frequency calculations were performed to locate minima for the optimized structures. Dispersion corrections were included in our calculations by employing D3 version of Grimme's dispersion with Becke-Johnson damping.[16] All the calculations were performed using Gaussian 09 suite of programs.[17]

4.1 UO_2Cl(**L**)

C	13.491925000	2.837415000	4.850693000
H	12.615384000	2.351263000	5.291105000
H	14.240417000	2.069464000	4.632208000
H	13.200013000	3.313641000	3.915274000
C	15.336905000	4.529414000	5.271601000
H	15.133575000	5.018421000	4.319852000
H	16.110101000	3.771962000	5.110707000
H	15.718409000	5.273629000	5.976635000
C	14.457121000	3.134068000	7.128136000
H	14.841550000	3.822641000	7.888410000
H	15.252156000	2.420609000	6.897589000
H	13.627104000	2.559470000	7.552805000
C	14.077221000	3.859873000	5.831860000
C	12.473249000	5.022599000	7.151773000
H	12.681034000	4.329890000	7.967583000
C	11.510339000	6.050918000	7.441685000
C	10.714520000	6.155453000	8.618055000
H	10.734291000	5.479429000	9.462048000
C	9.933313000	7.272115000	8.442852000
H	9.205203000	7.683963000	9.127043000
C	10.283060000	7.824310000	7.167567000
C	9.738253000	8.985388000	6.611428000
C	8.642822000	9.641534000	7.373496000
C	8.852439000	10.842611000	8.050704000
C	7.832719000	11.459457000	8.767630000
C	6.573127000	10.868763000	8.813291000
C	6.339022000	9.669782000	8.146112000
C	7.372613000	9.068265000	7.435697000
C	10.131728000	9.593166000	5.416281000
C	9.572620000	10.794043000	4.868825000
H	8.753191000	11.356395000	5.293169000
C	10.277602000	11.071919000	3.722928000
H	10.145299000	11.901334000	3.041562000
C	11.234523000	10.024925000	3.593634000
C	12.208576000	9.878710000	2.545372000
H	12.312321000	10.732295000	1.874734000
C	13.971435000	8.807399000	1.359107000
C	14.288934000	10.195335000	0.787628000
H	13.460642000	10.621399000	0.211940000
H	15.132436000	10.096369000	0.099844000
H	14.579858000	10.902441000	1.571933000
C	13.461281000	7.910963000	0.224729000
H	13.217939000	6.914485000	0.591936000
H	14.233436000	7.821230000	-0.545691000
H	12.567647000	8.350321000	-0.230507000
C	15.249965000	8.248078000	1.991217000
H	15.579820000	8.886677000	2.815518000
H	16.042471000	8.213135000	1.237490000
H	15.093028000	7.239103000	2.370311000
N	13.058588000	4.927426000	6.013617000
N	11.262675000	7.052310000	6.579121000
N	11.152997000	9.143330000	4.605628000
N	12.928258000	8.823515000	2.420218000
O	10.837523000	6.394378000	3.770820000
O	13.897703000	7.604214000	4.978549000
F	10.052557000	11.421204000	8.028206000
F	8.053467000	12.603474000	9.411519000
F	5.592384000	11.450702000	9.495232000
F	5.131974000	9.110847000	8.189578000
F	7.124863000	7.925022000	6.799577000
Cl	13.413700000	5.351764000	2.511913000
U	12.389224000	6.972389000	4.340096000

4.2 $[UO_2$(**L**)$]^+$

C	13.760107000	3.624313000	4.320496000
H	12.828914000	3.074570000	4.478677000
H	14.516233000	2.932254000	3.940792000
H	13.573467000	4.373804000	3.544132000
C	15.523392000	5.077681000	5.381862000
H	15.378689000	5.835129000	4.603153000
H	16.327848000	4.419523000	5.043784000
H	15.845669000	5.589601000	6.291880000
C	14.510119000	3.185550000	6.671288000
H	14.856160000	3.609704000	7.619156000
H	15.296482000	2.522150000	6.303580000
H	13.625471000	2.568353000	6.856284000
C	14.245529000	4.266291000	5.626552000
C	12.553241000	5.139693000	7.128239000
H	12.735422000	4.332624000	7.837806000
C	11.543161000	6.110332000	7.463141000
C	10.755369000	6.191803000	8.640620000
H	10.793011000	5.514584000	9.482593000
C	9.955877000	7.301782000	8.484364000
H	9.233581000	7.693099000	9.185933000
C	10.267681000	7.871121000	7.208931000
C	9.683133000	9.014136000	6.644261000

C	8.595965000	9.670497000	7.407336000	H	12.469576000	7.178422000	0.460935000
C	8.761257000	10.954484000	7.933431000	H	13.898928000	7.683476000	-0.464188000
C	7.748517000	11.578166000	8.652765000	H	12.605225000	8.842047000	-0.138980000
C	6.538911000	10.914244000	8.849448000	C	14.667509000	7.217198000	2.132750000
C	6.348257000	9.634299000	8.331813000	H	15.173884000	7.490142000	3.067732000
C	7.375442000	9.024512000	7.620987000	H	15.446071000	6.893948000	1.437815000
C	10.043796000	9.587714000	5.413002000	H	14.013697000	6.343111000	2.275337000
C	9.419481000	10.715534000	4.791860000	N	13.207537000	5.240447000	6.023846000
H	8.577469000	11.263769000	5.188708000	N	11.252181000	7.117721000	6.604821000
C	10.080638000	10.944682000	3.605430000	N	11.064852000	9.132638000	4.609077000
H	9.881669000	11.715006000	2.873379000	N	12.869717000	8.723544000	2.611665000
C	11.087925000	9.949921000	3.524763000	O	11.512141000	6.271857000	3.689592000
C	12.055076000	9.714195000	2.489902000	O	13.832806000	8.195671000	5.463398000
H	12.070788000	10.379654000	1.624263000	F	9.915491000	11.597919000	7.764902000
C	13.877116000	8.404751000	1.576537000	F	7.928377000	12.793024000	9.154003000
C	14.811047000	9.598047000	1.363413000	F	5.568368000	11.501027000	9.529908000
H	14.276085000	10.456272000	0.947624000	F	5.192605000	9.009379000	8.514698000
H	15.602297000	9.329685000	0.658245000	F	7.172159000	7.806000000	7.122352000
H	15.274584000	9.902390000	2.306198000	U	12.609587000	7.269301000	4.620346000
C	13.165975000	8.002717000	0.281677000				

4.3 $UO_2(OAc)(\mathbf{L})$

U	5.384651	2.220436	14.883656	C	2.398067	1.067199	15.756376
O	7.548118	3.157155	14.256828	C	2.509863	-1.702795	17.155105
F	1.928287	2.948551	9.845474	H	2.077189	-2.246462	16.308891
O	6.704242	1.194033	16.266119	H	2.602135	-2.397152	17.996593
O	5.563957	0.936589	13.684072	H	3.508914	-1.361864	16.877008
O	5.115124	3.558737	16.003785	C	0.631172	6.260757	9.230792
O	3.612222	0.981450	16.059319	C	6.418017	5.230916	11.394903
N	5.354789	3.894162	12.925119	H	7.211841	5.745023	10.867063
F	-0.105057	6.900001	8.327877	C	1.677796	4.245052	10.023838
N	2.980934	2.607638	14.022460	C	5.076548	5.273152	11.106968
F	0.427147	4.249028	8.016937	H	4.595920	5.816193	10.304643
F	2.362401	6.949484	12.322425	C	1.616669	-0.522389	17.555606
N	8.923840	4.397273	12.946424	C	10.472990	2.571335	13.556963
H	8.958452	5.050787	12.178551	H	9.733736	2.019150	14.140140
N	1.439385	0.392018	16.404426	H	11.462778	2.359745	13.974921
H	0.496132	0.521147	16.070180	H	10.455111	2.212399	12.522118
F	0.858056	8.241446	10.490327	C	6.814988	-0.487170	17.950858
O	8.701939	0.790571	17.191568	H	5.858337	-0.119845	18.331285
C	7.716339	3.939148	13.296015	H	7.464446	-0.792942	18.772734
C	2.368983	3.362353	13.049334	H	6.605819	-1.360847	17.322079
C	6.538348	4.370273	12.524841	C	1.901240	6.269095	11.273852
C	4.419262	4.435463	12.069917	C	10.177012	4.570455	15.069302
C	7.508178	0.568717	17.108360	H	9.992205	5.649301	15.114042
C	3.040023	4.198328	12.140059	H	11.141634	4.368148	15.546453
C	2.190511	4.910075	11.138766	H	9.403017	4.050946	15.638000
C	0.901075	4.903942	9.074908	C	10.210858	4.080967	13.615426
C	1.999019	1.955773	14.657317	C	2.214553	0.249976	18.738698

H	3.196958	0.654434	18.487035
H	2.324282	-0.417391	19.599901
H	1.561880	1.079755	19.029982
C	0.951332	3.158594	13.104291
H	0.216445	3.633104	12.468236
C	11.284729	4.835787	12.826284
H	11.323470	4.502291	11.782454
H	12.267263	4.653162	13.270205
H	11.109355	5.918382	12.846093
C	1.125321	6.946119	10.337456
C	0.716613	2.266821	14.123452
H	-0.246746	1.893053	14.446708
C	0.215324	-1.024814	17.915920
H	-0.445169	-0.197245	18.201555
H	0.274965	-1.710690	18.765616
H	-0.240672	-1.570501	17.081048

4.4 UO_2(OAc)(**L°**)

U	5.289433	2.292172	14.871584
O	7.462948	3.220542	14.360051
F	1.549232	2.873908	10.028031
O	6.595606	1.230955	16.370691
O	5.542383	0.986562	13.705261
O	4.998482	3.607856	16.019615
O	3.571591	0.962728	16.017824
N	5.299355	3.944154	12.949866
F	-0.158997	6.850914	8.212065
N	2.940038	2.623262	13.975339
F	0.031753	4.131379	8.203591
F	2.658797	7.058945	11.960363
N	8.898832	4.409934	13.065416
H	8.973121	5.049354	12.290486
N	1.396408	0.417893	16.416313
H	0.443047	0.580537	16.135130
F	1.175698	8.299404	10.096963
O	8.755512	0.601380	16.283445
C	7.652901	3.979878	13.371746
C	2.323319	3.378930	13.023683
C	6.533075	4.389769	12.562826
C	4.405406	4.452383	12.053392
C	7.607385	0.480087	16.695814
C	2.999009	4.221033	12.091436
C	2.171306	4.910254	11.076892
C	0.682168	4.841229	9.135060
C	1.948902	1.975497	14.661724
C	2.349038	1.100590	15.729191
C	2.481275	-1.680942	17.118334
H	1.994913	-2.227890	16.303922
H	2.626924	-2.367894	17.959375
H	3.456150	-1.336212	16.769859
C	0.588959	6.228336	9.130820
C	6.445230	5.186765	11.402204
H	7.257087	5.663343	10.864398
C	1.468144	4.204600	10.090613
C	5.101122	5.227507	11.074077
H	4.654712	5.731245	10.226943
C	1.618916	-0.486579	17.552961
C	10.380204	2.567169	13.799700
H	9.623156	2.041728	14.386915
H	11.360543	2.356070	14.242139
H	10.375689	2.177492	12.775952
C	7.248587	-0.612260	17.700190
H	6.807946	-0.163582	18.596123
H	8.134768	-1.189680	17.970404
H	6.492442	-1.275174	17.267729
C	2.046490	6.306072	11.044132
C	10.063307	4.614208	15.233937
H	9.896860	5.697123	15.240646
H	11.001549	4.404145	15.759064
H	9.249503	4.125713	15.772223
C	10.137441	4.083436	13.794762
C	2.286993	0.267632	18.712349
H	3.255870	0.660598	18.399913
H	2.434815	-0.404413	19.565073
H	1.660391	1.105109	19.036600
C	0.906851	3.206299	13.121591
H	0.162340	3.697497	12.509251
C	11.265783	4.789769	13.034490
H	11.336129	4.423563	12.003463
H	12.224249	4.599035	13.525235
H	11.110593	5.875556	13.010686
C	1.277632	6.963702	10.088767

C	0.675666	2.316225	14.154521
H	-0.290769	1.964373	14.498283
C	0.233829	-0.980181	17.985256
H	-0.403633	-0.144772	18.297972
H	0.326715	-1.666907	18.831438
H	-0.266558	-1.515571	17.169852

4.5 UO_2(OAc)(**L°**)

U	4.631646	-0.131548	16.952696
Cl	3.556671	-2.502807	17.707231
F	6.809889	5.607764	17.546869
F	6.875829	4.063422	13.065892
F	7.830447	7.958360	16.739918
F	7.937122	6.404426	12.285810
F	8.412921	8.373990	14.109971
O	2.462340	0.603864	17.765795
O	3.970794	-0.313825	15.326795
O	6.467032	-1.628367	16.413150
O	5.322223	0.111490	18.558211
N	0.851305	2.176131	18.079638
H	0.626870	3.155063	17.997968
N	6.662650	1.014296	15.968268
N	8.603858	-2.047966	15.760166
H	9.452763	-1.638075	15.404221
N	4.231212	2.361582	16.763488
C	7.052602	2.284506	15.660966
C	7.743969	0.209154	15.733878
C	4.890266	3.453008	16.279274
C	7.573985	-1.196290	15.985360
C	2.089723	1.806394	17.671306
C	-0.175659	1.289966	18.650795
C	2.985175	2.792172	17.129269
C	8.846959	0.960001	15.274661
H	9.833950	0.594465	15.013656
C	8.415740	2.273907	15.230129
H	8.997986	3.139037	14.941176
C	7.057102	5.770416	16.245536
C	6.797650	4.731637	15.341476
C	6.224282	3.438672	15.778064
C	8.586781	-3.506689	15.956774
C	2.828946	4.171760	16.877359
H	1.951037	4.782296	17.057312
C	8.298630	-3.842766	17.427293
H	9.048089	-3.382572	18.079830
H	8.329496	-4.927995	17.575532
H	7.311355	-3.477732	17.716073
C	7.589351	6.991967	15.844352
C	4.030828	4.594103	16.337503
H	4.277046	5.593462	16.003409
C	7.111912	4.986840	13.999994
C	0.334291	0.657922	19.954482
H	-0.446449	0.029873	20.397914
H	0.605753	1.434944	20.677101
H	1.212318	0.039593	19.758582
C	-0.559467	0.199141	17.640078
H	0.302265	-0.430422	17.410823
H	-0.923150	0.648796	16.710099
H	-1.354472	-0.431012	18.054210
C	7.894734	7.205242	14.504866
C	-1.389923	2.178684	18.942530
H	-2.196822	1.580041	19.374635
H	-1.769130	2.643584	18.024800
H	-1.138348	2.970658	19.658043
C	7.535961	-4.153110	15.042130
H	6.538063	-3.789515	15.294021
H	7.553859	-5.242180	15.160838
H	7.742417	-3.915707	13.993038
C	7.657722	6.195749	13.578933
C	9.985950	-4.002357	15.574624
H	10.216846	-3.768761	14.528298
H	10.045719	-5.087474	15.696865
H	10.753660	-3.548384	16.212307

5 Calculated Molecular orbitals

5.1 Compound $U^{VI}O_2$(OAc)(**L**)

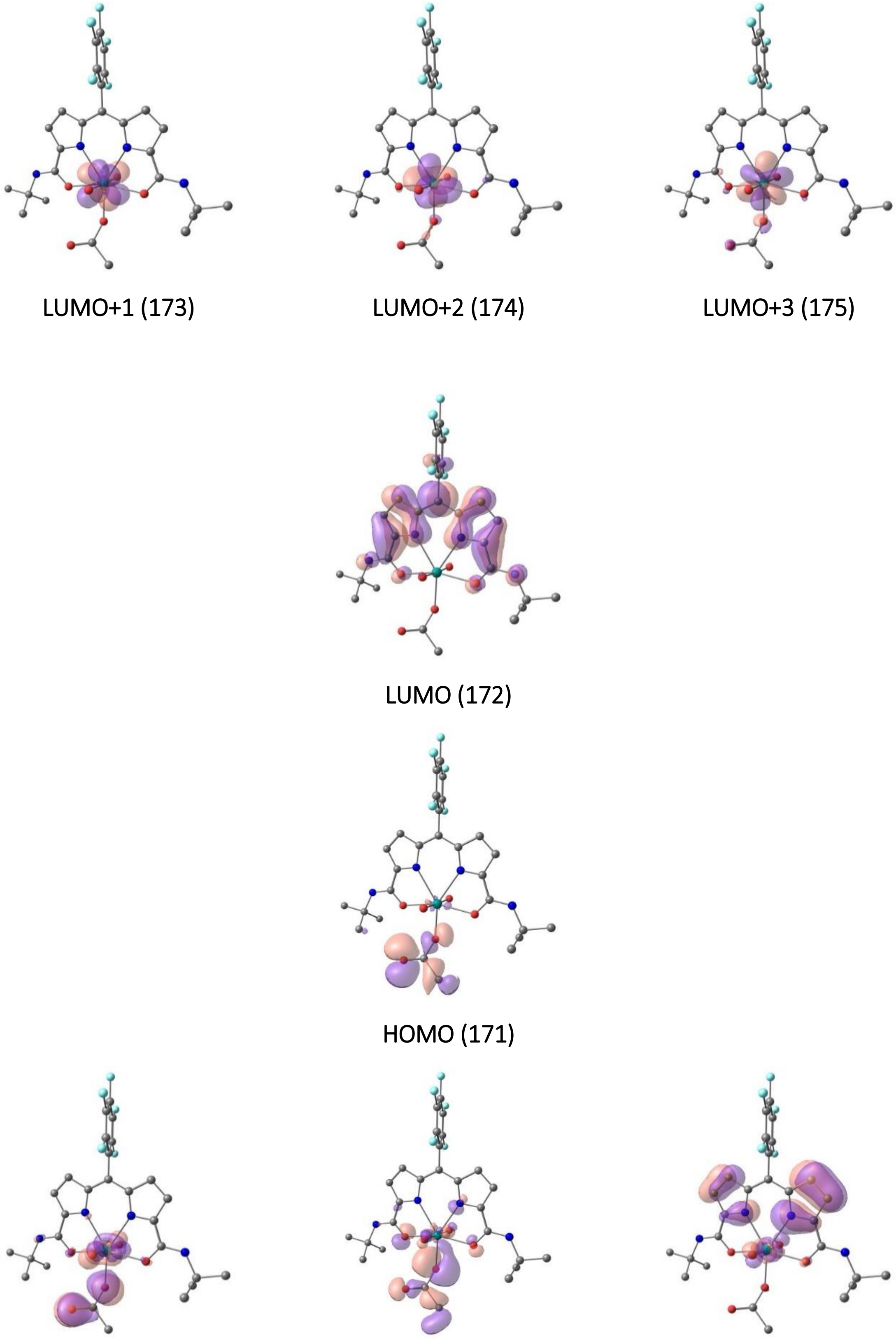

Figure S23 Molecular orbital diagram of $U^{VI}O_2$(OAc)**L**. ISO value of 0.02 au. Positive is purple; Negative is red.

Table S4 Orbital energies of $U^{VI}O_2$(OAc)(**L**).

Orbital	E (a.u.)	E (kcal mol^{-1})
LUMO+3 (175)	-0.0734	-46.03
LUMO+2 (174)	-0.0743	-46.60
LUMO+1 (173)	-0.0757	-47.48
LUMO (172)	-0.1182	-74.13
HOMO (171)	-0.2197	-137.78
HOMO-2 (169)	-0.2524	-158.29
HOMO-3 (168)	-0.2614	-163.94
HOMO-5 (166)	-0.2644	-165.82

5.2 Compound $U^{VI}O_2(OAc)(\mathbf{L}^{\bullet})$

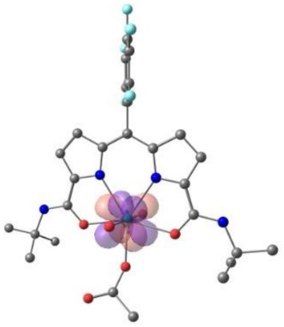

LUMO (173)

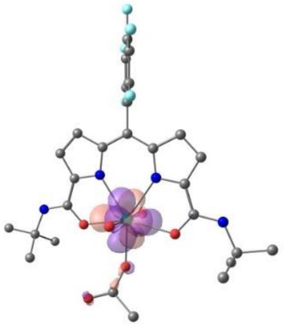

LUMO+1 (174)

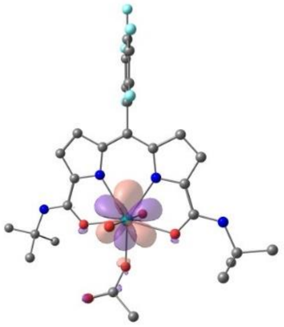

LUMO+2 (175)

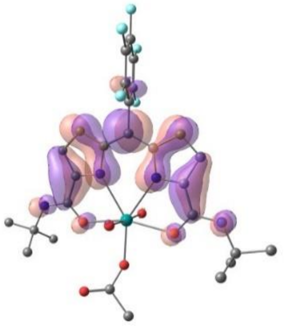

SOMO (172)

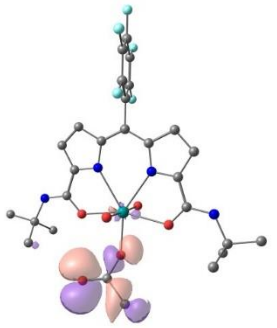

SOMO-2 (170)

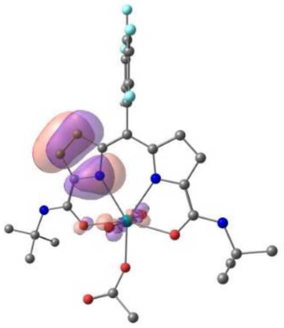

SOMO-3 (169)

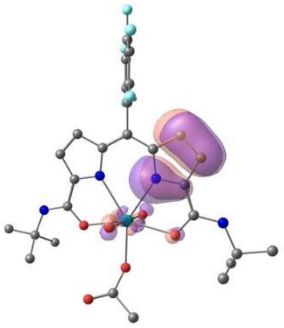

SOMO-4 (168)

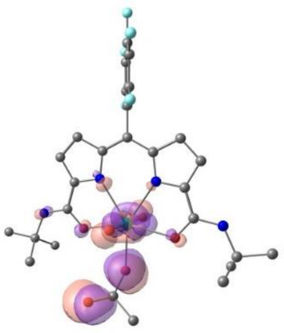

SOMO-5 (167)

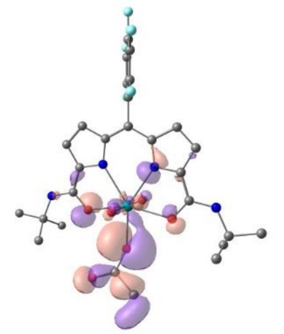

SOMO-6 (166)

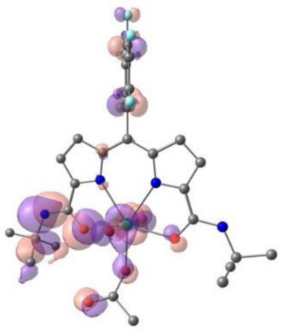

SOMO-9 (163)

Figure S24 Molecular orbital diagram of $U^{VI}O_2(OAc)(\mathbf{L}^{\bullet})$. ISO value of 0.02 au. Positive is purple; Negative is red.

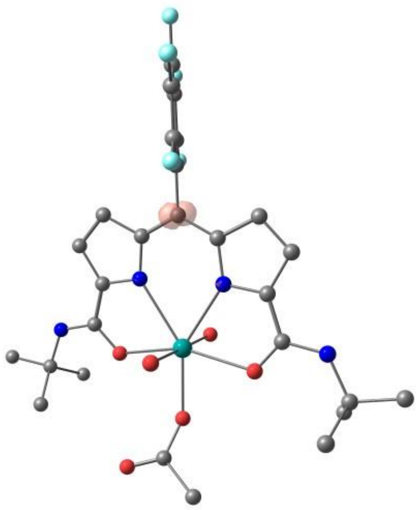

Figure S25 Unpaired spin density $U^{VI}O_2(OAc)(\mathbf{L^{\bullet}})$. Density located on the *meso*-carbon of the ligand.

Table S5 Orbital energies of $U^{VI}O_2(OAc)(\mathbf{L^{\bullet}})$.

Orbital	E (a.u.)	E (kcal mol^{-1})
LUMO+2 (175)	-0.0354	-22.20
LUMO+1 (174)	-0.0366	-22.95
LUMO (173)	-0.0378	-23.70
SOMO (172)	-0.0417	-26.15
SOMO-2 (170)	-0.1223	-76.70
SOMO-3 (169)	-0.1355	-84.98
SOMO-4 (168)	-0.1375	-86.23
SOMO-5 (167)	-0.1495	-93.76
SOMO-6 (166)	-0.1539	-96.52
SOMO-9 (163)	-0.1702	-106.74

5.3 Compound $U^{VI}O_2Cl$(**L**)

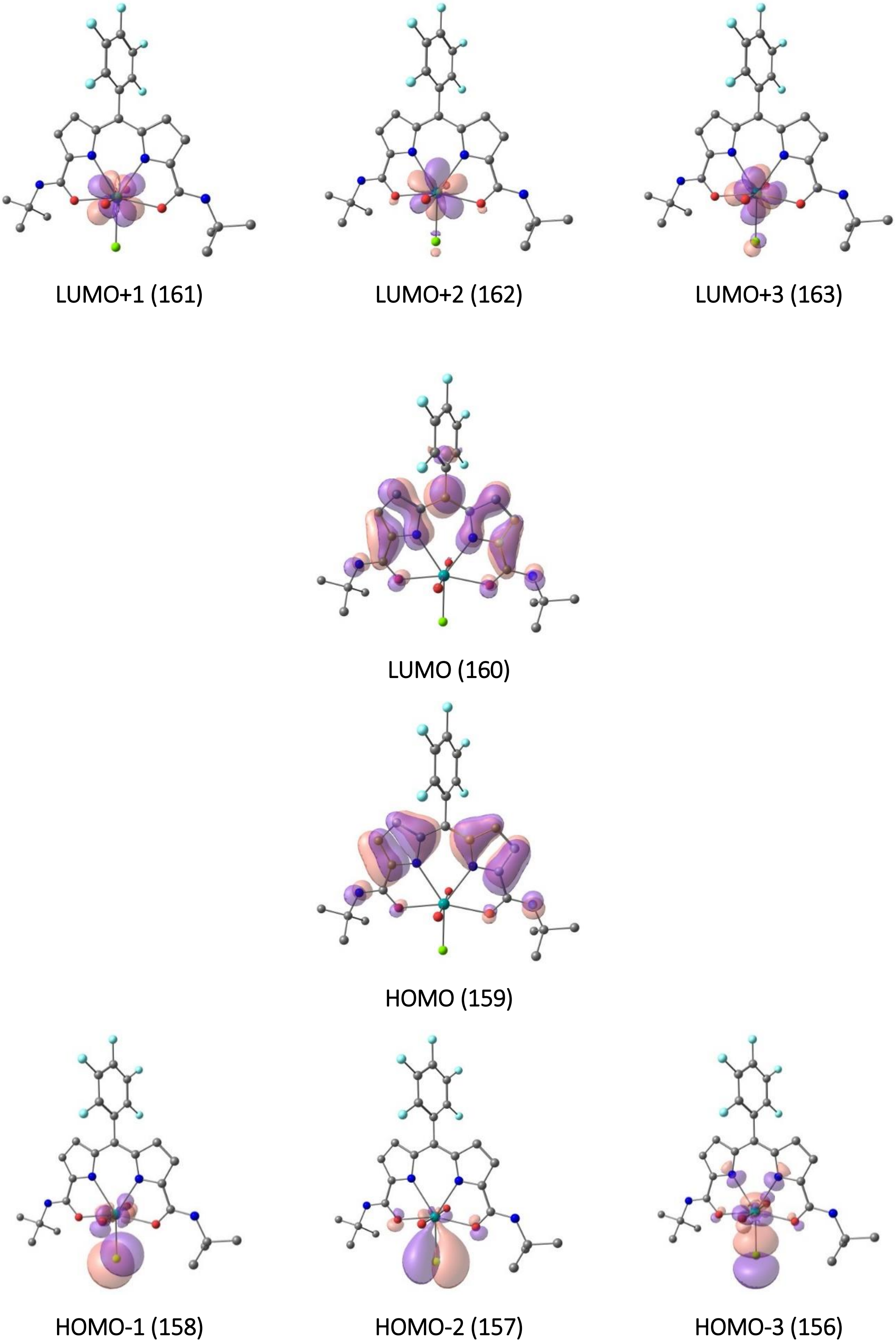

Figure S26 Molecular orbital diagram of $U^{VI}O_2Cl$**L**. ISO value of 0.02 au. Positive is purple; Negative is red.

Table S6 Orbital energies of $U^{VI}O_2Cl$(**L**).

Orbital	E (a.u.)	E (kcal mol^{-1})
LUMO+3 (163)	-0.0802	-50.30
LUMO+2 (162)	-0.0814	-51.05
LUMO+1 (161)	-0.0815	-51.11
LUMO (160)	-0.1214	-76.14
HOMO (159)	-0.2289	-143.55
HOMO-1 (158)	-0.2374	-148.89
HOMO-2 (157)	-0.2476	-155.28
HOMO-3 (156)	-0.2646	-165.88

5.4 Compound $U^{VI}O_2(Cl)(\mathbf{L}^{\bullet})$

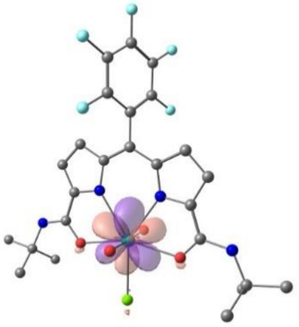

LUMO+1 (162)

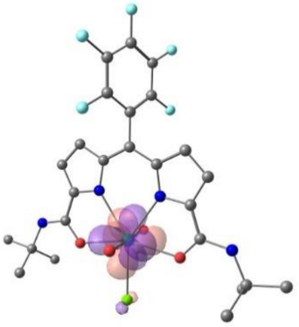

LUMO+2 (163)

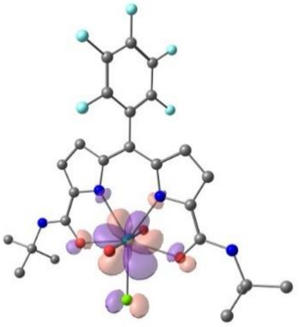

LUMO+3 (164)

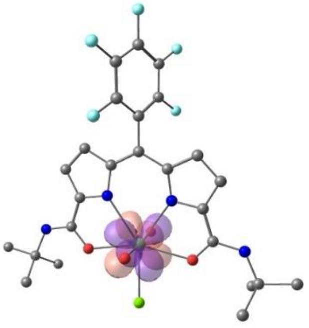

LUMO (161)

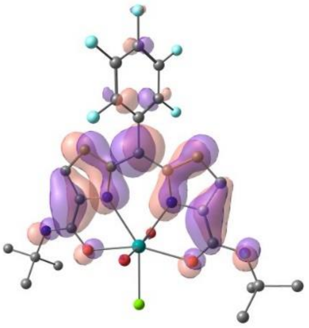

SOMO (160)

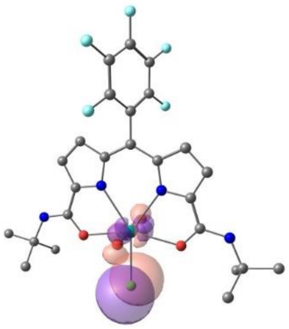

SOMO-2 (158)

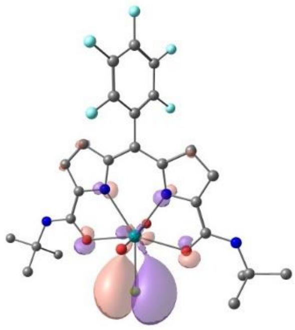

SOMO-3 (157)

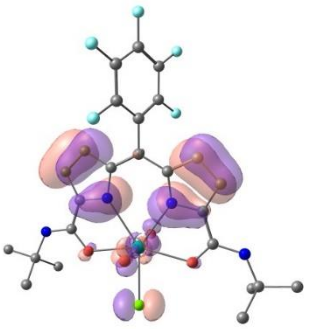

SOMO-5 (155)

Figure S27 Molecular orbital diagram of $U^{VI}O_2(Cl)(\mathbf{L}^{\bullet})$. ISO value of 0.02 au. Positive is purple; Negative is red.

Table S7 Orbital energies of $U^{VI}O_2(Cl)(\mathbf{L}^{\bullet})$.

Orbital	E (a.u.)	E (kcal mol^{-1})
LUMO+3 (164)	-0.0316	-19.82
LUMO+2 (163)	-0.0323	-20.26
LUMO+1 (162)	-0.0325	-20.38
LUMO (161)	-0.0394	-24.71
SOMO (160)	-0.0438	-27.47
SOMO-2 (158)	-0.1308	-82.03
SOMO-3 (157)	-0.1383	-86.73
SOMO-5 (155)	-0.1388	-87.05

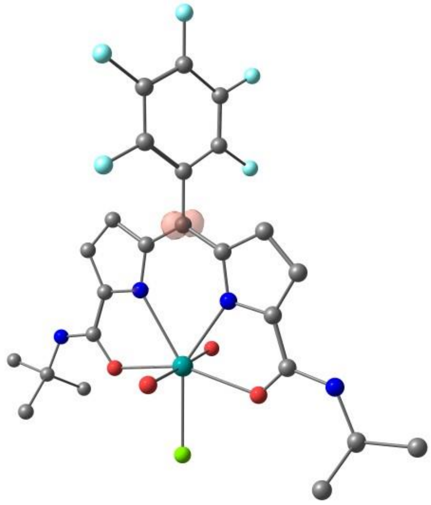

Figure S28 Unpaired spin density $U^{VI}O_2(Cl)(\mathbf{L}^{\bullet})$. Density located on the *meso*-carbon of the ligand.

Table S8 Energies of the uranyl complexes before and after one electron reduction.

Compound	H (Hartree)	H (kcal mol^{-1})	G (Hartree)	G (kcal mol$^{-1)}$
$U^{VI}O_2(OAc)(\mathbf{L})$	-2690.69	-1688433.89	-2690.82	-1688514.07
$U^{VI}O_2(OAc)(\mathbf{L}^{\bullet})$	-2690.78	-1688485.86	-2690.91	-1688567.33
$U^{VI}O_2(Cl)(\mathbf{L})$	-2477.40	-1554590.25	-2477.52	-1554664.30
$U^{VI}O_2(Cl)(\mathbf{L}^{\bullet})$	-2477.48	-1554643.92	-2477.60	-1554718.72

5.5 Cationic species

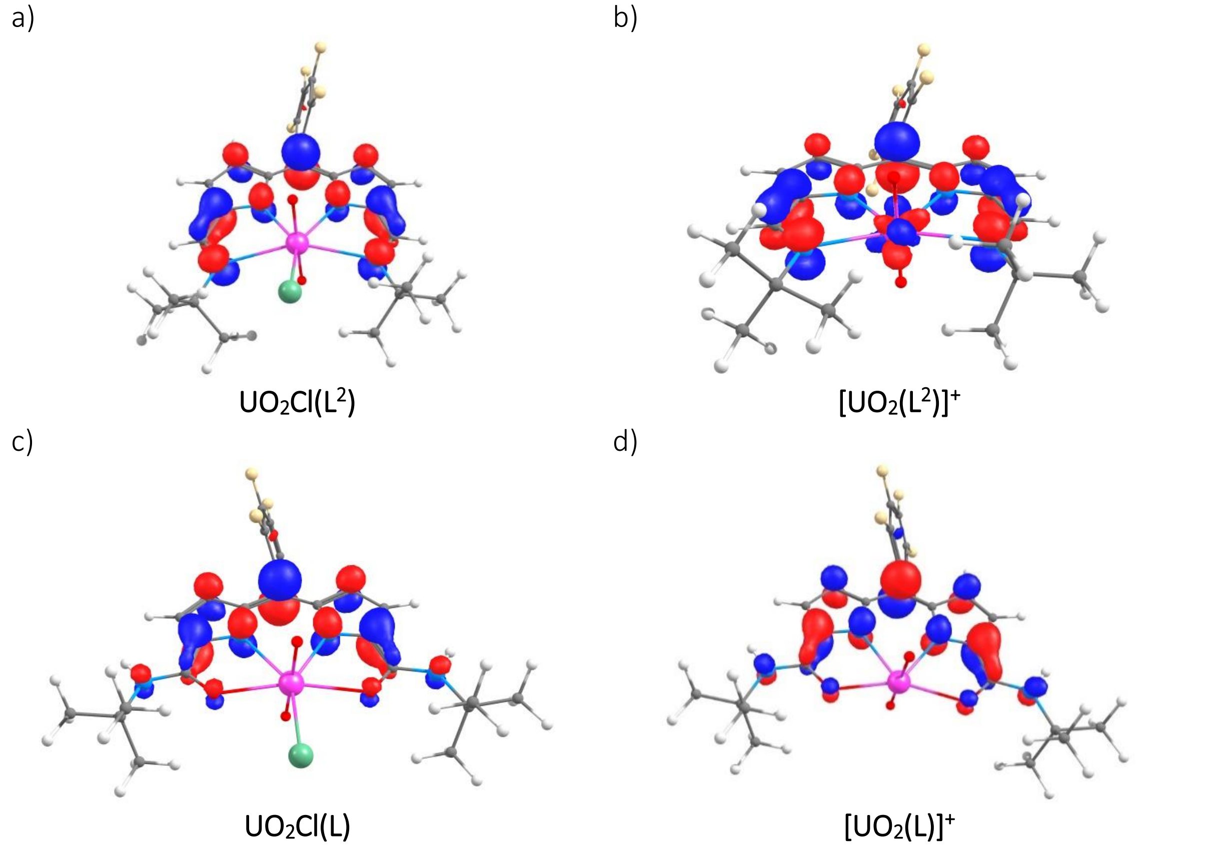

Figure S29 Molecular orbital plots of the LUMOs of a) $UO_2Cl(\mathbf{L^2})$; b) $[UO_2(\mathbf{L^2})]^+$; c) $UO_2Cl(\mathbf{L})$; d) $[UO_2(\mathbf{L})]^+$. ISO value of 0.02 au. Positive is purple; negative is red.

6 Electrochemistry

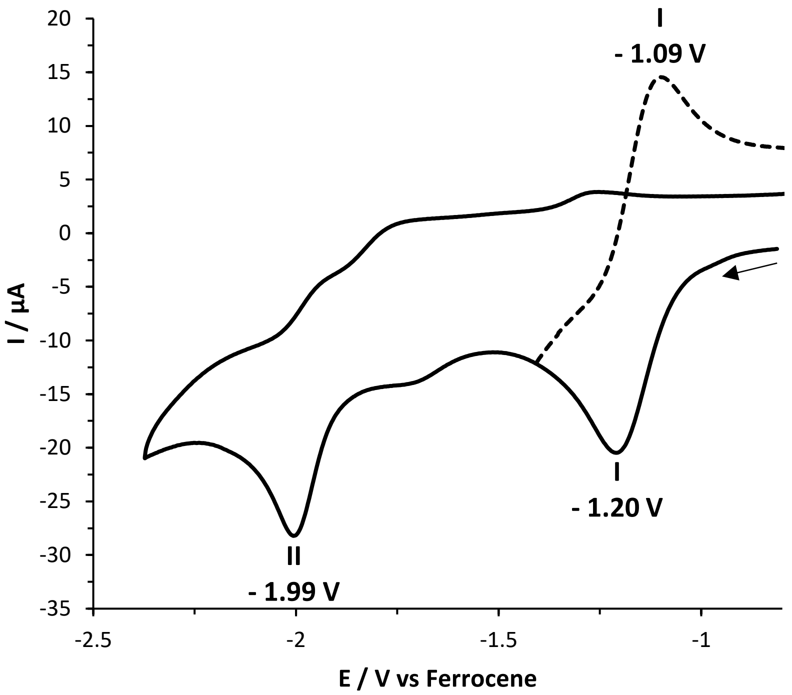

Figure S30 Cyclic voltammograms of H**L** in MeCN (1.0 mM), measured at 100 mVs^{-1} with 0.1 M [$^{n}Bu_4N$][PF_6] as electrolyte. First reduction/oxidation process isolation (dotted line).

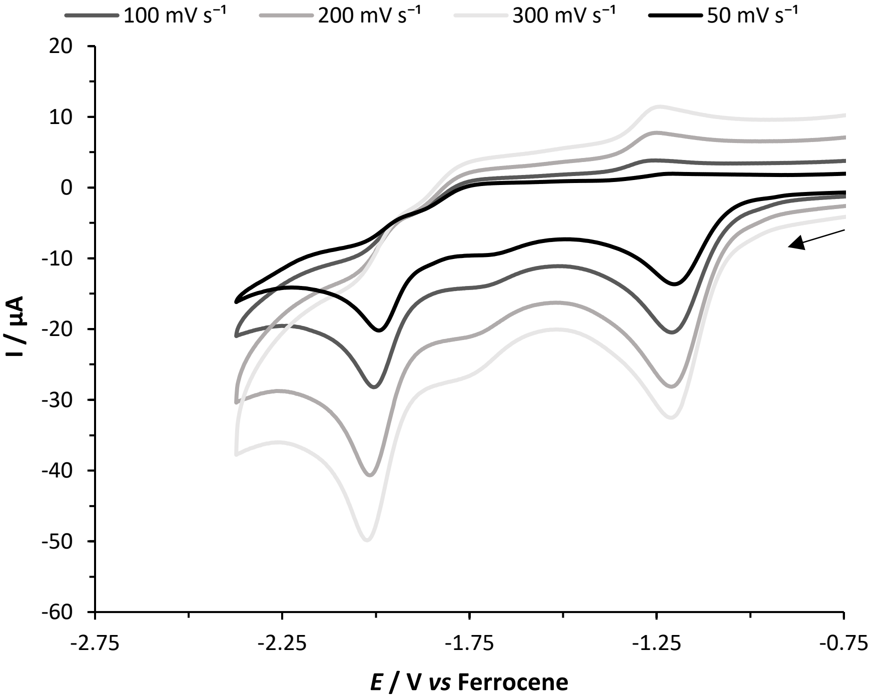

Figure S31 Cyclic voltammograms of H**L** in MeCN (1.0 mM), measured at multiple scan rates between 50 – 300 mV s^{-1} with 0.1 M [$^{n}Bu_4N$][PF_6] as electrolyte.

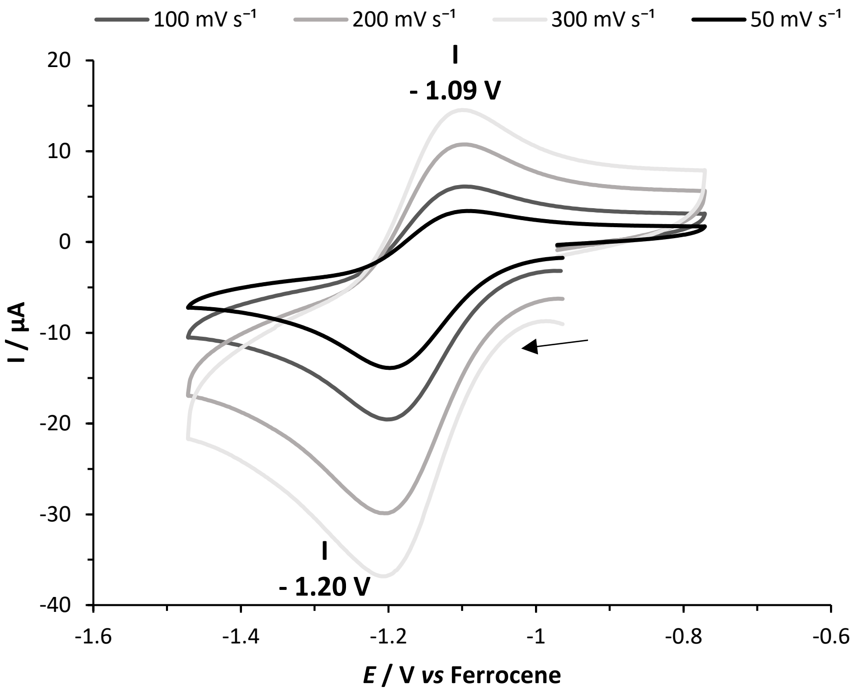

Figure S32 Cyclic voltammograms of H**L** in MeCN (1.0 mM), isolating the first process measured at multiple scan rates between 50 – 300 mV s^{-1} with 0.1 M [$^{n}Bu_4N$][PF_6] as electrolyte.

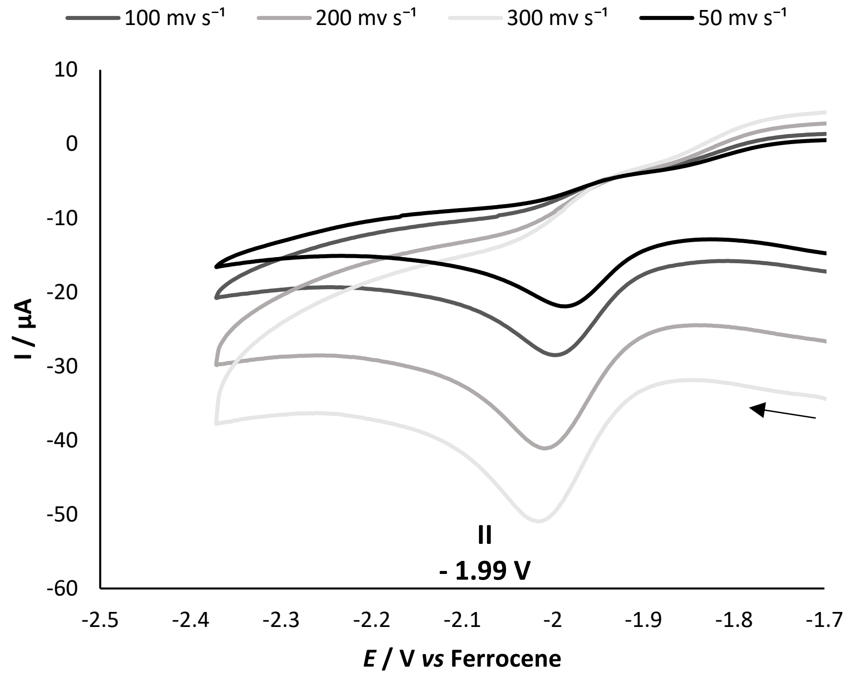

Figure S33 Cyclic voltammograms of H**L** in MeCN (1.0 mM), isolating the second process measured at multiple scan rates between 50 – 300 mV s^{-1} with 0.1 M [$^{n}Bu_4N$][PF_6] as electrolyte.

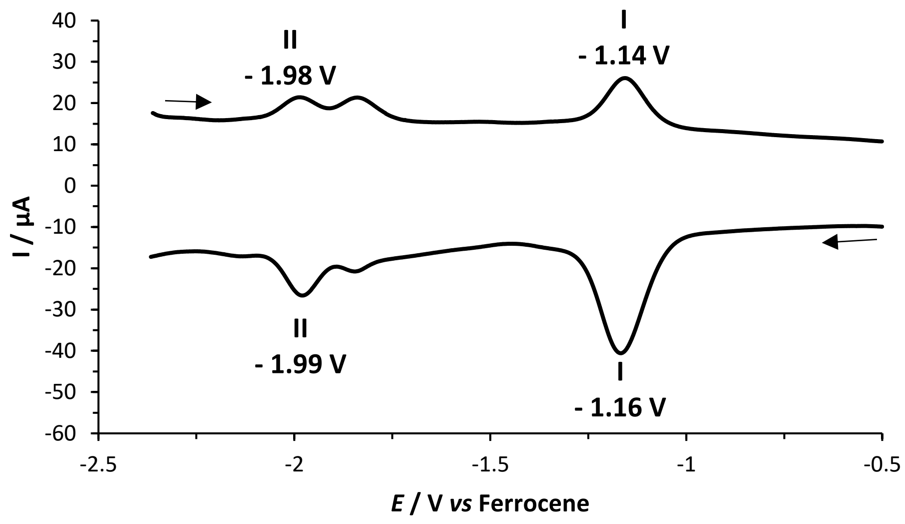

Figure S34 Square-wave voltammograms of H**L** in MeCN (1.0 mM) with 0.1 M [^{n}Bu$_4$N][PF$_6$] as electrolyte. Both the cathodic and anodic scans are shown.

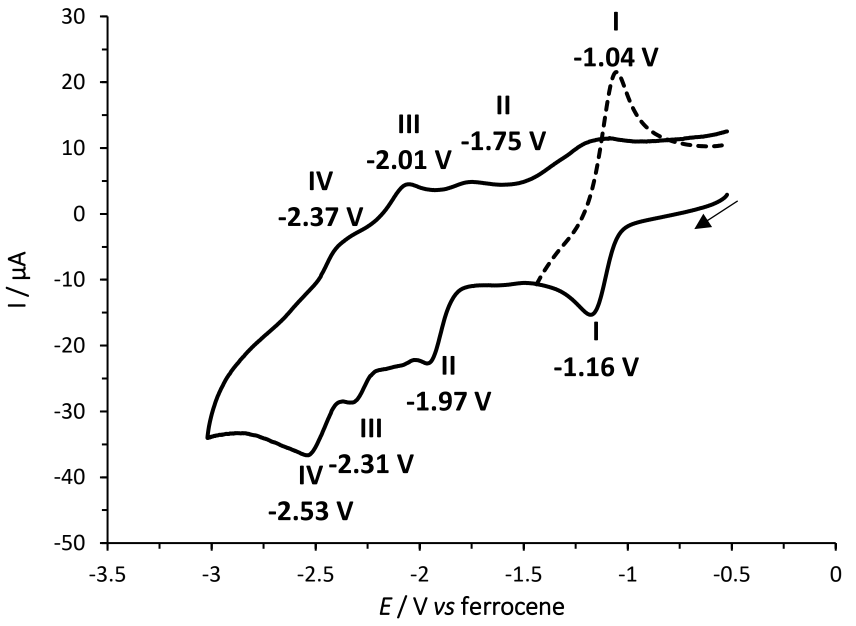

Figure S35 Cyclic voltammograms of UO_2(OAc)(**L**) in MeCN (1.0 mM), measured at 100 mVs^{-1} with 0.1 M [^{n}Bu$_4$N][PF$_6$] as electrolyte. First reduction/oxidation process isolation (dotted line).

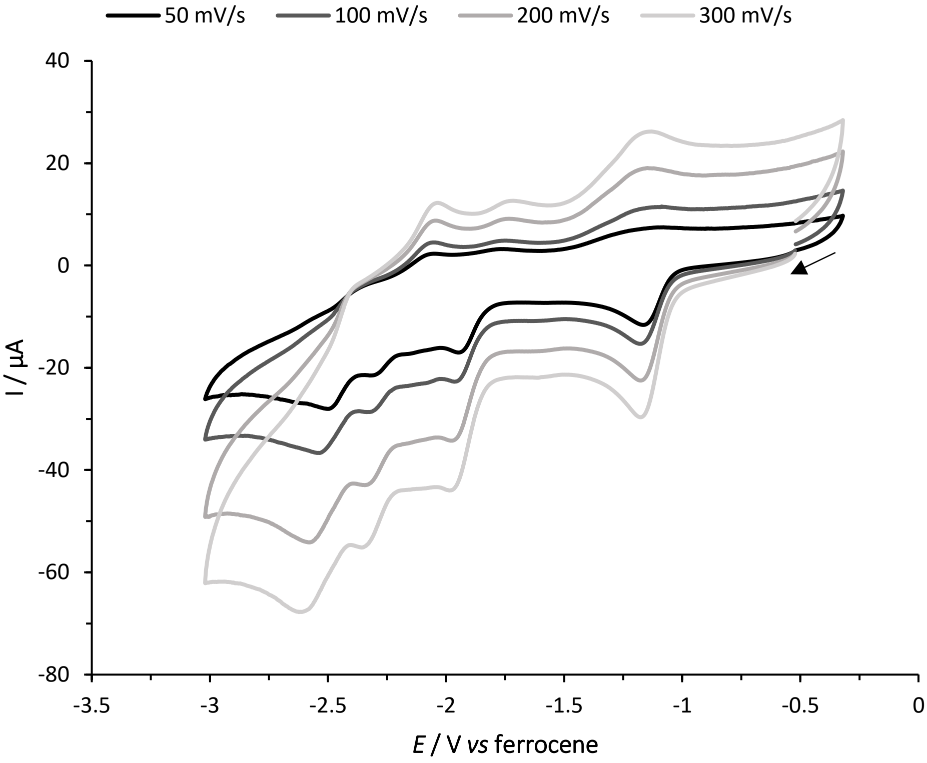

Figure S36 Cyclic voltammograms of UO_2(OAc)(**L**) in MeCN (1.0 mM), measured at multiple scan rates between 50 – 300 mV s^{-1} with 0.1 M [^{n}Bu$_4$N][PF_6] as electrolyte.

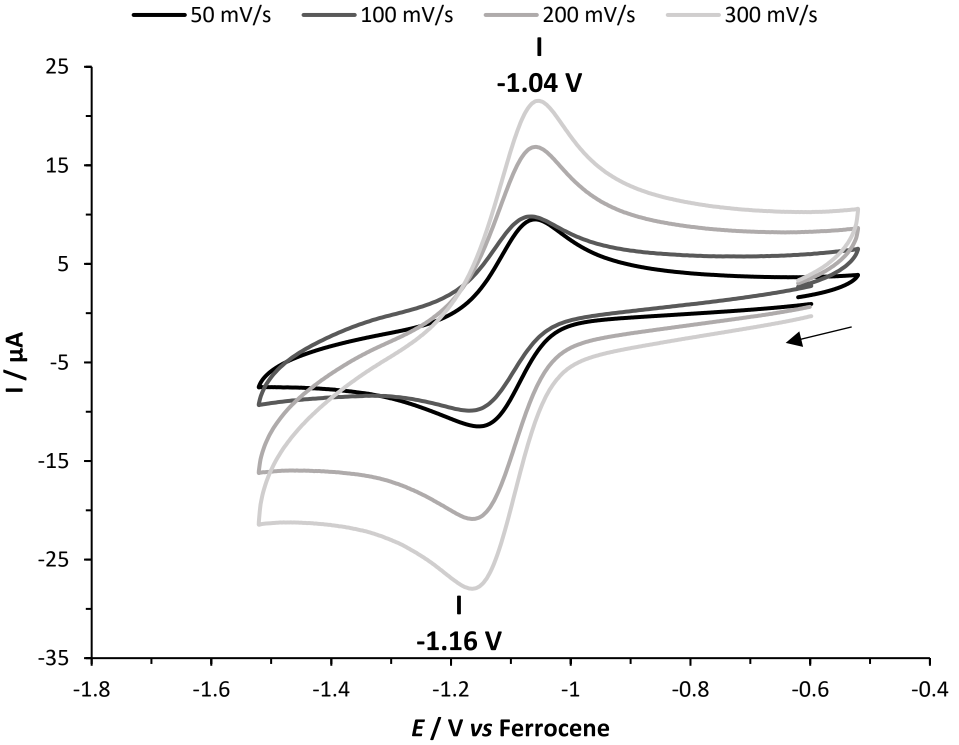

Figure S37 Cyclic voltammograms of UO_2(OAc)(**L**) in MeCN (1.0 mM), isolating the first process measured at multiple scan rates between 50 – 300 mV s^{-1} with 0.1 M [^{n}Bu$_4$N][PF_6] as electrolyte.

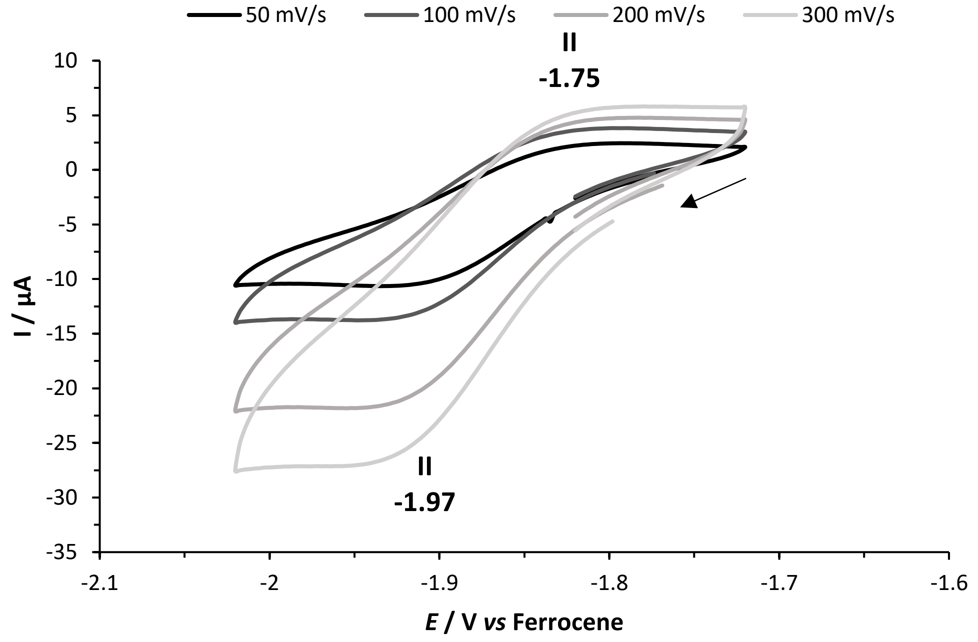

Figure S38 Cyclic voltammograms of $UO_2(OAc)$(**L**) in MeCN (1.0 mM), isolating the second process measured at multiple scan rates between 50 – 300 mV s^{-1} with 0.1 M [^{n}Bu$_4$N][PF_6] as electrolyte.

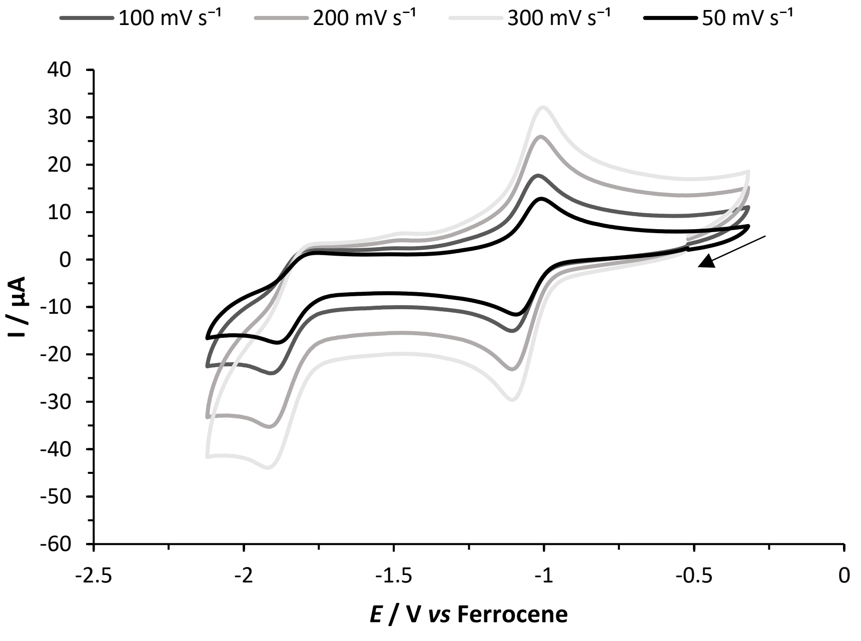

Figure S39 Cyclic voltammograms of $UO_2(OAc)$(**L**) in MeCN (1.0 mM), isolating the first and second process measured at multiple scan rates between 50 – 300 mV s^{-1} with 0.1 M [^{n}Bu$_4$N][PF_6] as electrolyte.

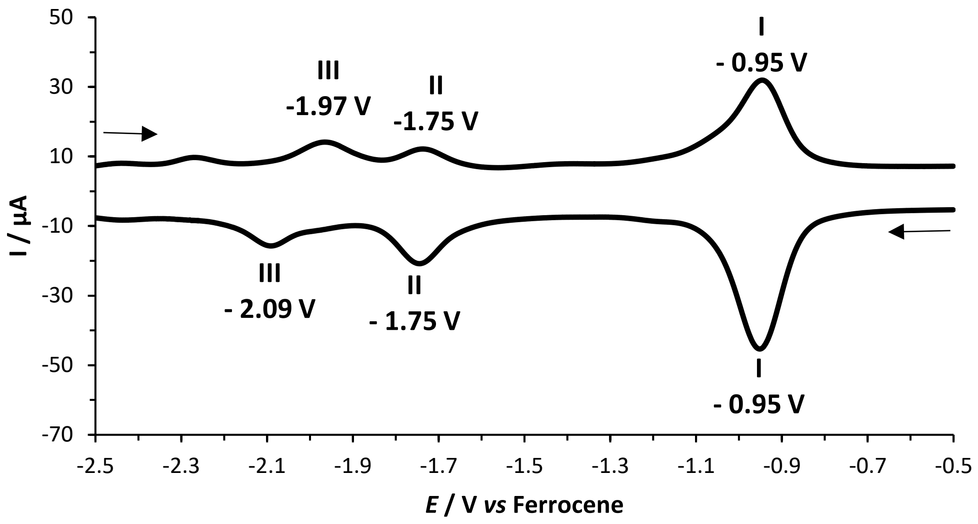

Figure S40 Square-wave voltammograms of $UO_2(OAc)$(**L**) in MeCN (1.0 mM) with 0.1 M [^{n}Bu$_4$N][PF_6] as electrolyte. Both the cathodic and anodic scans are shown.

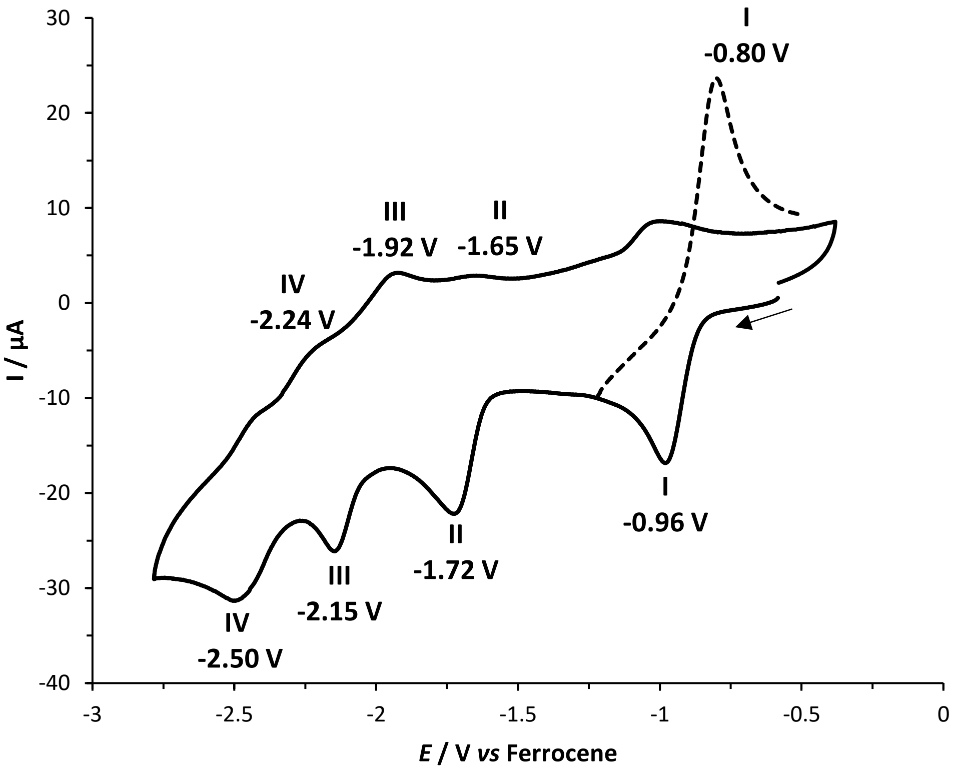

Figure S41 Cyclic voltammograms of UO_2Cl(**L**) in MeCN (1.0 mM), measured at 100 mVs^{-1} with 0.1 M [^{n}Bu$_4$N][PF_6] as electrolyte. First reduction/oxidation process isolation (dotted line).

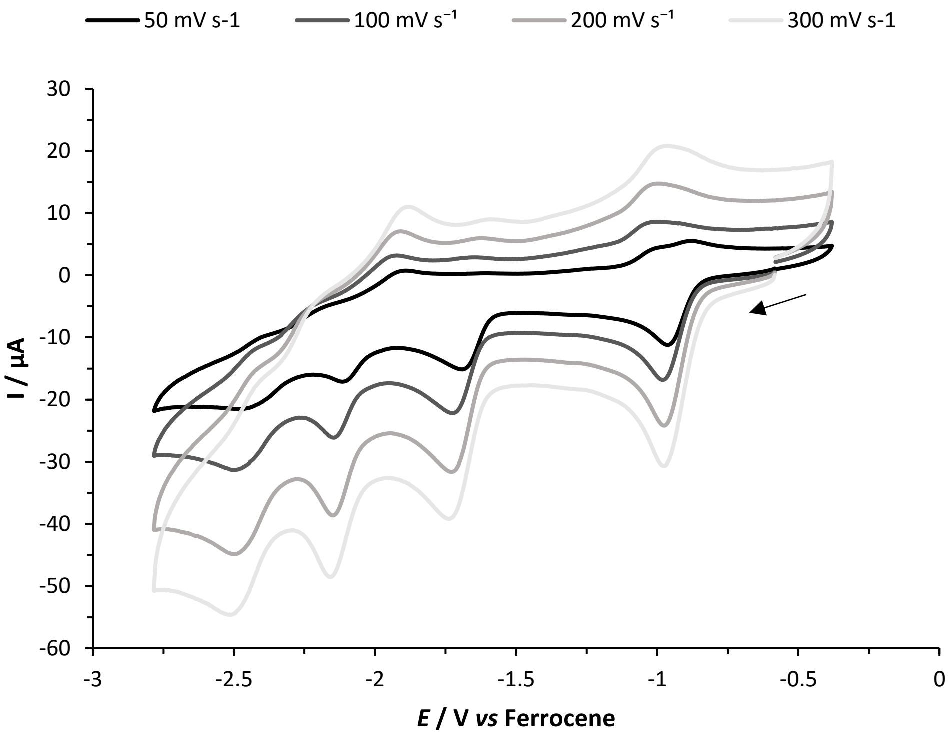

Figure S42 Cyclic voltammograms of UO_2Cl(**L**) in MeCN (1.0 mM), measured at multiple scan rates between 50 – 300 mV s^{-1} with 0.1 M [$^nBu_4N$][PF_6] as electrolyte.

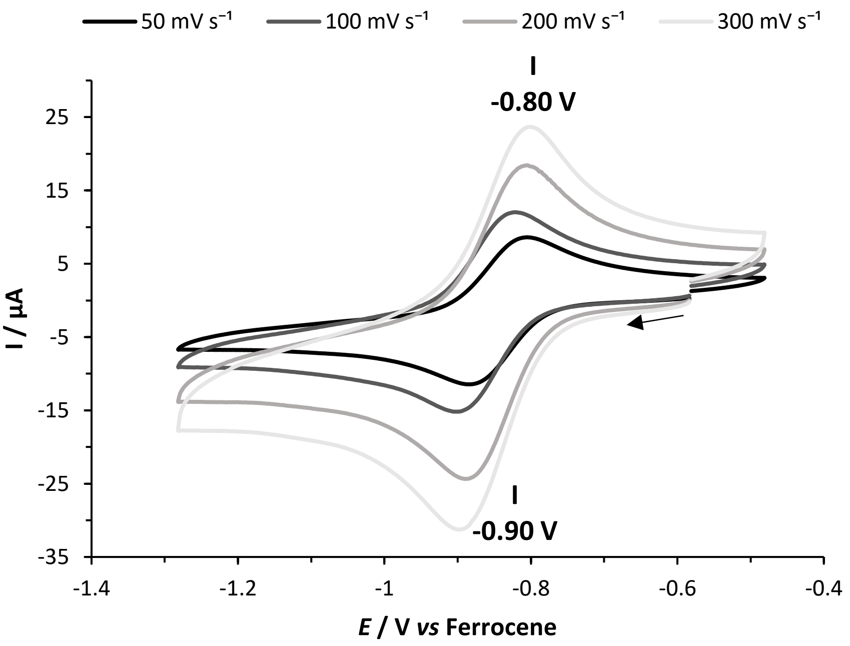

Figure S43 Cyclic voltammograms of UO_2Cl(**L**) in MeCN (1.0 mM), isolating the first process measured at multiple scan rates between 50 – 300 mV s^{-1} with 0.1 M [$^nBu_4N$][PF_6] as electrolyte.

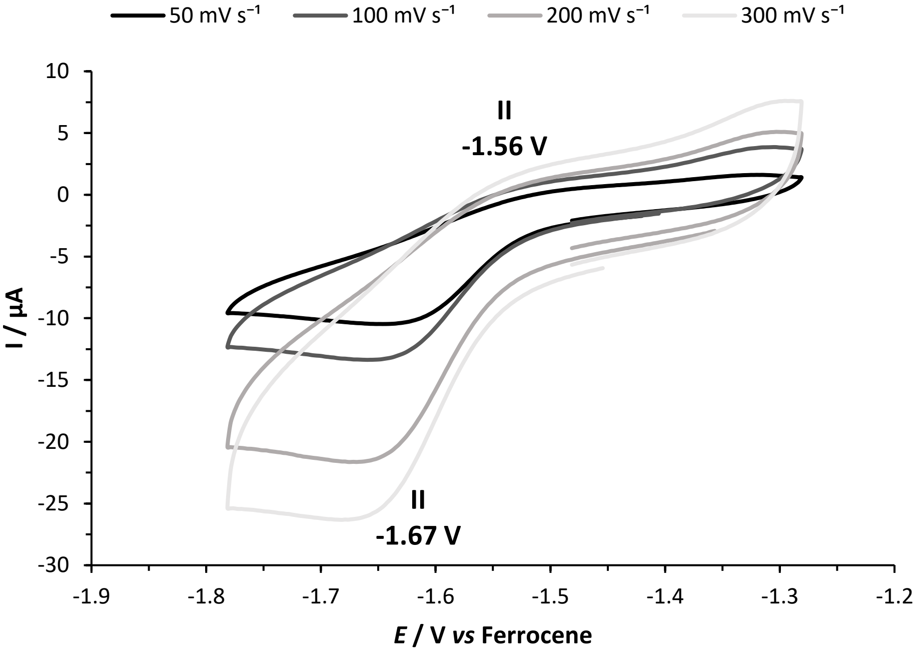

Figure S44 Cyclic voltammograms of UO_2Cl(**L**) in MeCN (1.0 mM), isolating the second process measured at multiple scan rates between 50 – 300 mV s^{-1} with 0.1 M [$^{n}Bu_4N$][PF_6] as electrolyte.

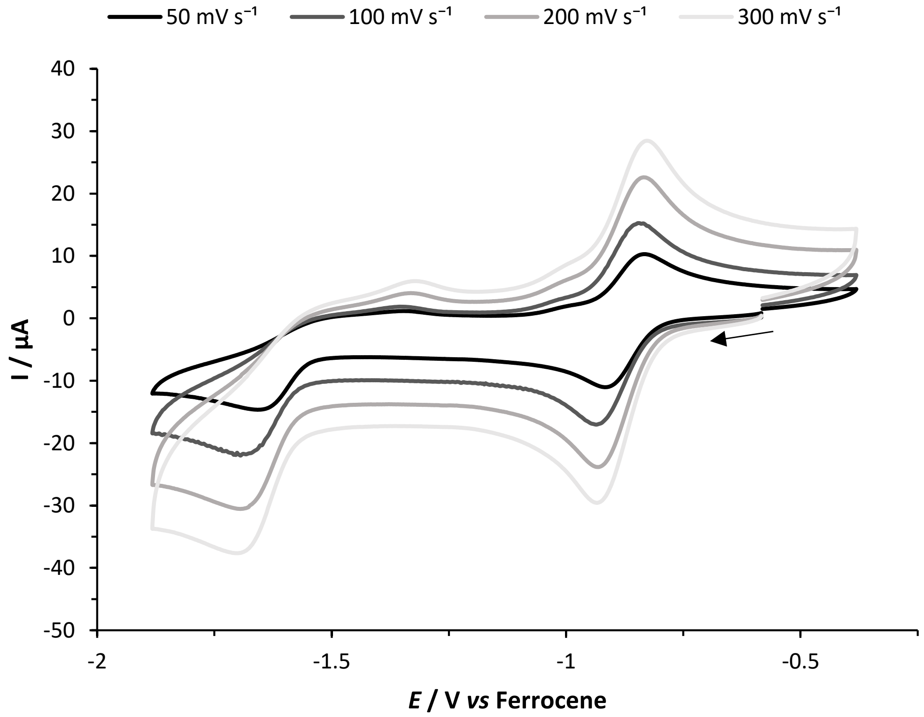

Figure S45 Cyclic voltammograms of UO_2Cl(**L**) in MeCN (1.0 mM), isolating the first and second process measured at multiple scan rates between 50 – 300 mV s^{-1} with 0.1 M [$^{n}Bu_4N$][PF_6] as electrolyte.

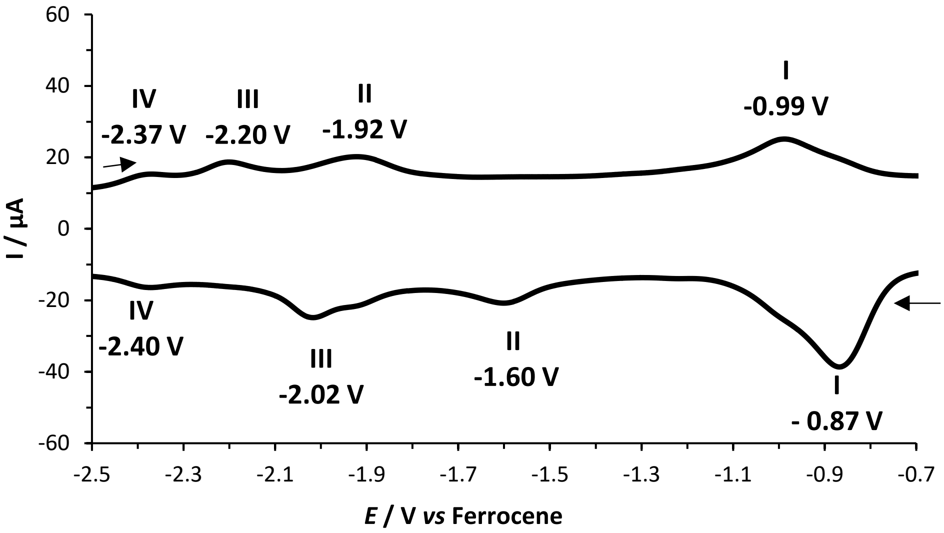

Figure S46 Square-wave voltammograms of UO_2Cl(**L**) in MeCN (1.0 mM) with 0.1 M [$^{n}Bu_4N$][PF_6] as electrolyte. Both the cathodic and anodic scans are shown.

Table S9 Summary of cyclic voltammetry data for H(**L**), UO_2(OAc)(**L**), and UO_2Cl(**L**). Values are from voltammograms measures at 100 mV s^{-1} in MeCN and all potentials are quotes *versus* ferrocene. [a] Irr. = irreversible, Q.R. = *quasi*-reversible, red. = reduction.

Compound	Process	E_p^c / V	E_p^a / V	ΔE / V	$E_{1/2}$ / V	I i_p^r/ i_p^f I	Assignment [a]
HL	I	−1.20	−1.09	0.11	−1.15	0.80	Q.R. 1e⁻ red.
	II	−1.99	-	-	-	-	Irr. 1e⁻ red.
UO_2(OAc)(L)	I	−1.16	−1.04	0.12	−1.10	1.12	Q.R. 1 e⁻ red. ligand
	II	−1.97	-	-	-	-	Irr. 1e⁻ red. U^{VI}/U^{V}
	III	−2.31	-	-	-	-	Irr. 1e⁻ red.
	IV	−2.53	-	-	-	-	Irr 1e⁻ red.
UO_2Cl(L)	I	−0.96	−0.80	0.16	−0.88	0.83	Q.R. 1 e⁻ red. ligand
	II	−1.72	-	-	-	-	Irr. 1e⁻ red. U^{VI}/U^{V}
	III	−2.15	-	-	-	-	Irr 1e⁻ red.
	IV	−2.50	-	-	-	-	Irr 1e⁻ red.

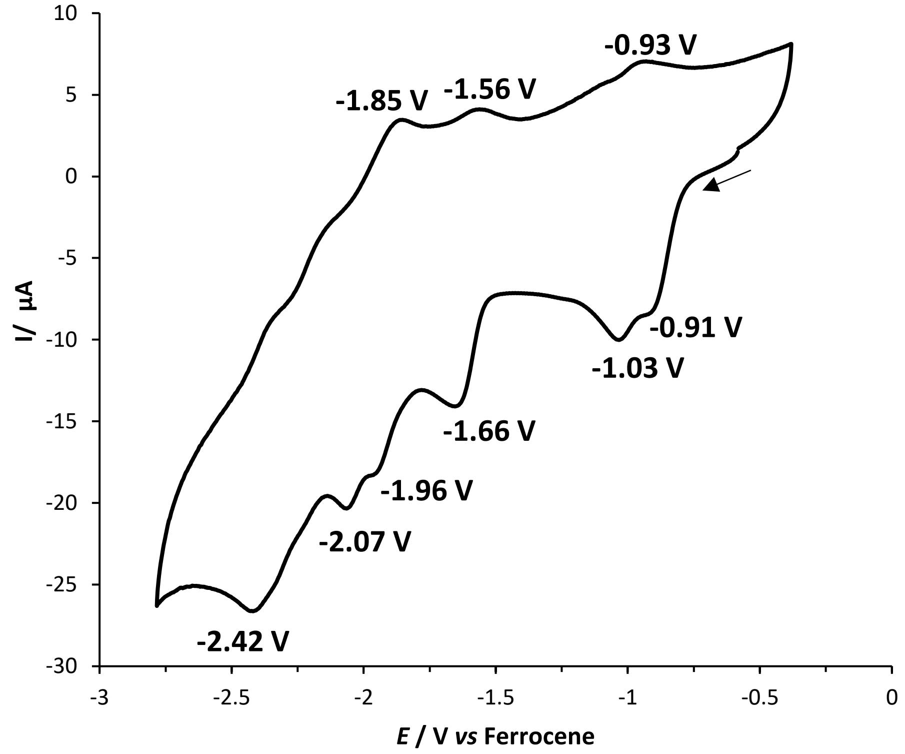

Figure S47 Cyclic voltammograms of UO_2Cl(**L**) and [UO_2(MeCN)(**L**)][Cl] in MeCN (1.0 mM), measured at 100 mVs^{-1} with 0.1 M [$^{n}Bu_4N$][PF_6] as electrolyte.

7 EPR

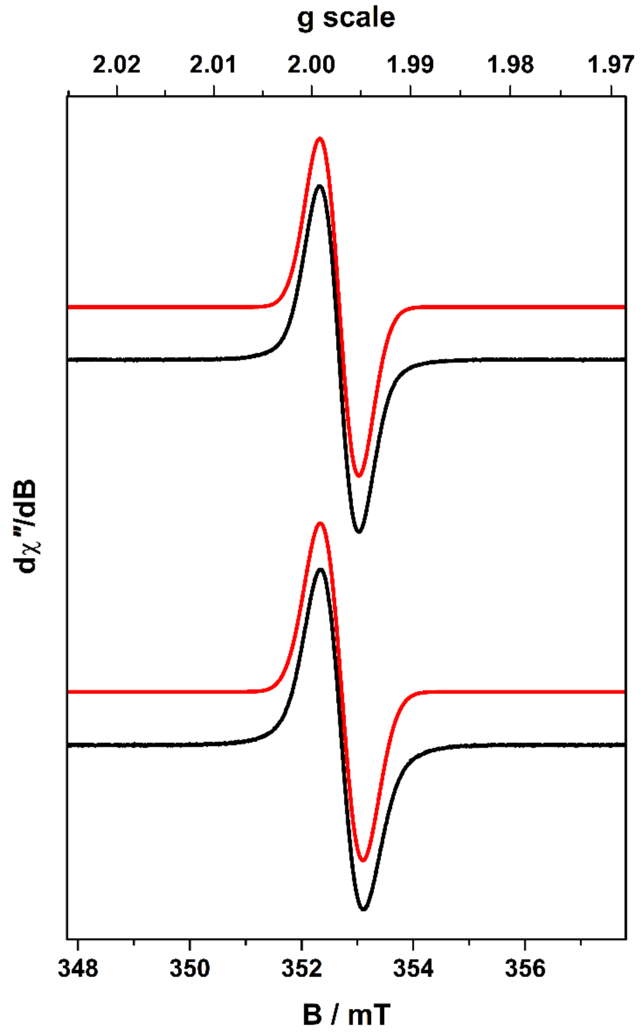

Figure S48 Comparison of the X-band EPR spectra of $UO_2(OAc)$(**L**) (top) and UO_2Cl(**L**) (bottom) recorded in CH_2Cl_2 solution at room temperature (experimental conditions: frequency, 9.858 GHZ; power, 0.63 mW; modulation, 0.02 mT). Experimental data represented by the black line; simulations are depicted by the red trace.

8 References

1. G. M. Sheldrick, *Acta Crystallogr., Sect. A: Found. Adv.*, 2015, **71**, 3−8.
2. G. M. Sheldrick, *Acta Crystallogr., Sect. C: Chem.*, 2015, **71**, 3−8.
3. G. M. Sheldrick, *Acta Crystallogr., Sect. A: Found. Adv.*, 2008, 122.
4. L. J. B. O. V. Dolomanov, R. J. Gildea, J. A. K. Howard, H. Puschmann, *J. Appl. Crystallogr.*, 2009, **42**, 339−341.
5. A. D. Becke, *J. Chem. Phys*, 1993, **98**, 5648.
6. K. Burke, J. P. Perdew and W. Yang, *Electronic Density Functional Theory: Recent Progress and New Directions*, Plenum, New york, 1998.
7. A. Moritz, X. Cao and M. Dolg, *Theor. Chem. Acc.* , 2007, **118**, 845.
8. A. Hollwarth, M. Bohme, S. Dapprich, A. W. Ehlers, A. Gobbi, V. Jonas, K. F. Kohler, R. Stegmann, A. Veldkamp and G. Frenkling, *J. Chem. Phys.*, 1993, **208**, 237.
9. P. C. Hariharan and J. A. Pople, *Theor. Chim. Acta.* , 1973, **28**, 213.
10. W. J. Hehre, R. Ditchfield and J. A. Pople, *J. Chem. Phys.*, 1972, **56**, 2257.
11. S. Grimme, S. Ehrlich and L. Goerigk, *J. Comp. Chem.*, 2011, **32**, 1456.
12. M. J. Frisch, G. W. Trucks, H. B. Schlegel, G. E. Scuseria, M. A. Robb, J. R. Cheeseman, G. Scalmani, V. Barone, G. A. Petersson, H. Nakatsuji, X. Li, M. Caricato, A. V. Marenich, J. Bloino, B. G. Janesko, R. Gomperts, B. Mennucci, H. P. Hratchian, J. V. Ortiz, A. F. Izmaylov, J. L. Sonnenberg, Williams, F. Ding, F. Lipparini, F. Egidi, J. Goings, B. Peng, A. Petrone, T. Henderson, D. Ranasinghe, V. G. Zakrzewski, J. Gao, N. Rega, G. Zheng, W. Liang, M. Hada, M. Ehara, K. Toyota, R. Fukuda, J. Hasegawa, M. Ishida, T. Nakajima, Y. Honda, O. Kitao, H. Nakai, T. Vreven, K. Throssell, J. A. Montgomery Jr., J. E. Peralta, F. Ogliaro, M. J. Bearpark, J. J. Heyd, E. N. Brothers, K. N. Kudin, V. N. Staroverov, T. A. Keith, R. Kobayashi, J. Normand, K. Raghavachari, A. P. Rendell, J. C. Burant, S. S. Iyengar, J. Tomasi, M. Cossi, J. M. Millam, M. Klene, C. Adamo, R. Cammi, J. W. Ochterski, R. L. Martin, K. Morokuma, O. Farkas, J. B. Foresman and D. J. Fox, *Journal*, 2016.
13. G. R. Hanson, K. E. Gates, C. J. Noble, M. Griffin, A. Mitchell and S. Benson, *Journal of Inorganic Biochemistry*, 2004, **98**, 903-916.

14 . (a) A. D. Becke, *J. Chem. Phys*. **1993**, *98*, 5648; (b) K. Burke, J. P. Perdew, W. Yang, in Electronic Density Functional Theory: Recent Progress and New Directions, Eds: J. F. Dobson, G. Vignale, M. P. Das, Plenum, New York, 1998

15 (a) A. Moritz, X. Cao and M. Dolg, *Theor. Chem. Acc*. **2007**, *118*, 845; (b) A. Hollwarth, M. Bohme, S. Dapprich, A.W. Ehlers, A. Gobbi, V. Jonas, K.F. Kohler, R. Stegmann, A. Veldkamp, G. Frenking *J. Chem. Phys*. **1993**, *208*, 237.

16. (a) P. C. Hariharan and J. A. Pople, Theor. Chim. Acta 1973, 28, 213; (b) W. J. Hehre, R. Ditchfield and J. A. Pople, *J. Chem. Phys*. **1972**, *56*, 2257.

17 S. Grimme, S. Ehrlich, L. Goerigk, *J. Comp. Chem.*, **2011**, *32*, 1456

18. Gaussian 16, Revision B.01, M. J. Frisch, G. W. Trucks, H. B. Schlegel, G. E. Scuseria, M. A. Robb, J. R. Cheeseman, G. Scalmani, V. Barone, G. A. Petersson, H. Nakatsuji, X. Li, M. Caricato, A. V. Marenich, J. Bloino, B. G. Janesko, R. Gomperts, B. Mennucci, H. P. Hratchian, J. V. Ortiz, A. F. Izmaylov, J. L. Sonnenberg, D. Williams-Young, F. Ding, F. Lipparini, F. Egidi, J. Goings, B. Peng, A. Petrone, T. Henderson, D. Ranasinghe, V. G. Zakrzewski, J. Gao, N. Rega, G. Zheng, W. Liang, M. Hada, M. Ehara, K. Toyota, R. Fukuda, J. Hasegawa, M. Ishida, T. Nakajima, Y. Honda, O. Kitao, H. Nakai, T. Vreven, K. Throssell, J. A. Montgomery, Jr., J. E. Peralta, F. Ogliaro, M. J. Bearpark, J. J. Heyd, E. N.

Brothers, K. N. Kudin, V. N. Staroverov, T. A. Keith, R. Kobayashi, J. Normand, K. Raghavachari, A. P. Rendell, J. C. Burant, S. S. Iyengar, J. Tomasi, M. Cossi, J. M. Millam, M. Klene, C. Adamo, R. Cammi, J. W. Ochterski, R. L. Martin, K. Morokuma, O. Farkas, J. B. Foresman, and D. J. Fox, Gaussian, Inc., Wallingford CT, 2016.
